# Supplementary material for: Polarization of Myosin II Refines Tissue Material Properties to Buffer Mechanical Stress
Source: Dev Cell. 2019 Jan 28;48(2):245–260.e7. doi: 10.1016/j.devcel.2018.12.020 (PMC6353629; doi:10.1016/j.devcel.2018.12.020)
Supplement: Document S1. Figures S1–S7 [file mmc1.pdf]

Developmental Cell, Volume *48*

Supplemental Information

Polarization of Myosin II Refines Tissue Material Properties to Buffer Mechanical Stress

Maria Duda, Natalie J. Kirkland, Nargess Khalilgharibi, Melda Tozluoglu, Alice C. Yuen, Nicolas Carpi, Anna Bove, Matthieu Piel, Guillaume Charras, Buzz Baum, and Yanlan Mao

Supplemental Information

INVENTORY OF SUPPLEMENTAL INFORMATION

Supplemental Data

Figure S1, related to Figure 1
Figure S2, related to Figure 2
Figure S3, related to Figure 4
Figure S4, related to Figure 4
Figure S5, related to Figure 5
Figure S6, related to Figure 6
Figure S7, related to Figure 6 and 7

Supplemental Movies Legends

Movie S1, related to Star Methods: Young's modulus determination
Movie S2, related to Figure 1
Movie S3, related to Figure 2
Movie S4, related to Figure 6
Movie S5, related to Figure 6
Movie S6, related to Figure 4

Duda et al. Fig. S1 related to Figure 1

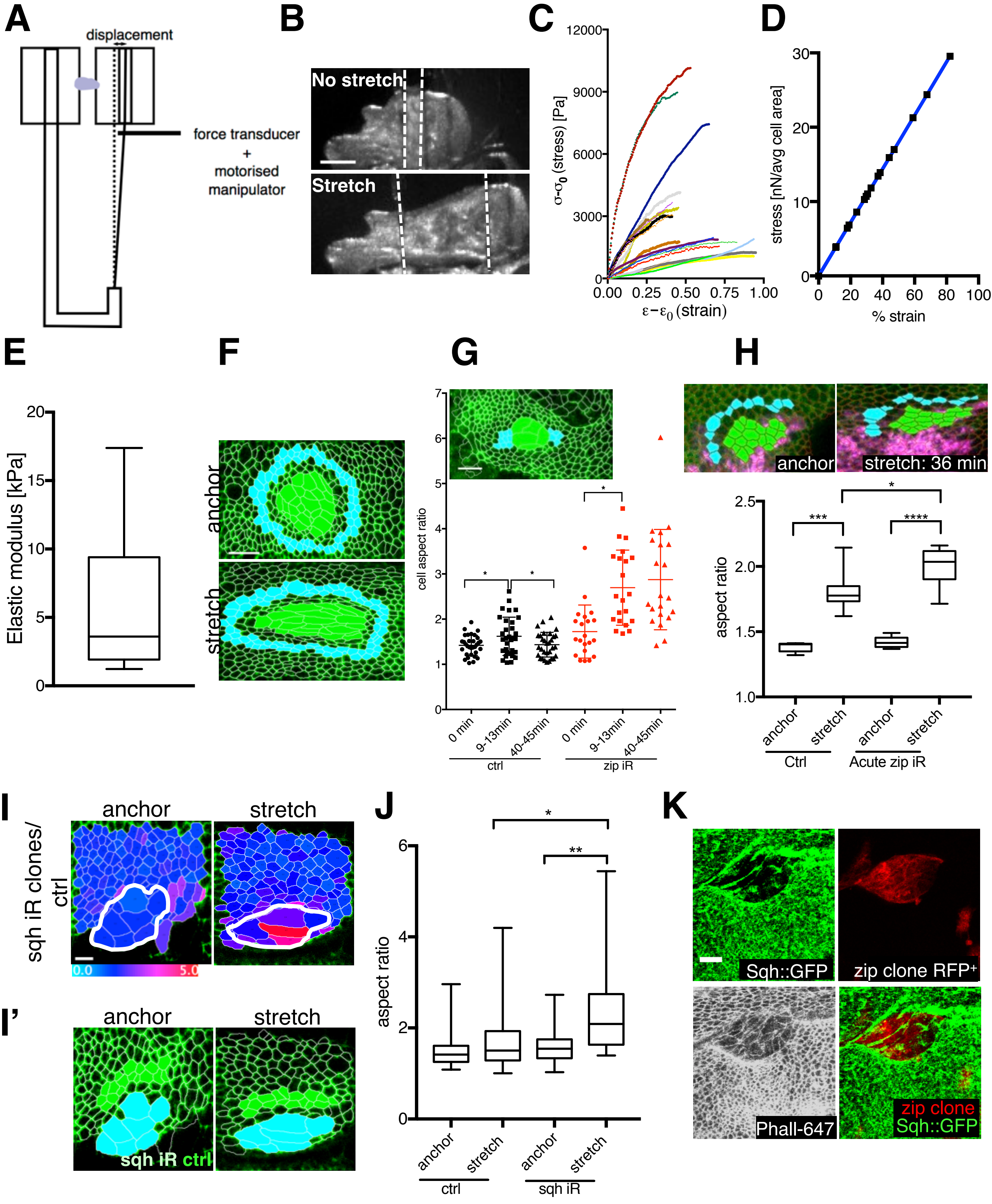

Figure S1, related to Figure 1. Further characterization of forces applied and *zip* RNAi cells in stretching assays.

(A) Schematics of the force transducer set up: wing (purple) is attached to two coverslips glued on a stiff and elastic rod. The elastic rod is directly connected to the force transducer and motorised manipulator, which stretches the tissue.

(B) Brightfield image of wing disc prior to and after stretch.

(C) Stress (y axis)-strain (x axis) curves of each stretching experiment. N=15 discs, 3 different devices (as shown in A).

(D) Forces experienced by the average cell area (10 μm^2) with different degrees of stretching (quantified as % strain change; strains are comparable to assay shown in (C).

(E) Box plot showing distribution of derived Young's modulus values; median (3.6.kPa) is indicated with horizontal line and used thereafter for force calculations.

(F) Segmentation example of a *zip*-RNAi clone as in Fig. 1E. Selection of control (cyan) and *zip*-RNAi (green) cells. Note a 2-cell rows gap is always ensured between clone and control cells in order to avoid the unspecific effects from clone stretching.

(G) Distribution plot showing aspect ratio change in discs subjected to constant deformation for 40-45 min for control and *zip*-RNAi cells (image shows selection of control cells (cyan) directly adjacent and in series with the *zip*-RNAi clone (green) and aligned with direction of the stretch); X-axis defines specific timepoints during stretch (in minutes); 0 min indicates anchored (unstretched) discs. N=3 discs; error bars represent S.D.

(H) Silent clone system with 16 hours *zip*-RNAi expression (marked by magenta) in third instar wing discs expressing Arm::GFP subject to up to 40 min stretch. Overlaid segmentation with control (cyan) and *zip*-RNAi (green). Box plot showing distribution of cell aspect ratio in anchored and stretched (20 min) discs with acute *zip*-RNAi; median is represented by horizontal line; 75th and 25th centiles are represented by top and bottom of the boxes respectively, n= 5-7 wing discs.

(I) Color coded aspect ratio of anchored and stretched (20 min) disc with *sqh*-RNAi clone (labeled as *sqh* iR and outlined in white).

(I') Control cells (green) and *sqh*-RNAi cells (cyan) prior to and after stretch (20 min).

(J) Box plot showing distribution of cell aspect ratio in anchored and stretched (20 min) wing disc for control (wild-type) and *sqh*-RNAi cells; median is represented by horizontal line; 75th and 25th centiles are represented by top and bottom of the boxes respectively, n= 3 wing discs.

(K) Reduction of Sqh::GFP signal in *zipper* RNAi clone (labeled as *zip* iR, in red).

*p<0.05, **p<0.01 with *t*-test. Scale bars, 5 μm (K), 10 μm (F,G,I,I'), 150 μm (B).

Duda et al. Fig. S2 related to Figure 2

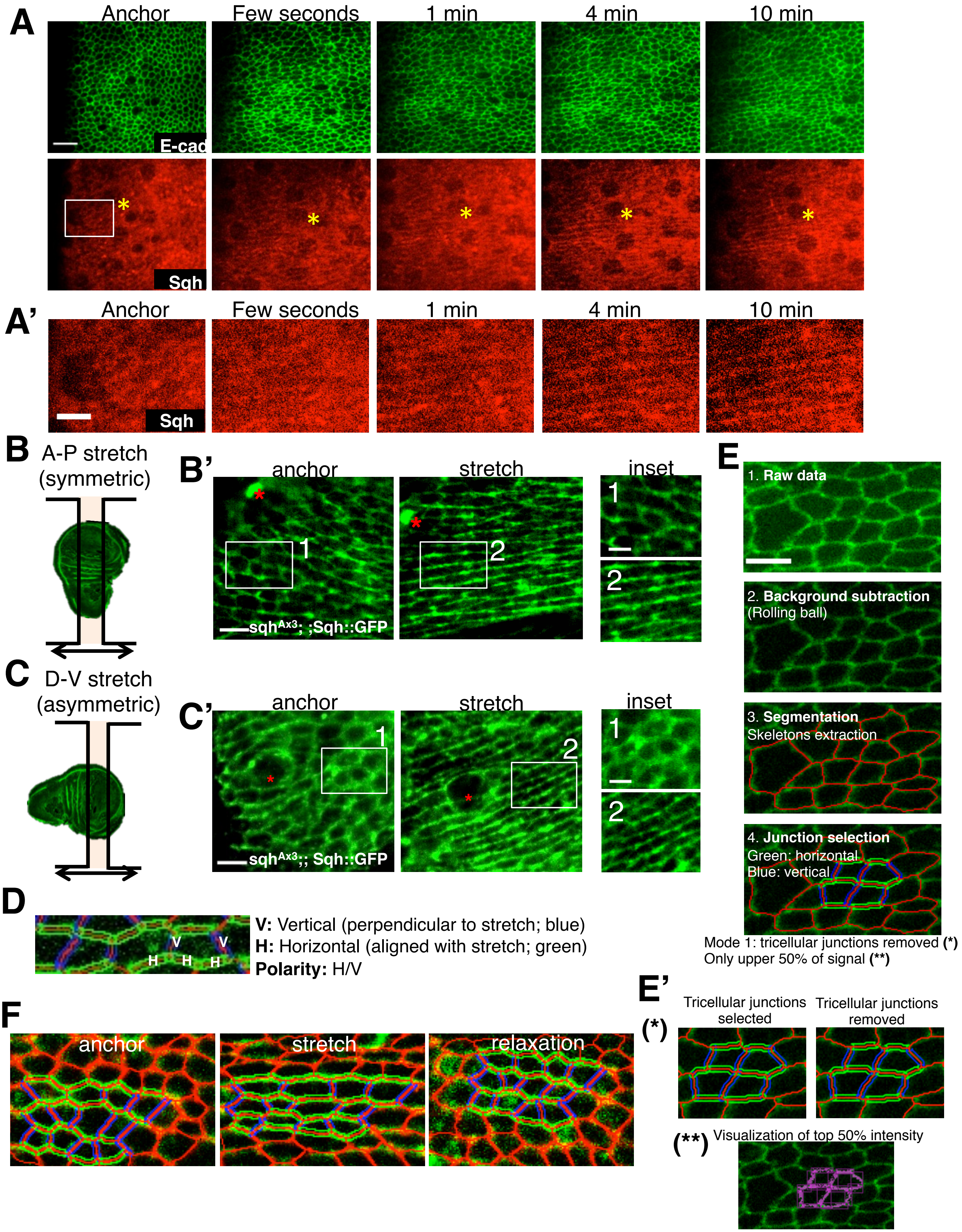

Figure S2, related to Figure 2. Emergence of Sqh polarity and Quantification of polarity.

(A) Emergence of Myosin polarity following mechanical stretching in discs expressing Sqh:mCherry and E-cad::GFP. (A') Inset of Sqh::mCherry from experiment described in (A).

(B) Schematics showing symmetric stretching, defined as stretch along anterior (A)-posterior (P) axis. (B') Discs expressing Sqh::GFP in *sqh*^{Ax3} null background subjected to symmetric stretch. Insets show a close up of equivalent regions prior to (1) and after stretch (2).

(C) Schematics showing asymmetric stretching, defined as stretch along dorsal (D)-ventral (V) axis. (C') Sqh::GFP in *sqh*^{Ax3} null background subjected to asymmetric stretch. Insets show a close up of equivalent regions prior to (1) and after stretch (2).

(D) Example of vertical (blue) and horizontal junctions (green). Vertical junctions are at angle > 45° to the direction of stretch while horizontal junctions are aligned <45° with respect to the direction of stretch (and equivalent axis in unstretched tissue). Polarity is defined as the enrichment of mean intensity on horizontal junctions with respect to mean intensity on vertical junctions.

(E) Steps of fluorescent intensity measurements: raw data is subjected to background subtraction and segmentation with EpiTools, junctions are categorized as vertical or horizontal (if unsure, junction omitted) and pixels in the 50% top intensity (**) are measured in mode 1 (tricellular signal removed (*)); note that extracted intensity overlaps perfectly with junctional geometry.

(F) Example of junction selection in anchored, stretched and relaxed disc; green junctions are defined as horizontal junctions, blue junctions are vertical junctions.

Red and yellow asterisks indicate equivalent regions in the tissue. Scale bars, 10 μm (A), 5 μm (A',B',C', E), 3 μm (B' inset, C' inset).

Duda et al. Fig. S3 related to Figure 4

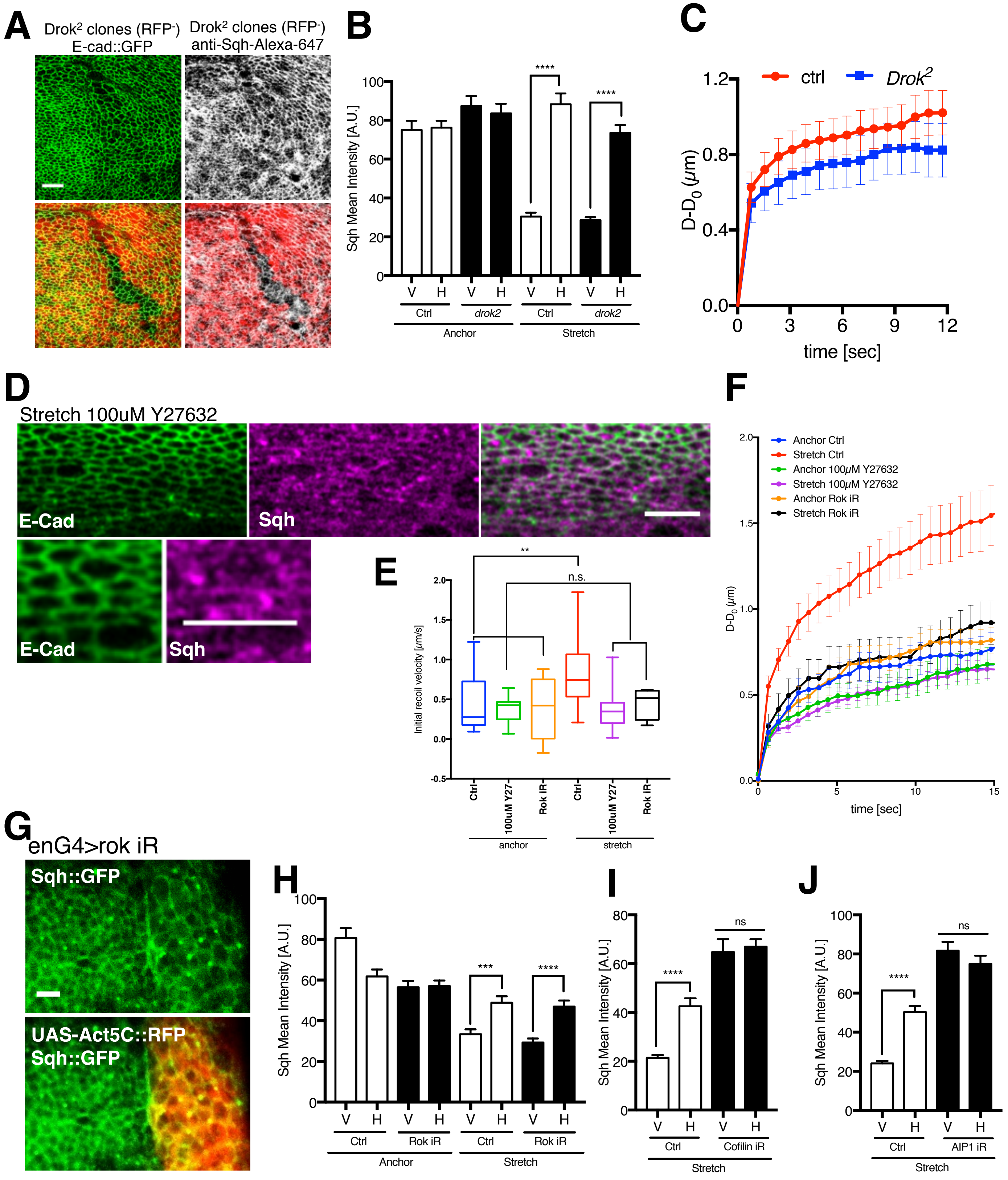

Figure S3, related to Figure 4. Characterization of *Rok* depletion.

(A) *Drok*^{2} clones in Arm::GFP expressing wing disc stained with anti - Sqh (Alexa 647, red) antibody showing enlarged cell size and junctional Sqh inside the clone.

(B) Quantification of junctional Sqh mean intensity in experiment (A) for anchor and stretch, control and *Drok*^{2}; error bars represent S.E.M.

(C) Laser ablations of junctions in *Drok*^{2} clones and control cells in Arm::GFP disc. Plot shows increase in distance between recoiling vertices [μm] against time [sec.]; error bars represent S.E.M.

(D) Rok inhibitor Y27632 treated discs expressing ECad::GFP and Sqh::Cherry under stretch.

(E) Single junction ablation initial recoil velocity for Rok inhibitor treated and *rok*-RNAi wing discs; error bars represent S.E.M.

(F) Laser ablations of junctions used for calculation of (E). Plot shows increase in distance between recoiling vertices [μm] against time [sec.]; error bars represent S.E.M.

(G) UAS- *rok*-RNAi expressed from enGal4 driver (posterior side marked by UAS-Act5C::RFP in red) in Sqh::GFP wing disc shows junctional Sqh as well as enlarged cell size of *rok* iR cells.

(H) Quantification of Sqh junctional intensity for experiment described for (G) in anchored and stretched discs; error bars represent S.E.M.

(I) Quantification of stretched discs expressing Sqh::GFP and *enGal4>UAS-cofilin RNAi* and *UAS-Act5c-RFP*, presented in FigS4C; error bars represent S.E.M.

(J) Quantification of stretched discs expressing Sqh::GFP and *enGal4>UAS-AIP1 RNAi* and *UAS-Act5c-RFP*, presented in FigS4C; error bars represent S.E.M.

Scale bars 10 μm.

Duda et al. Fig. S4 related to Figure 4

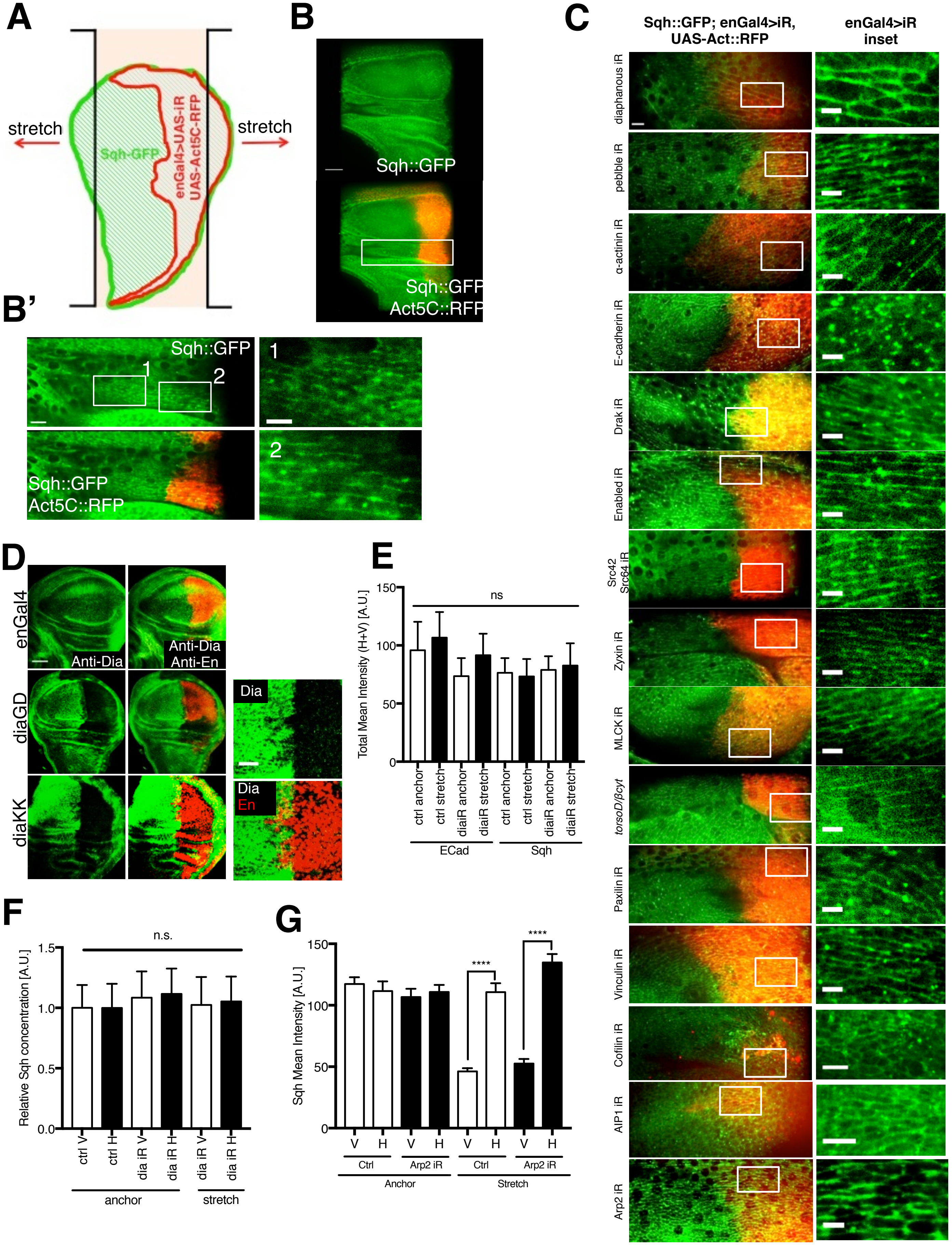

Figure S4, related to Figure 4. Screen identifies MyoII polarity is induced downstream of Diaphanous.

(A) Schematics representing screen rationale: wing disc expressing Sqh::GFP and RNAi of interest using *en*Gal4 driver (*en*Gal4 marked by UAS-Act5C::RFP expression) is subjected to stretch; the readout of the screen is comparison of Sqh polarity on anterior (control) and posterior (RNAi/overexpression) side.

(B) Stretched *Sqh::GFP, enGal4> UAS-Act5C::RFP* wing disc.

(B') Close up of disc shown in (B) showing Sqh:GFP polarity in anterior (inset 1) and posterior (inset 2, marked in red) wing disc compartment.

(C) Screen candidates and observations using system described in A and B. Insets show Sqh::GFP in posterior compartment, scale bars, 5μm.

(D) Diaphanous antibody staining (green) in discs expressing *en*Gal4> UAS-*dia*-RNAi (GD VDRC library, labeled as diaGD) and *en*Gal4>*dia*-RNAi (KK VDRC library, labeled as diaKK). Discs are stained with DAPI (blue), Phalloidin-647 (white) and anti-Engrailed (red, marks posterior side). D inset shows strength of enGal4 driver is patchy around anterior – posterior boundary: some Engrailed positive cells (red) do not have visible *diaphanous* knock-down (green).

(E) Total myosin levels calculated from experiment in Fig4F in anchor and stretch with wildtype anterior and *dia*-RNAi expression in the posterior of the wing disc; error bars represent S.E.M.

(F) Relative change in junctional Sqh concentration in anchor and stretch wing discs described in (A) that are expressing *dia*-RNAi in the posterior compartment and are wild type in the anterior compartment. Quantification from discs shown in (Fig4F). Values normalized to anchored vertical junction concentration, n=4; error bars represent S.E.M.

(G) Quantification of stretched discs expressing Sqh::GFP and *enGal4>UAS-Arp2 RNAi* and *UAS-Act5c-RFP*, presented in (C); error bars represent S.E.M.

****$p<0.0001$ with *t*-test. Scale bars, 50 μm (D), 30 μm (B), 10 μm (B'), 5 μm (B' and C insets)

Duda et al. Fig S5 related to Figure 5

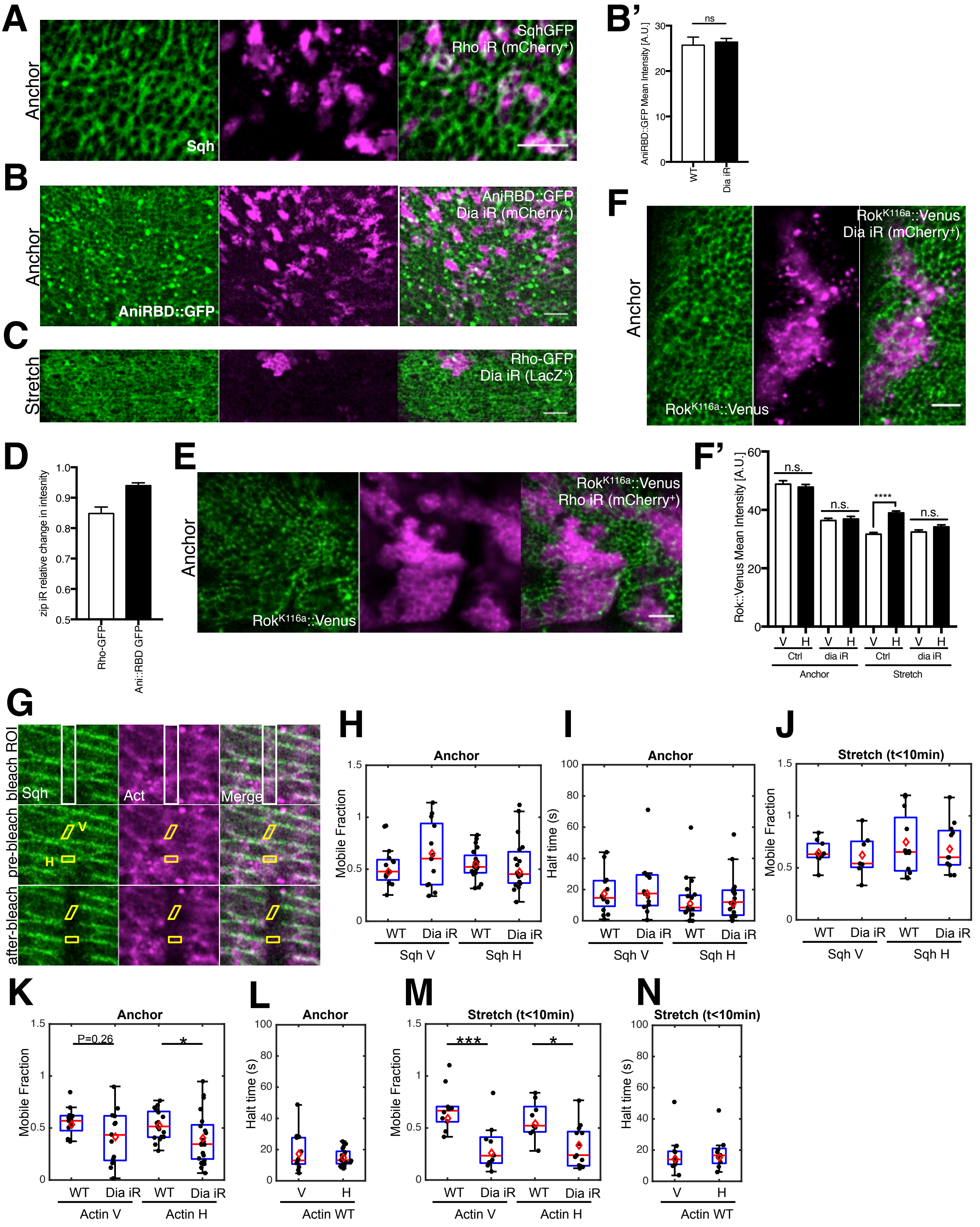

Figure S5, related to Figure 5. Mechanism of Diaphanous in MyoII polarity

(A) *rho*-RNAi clones (indicated in magenta) in anchored Sqh::GFP expressing discs.

(B) *dia*-RNAi clones (indicated in magenta) in anchored AniRBD::GFP expressing discs. (B') quantification of AniRBD::GFP junctional mean intensity in wildtype regions and *dia*-RNAi clones; error bars represent S.E.M.

(C) *dia*-RNAi clones (indicated in magenta) in stretched Rho::GFP protein trap expressing discs. Discs were fixed under stretch and stained, Rho::GFP boosted with anti-GFP-FITC.

(D) Quantification of the relative change in junctional intensity for AniRBD::GFP and Rho::GFP protein trap *zip*-RNAi clones compared to wildtype region; error bars represent S.E.M.

(E) *rho*-RNAi clones (indicated in magenta) in anchored Rok^{K116a}::Venus expressing discs.

(F) *dia*-RNAi clones (indicated in magenta) in anchored Rok^{K116a}::Venus expressing discs. (F') Quantification of Rok^{K116a}::Venus mean junctional intensity for horizontal and vertical junctions in anchored and stretched discs expressing *dia*-RNAi clones, n=3, ****$p<0.0001$ with *t*-test.

(A-F): scale bar 10μm; error bars represent S.E.M.

(G) Fluorescence recovery after photo-bleaching diagram of bleaching and junction analysis. White bounding box shows the bleach region and the yellow boxes mark the horizontal and vertical junctions analyzed for recovery as described in experimental procedures. Wing discs were used as described in (FigS4A) and that co-expressed UAS *dia*-RNAi.

(H-I) FRAP mobile fraction and half time of Sqh::GFP on horizontal and vertical junctions in anchored discs expressing either Sqh::GFP, enGal4> UAS-Act5CRFP or Sqh::GFP, enGal4>UAS-dia RNAi, UAS-Act5CRFP.

(J) FRAP mobile fraction of Sqh::GFP on horizontal and vertical junctions in discs expressing either Sqh::GFP, enGal4> UAS-Act5CRFP or Sqh::GFP, enGal4>UAS-dia RNAi, UAS-Act5CRFP subjected to <10 min stretch.

(K-L) FRAP mobile fraction and half time of Act5C::RFP on horizontal and vertical junctions in anchored discs expressing either Sqh::GFP, enGal4> UAS-Act5CRFP or Sqh::GFP, enGal4>UAS-dia RNAi, UAS-Act5CRFP.

(M-N) FRAP mobile fraction and half time of Act5C::RFP on horizontal and vertical junctions in discs expressing either Sqh::GFP, enGal4> UAS-Act5CRFP or Sqh::GFP, enGal4>UAS-dia RNAi, UAS-Act5CRFP subjected to <10 min stretch.

In all boxplots (H-N), red line indicates the median, red diamond the mean, edges the 25th/75th percentile, and whiskers the most extreme data points that are not considered to be outliers. Black points represent individual junctions. * $p<0.05$, ***$p<0.001$ with Wilcoxon rank-sum test.

Duda et al. Fig.S6 related to Figure 6

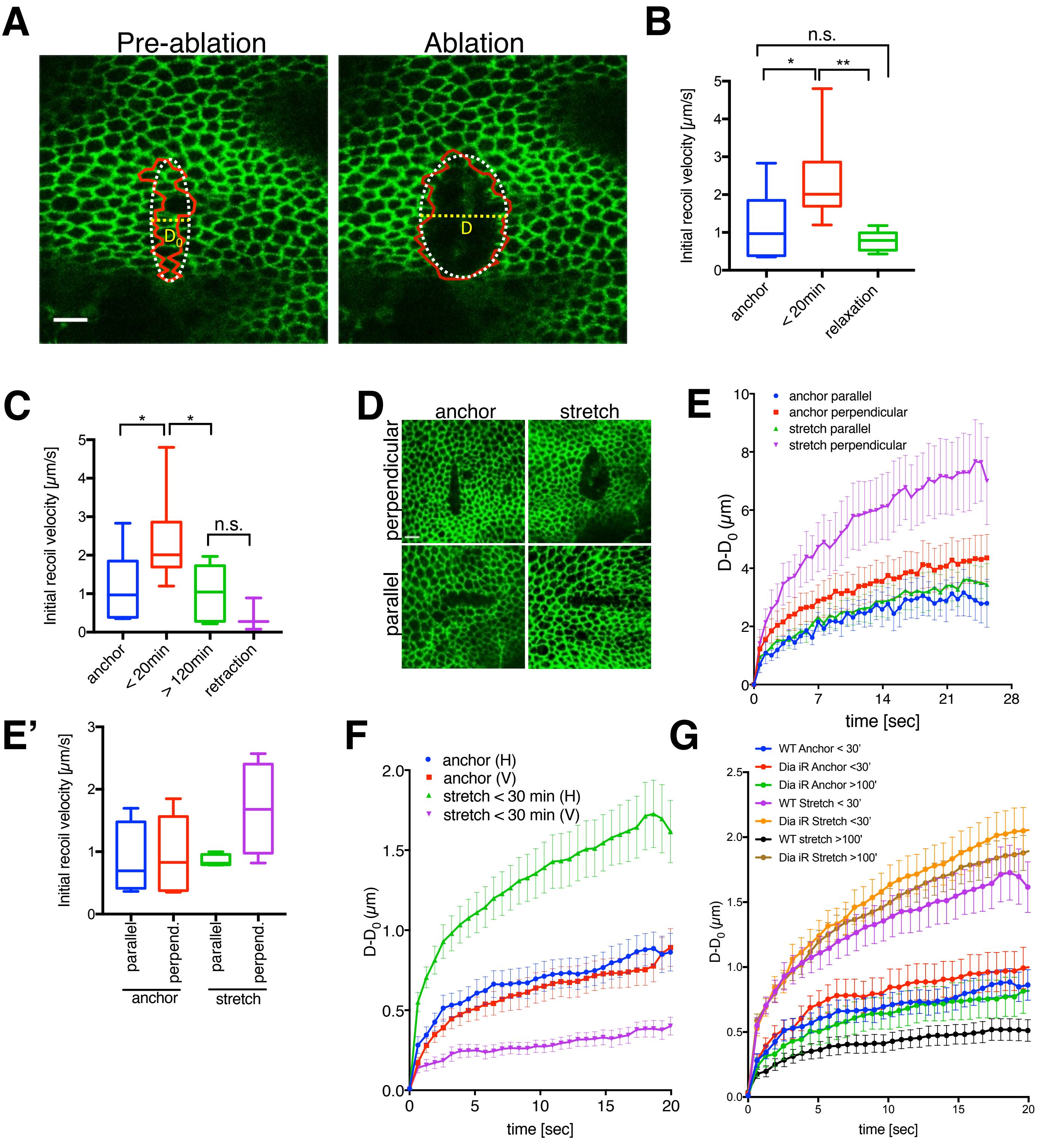

Figure S6, related to Figure 6. Cell- and tissue-scale tension measurements in stretched tissue.

(A) Stretched tissue prior to (left panel) and after (right panel) ablation of circa 10 cells in the direction perpendicular to stretch. Red line outlines ablated area; white dotted line represents ellipse fitted to ablation area; yellow dotted line indicates short ellipse axis measuring ablation recoil.

(B, C) Box plots of initial recoil velocity quantified as a short ellipse length change ($D-D_0$) over time of 1.27 seconds post-ablation (first timepoint was omitted due to sporadic unspecific effects from extensive injury) as described in (A). Median is represented by horizontal line; 75th and 25th percentiles are represented by top and bottom of the boxes respectively. Plot in (B) represents experiment where discs were anchored, stretched (<20 min) and relaxed; plot in (C) shows the same data for anchored and stretched (<20 min) discs as in (B) but plotted together with discs stretched >120 min and retracted (compressed to initial shape) from Fig6A'. N=3-11 wing discs per condition.

(D) Laser cuts (perpendicular or parallel to the line of stretch) across circa 10 cells in E-cad::GFP expressing wing disc tissue subjected to anchoring or short stretching (< 30 min).

(E) Plot showing anchored and stretched (<20 min) discs ablated in the direction perpendicular and parallel to stretch as described in (D); data is plotted as a mean ± S.E.M. (E') Box plot of initial recoil velocity from (E) calculated as described for (B) and (C). N=3-11 wing discs per condition.

(F) Plot showing increase in distance between recoiling vertices ($D-D_0$) over time [sec.] following laser cuts of single horizontal (H) and vertical (V) junctions in anchored and stretched E-cad::GFP expressing discs. Data is plotted as a mean ± S.E.M, n=17-23 cuts per condition.

(G) Plot showing increase in distance between recoiling vertices ($D-D_0$) over time [sec.] following laser cuts of single horizontal (H) junctions in anchored, short term and long term stretched E-cad::GFP expressing discs, with and without *dia*-RNAi expression through rotundGal4 driver. Data is plotted as a mean ± S.E.M, n=8-30 cuts per condition and refers to initial recoil velocity presented in Fig6C.

* p<0.05, **p<0.01 with *t*-test. Scale bars, 10 μm.

Duda et al. Fig.S7 related to Fig.6 and Fig. 7

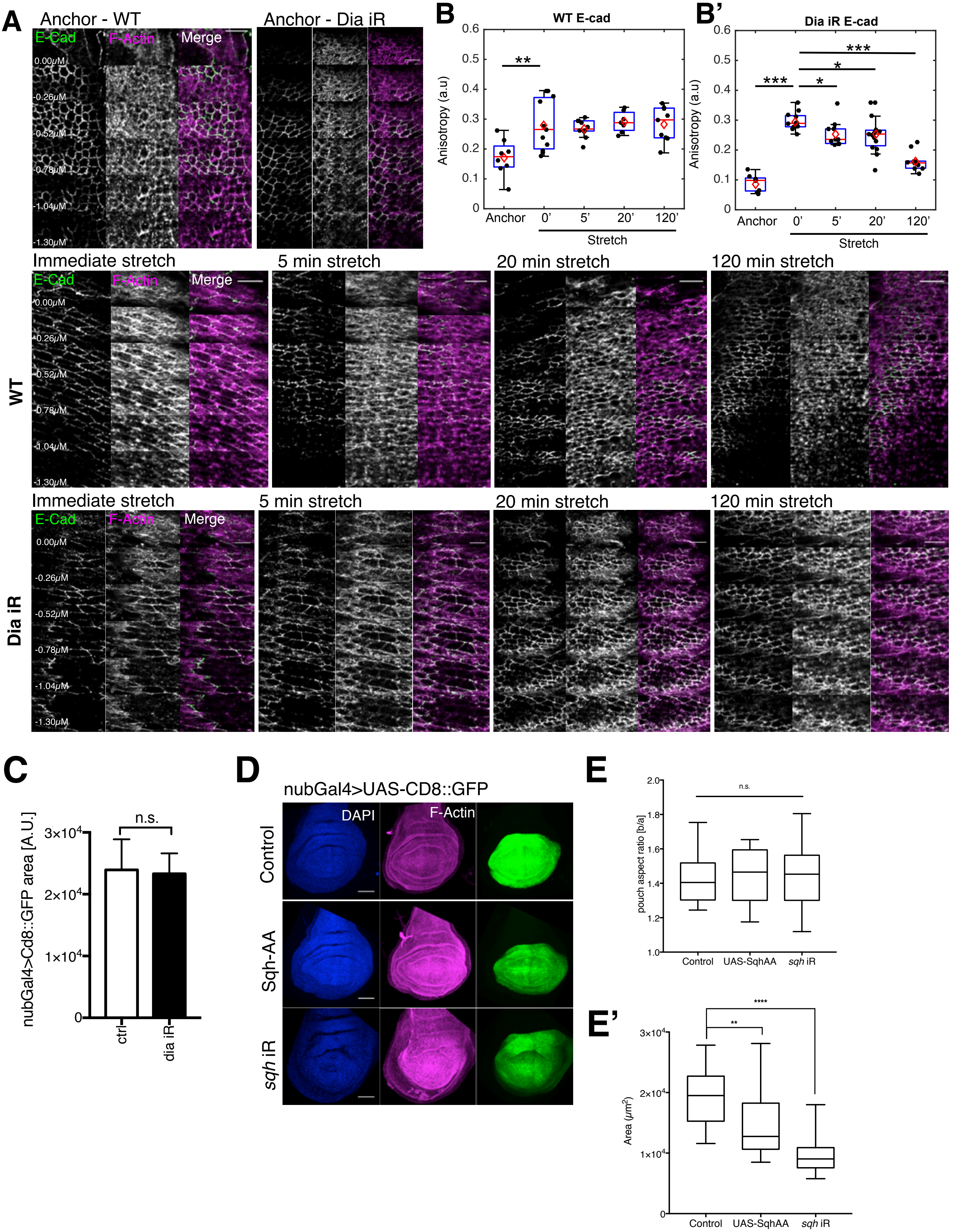

Figure S7, related to Figure 6, 7. Actin remodelling dynamics and disc shape analysis

(A) Wildtype discs expressing Ecad::6XRFP or Ecad::GFP;rnGal4>UAS-*dia*-RNAi were fixed whilst in anchored or stretched positions, then stained for filamentous actin with Phalloidin. Imaging was deconvolved and is presented in 0.26μm z steps to show junctional actin and apical meshwork (5μm scale bars).

(B) Anisotropy quantification as described in experimental procedures for Ecad in wildtype and *dia*-RNAi expressing (B') wing discs in (A). N=3-5. In all boxplots, red line indicates the median, red diamond the mean, edges the 25th/75th percentile, and whiskers the most extreme data points that are not considered to be outliers. Black points represent individual ROIs as described in experimental procedures. *p<0.05, **p<0.01 and ***p<0.001 with Wilcoxon rank-sum test.

(C) Mean wing pouch area quantified as total *nub*Gal4>Cd8::GFP (GFP positive) area in control condition (UAS-Cd8::GFP) and diaphanous knock-down (*dia* iR + UAS-Cd8::GFP) showing no apparent size difference. Error bars indicate standard deviation.

(D) Discs expressing *nub*Gal4 and Cd8::GFP (control) and UAS-*SqhAA* or *Sqh*-iR labeled with DAPI; ellipse is fitted into the pouch region and aspect ratio determined as long ellipse axis (b) divided by short ellipse axis (a) as described for Fig7G.

(E) Box plot showing distribution of wing disc pouch aspect ratios in conditions described in D.

(E') Box plot showing distribution of mean wing pouch area quantified as total *nub*Gal4>Cd8::GFP (GFP positive) area in control condition (UAS-Cd8::GFP) and myosin perturbation (RNAi + UAS-Cd8::GFP), described in (D,E). Median is represented by horizontal line; 75th and 25th percentiles are represented by top and bottom of the boxes respectively. *p<0.05, **p<0.01 ***p<0.002, ****p<0.0001, with *t*-test. Scale bars, 50μm (D) 5μm (A)
