# Supplementary material for: Polarization of Myosin II Refines Tissue Material Properties to Buffer Mechanical Stress
Source: Dev Cell. 2019 Jan 28;48(2):245–260.e7. doi: 10.1016/j.devcel.2018.12.020 (PMC6353629; doi:10.1016/j.devcel.2018.12.020)
Supplement: Document S2. Article plus Supplemental Information [file mmc8.pdf]

Article

Developmental Cell

Polarization of Myosin II Refines Tissue Material Properties to Buffer Mechanical Stress

Graphical Abstract

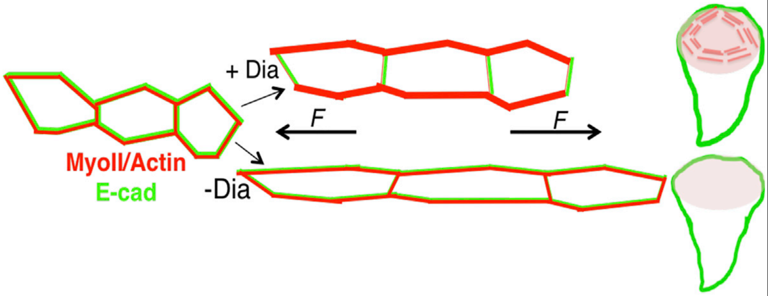

Authors

Maria Duda, Natalie J. Kirkland, Nargess Khalilgharibi, ..., Guillaume Charras, Buzz Baum, Yanlan Mao

Correspondence

y.mao@ucl.ac.uk

In Brief

Duda and Kirkland et al. develop a tissue-stretching device and demonstrate that Myosin II polarizes with mechanical stretch, a rapid reaction that changes tissue stiffness and elasticity to protect it from physical damage and maintain tissue shape. MyoII polarization is mediated downstream of actin polymerization via Diaphanous.

Highlights

- MyoII forms polarized cables upon mechanical stretch
- MyoII polarity increases tissue stiffness and elasticity in the direction of stretch
- MyoII cables protect tissues from fluctuations in forces to maintain tissue shape
- MyoII polarization is Rok-independent but mediated by actin polymerization via Dia

Duda et al., 2019, Developmental Cell *48*, 245–260
January 28, 2019 © 2018 The Authors. Published by Elsevier Inc.
https://doi.org/10.1016/j.devcel.2018.12.020

CellPress

Developmental Cell
Article

CellPress

Polarization of Myosin II Refines Tissue Material Properties to Buffer Mechanical Stress

Maria Duda,[1,8] Natalie J. Kirkland,[1,8] Nargess Khalilgharibi,[1,2,3] Melda Tozluoglu,[1] Alice C. Yuen,[1] Nicolas Carpi,[4] Anna Bove,[2,6] Matthieu Piel,[4] Guillaume Charras,[2,5,6] Buzz Baum,[1,5] and Yanlan Mao[1,5,7,9,*]
[1]MRC Laboratory for Molecular Cell Biology, University College London, London WC1E 6BT, UK
[2]London Centre for Nanotechnology, University College London, London WC1E 6BT, UK
[3]Centre for Computation, Mathematics and Physics in the Life Sciences and Experimental Biology (CoMPLEX), University College London, London WC1E 6BT, UK
[4]Institut Curie, PSL Research University, CNRS, UMR 144, Paris 75005, France
[5]Institute for the Physics of Living Systems, University College London, London WC1E 6BT, UK
[6]Cell and Developmental Biology, University College London, Gower Street, London WC1E 6BT, UK
[7]College of Information and Control, Nanjing University of Information Science and Technology, Nanjing, Jiangsu 210044, China
[8]These authors contributed equally
[9]Lead Contact
*Correspondence: y.mao@ucl.ac.uk
https://doi.org/10.1016/j.devcel.2018.12.020

SUMMARY

As tissues develop, they are subjected to a variety of mechanical forces. Some of these forces are instrumental in the development of tissues, while others can result in tissue damage. Despite our extensive understanding of force-guided morphogenesis, we have only a limited understanding of how tissues prevent further morphogenesis once the shape is determined after development. Here, through the development of a tissue-stretching device, we uncover a mechanosensitive pathway that regulates tissue responses to mechanical stress through the polarization of actomyosin across the tissue. We show that stretch induces the formation of linear multicellular actomyosin cables, which depend on Diaphanous for their nucleation. These stiffen the epithelium, limiting further changes in shape, and prevent fractures from propagating across the tissue. Overall, this mechanism of force-induced changes in tissue mechanical properties provides a general model of force buffering that serves to preserve the shape of tissues under conditions of mechanical stress.

INTRODUCTION

Tissue shape and function are tightly coupled: simple changes in cell geometry affect fundamental processes such as cell growth, death, or the direction of cell divisions (Chen et al., 1997; Théry et al., 2007). During development, force-guided tissue shape changes are both critical and instructive for morphogenesis (Heisenberg and Bellaïche, 2013; Legoff et al., 2013; Munjal et al., 2015; Vuong-Brender et al., 2017). In developed and differentiated tissues, such as those of the adult heart or lung, it is important to preserve the correct shape for normal organ function (Liu et al., 1999; Lyon et al., 2015). This is especially challenging in face of the continuous fluctuations in mechanical forces experienced by the tissues. Mechanical forces can originate from tissue intrinsic processes such as cell division, cell death, and cell shape change or from the external environment through processes such as extracellular matrix (ECM) remodeling, mechanical changes in neighbouring tissues, gravity, fluid flow, or air flow (Etournay et al., 2015; Haigo and Bilder, 2011; Jufri et al., 2015; Legoff et al., 2013; Liu et al., 1999; Mao et al., 2013; Monier et al., 2015; Porazinski et al., 2015). Unremitted mechanical stress can impact tissue integrity and fidelity of cell division and cause tissue fracture (Casares et al., 2015; Harris et al., 2012; Lancaster et al., 2013). Although tissues have developed a plethora of active cellular-scale mechanisms to dissipate mechanical stresses, such as cell extrusions, divisions, transitions, and fusions, the full impact of these active cellular behaviors can take up to several hours (Campinho et al., 2013; Etournay et al., 2015; Heisenberg and Bellaïche, 2013; Marinari et al., 2012; Wyatt et al., 2016; Wyatt et al., 2015). Over short timescales, their response depends on their mechanical properties at rest, such as elasticity or stiffness (Brückner and Janshoff, 2015; Skoglund et al., 2008; Zhou et al., 2009), and on their ability to dissipate applied stress (Khalilgharibi et al., 2016). However, it is not known if cells also possess mechanisms that allow them to rapidly adapt in ways that preserve cell and tissue form when stress is sustained for long periods. Here, we utilize the *Drosophila* wing imaginal disc to investigate the molecular and cellular basis of epithelial mechanics and the role of dynamic remodeling in tissue shape maintenance and injury responses in stretch-challenged tissues.

RESULTS

MyoII is Essential for Setting Tissue Stiffness and Elasticity

Cell shape is defined by the balance of forces exerted on cells through the external environment (such as cell-cell and cell-ECM

This is an open access article under the CC BY license (http://creativecommons.org/licenses/by/4.0/).

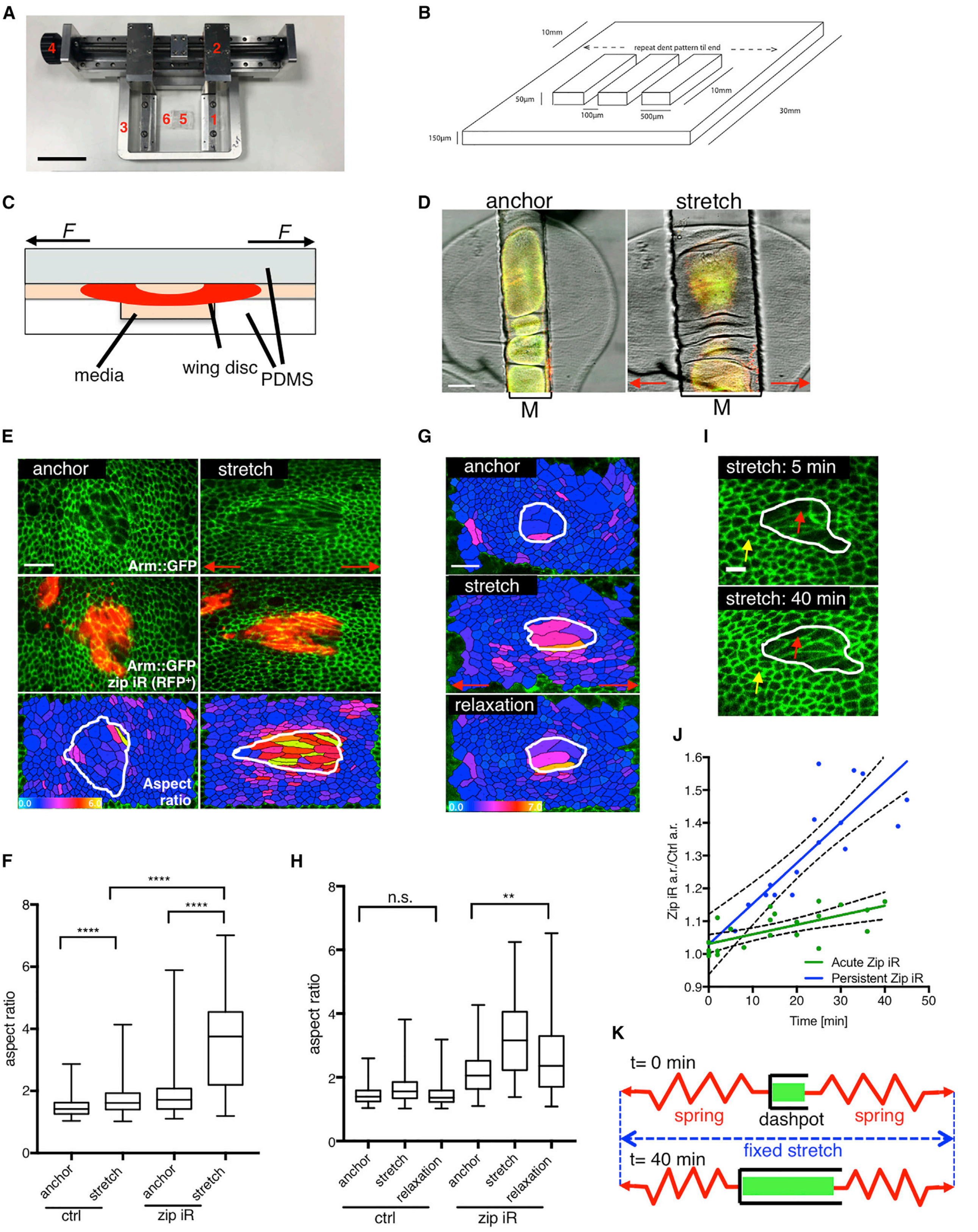

(legend on next page)

adhesion) and the forces exerted by intracellular cell components such as the actomyosin cortex (Mao and Baum, 2015). Therefore, the pathways controlling cell shape are likely to be critical in responses to mechanical stress. We focused on the non-muscle Myosin II (MyoII) contractility pathway, as MyoII is recruited to the cell cortex in force-driven morphogenetic processes such as mesoderm invagination in gastrulation as well as by deformation applied through micropipette aspiration (Fernandez-Gonzalez et al., 2009; Pouille et al., 2009). MyoII anisotropy has also been correlated with emergent tension patterns in the *Drosophila* wing epithelium (Legoff et al., 2013; Mao et al., 2013; Singh et al., 2018). Although studies of these processes suggested that MyoII could be sensitive to mechanical stimuli, it is unclear whether MyoII accumulation is the cause or consequence of tissue tension. To test this directly, we looked at the function of MyoII in responding to a mechanical challenge. In order to directly apply a controllable and quantifiable mechanical stress to a tissue, we designed a tissue-stretching and compression device (Figures 1A–1D). Contrary to previous setups that rely on the adhesion of cells to polydimethylsiloxane (PDMS) (Aragona et al., 2013; Aw et al., 2016; Eisenhoffer et al., 2012), this device uses a unique mechanism to clamp tissue explants to stretch or compress stiff tissues, while suspended in growth media (see Figure 1C; STAR Methods). The wing disc is positioned over the microchannel and while the sides of the wing disc are clamped between the two PDMS layers, the central portion of the tissue is effectively suspended in the microchannel, free of contact with PDMS. This central portion is perfused with *ex vivo* culture media (Mao et al., 2013, 2011). Stretching of the PDMS sandwich concomitantly stretches the suspended central region inside the microchannel, and this is the region we image in all experiments (marked M in Figure 1D). Such a setup eliminates the non-specific effects of interactions between the tissue and PDMS, such as external shear forces, which could not be excluded in previously published devices. We have verified that discs are viable under anchored or stretched conditions for up to 3.5 h, as cell divisions are maintained throughout this period (data not shown).

To determine if the deformations we applied resulted in forces of a physiological magnitude, we experimentally derived the elastic index of the tissue (Young modulus) (Figures S1A–S1E; Video S1; see also STAR Methods). We subsequently calculated that the forces applied during stretching of the disc are in the range of 5–30 nN per cell area and that about 30 nN is required to produce 100% strain. This force is of a similar magnitude to the cellular forces previously measured *in vivo* (Stewart et al., 2011).

To test the function of MyoII in mechanical responses, we generated Myosin heavy chain (*zipper*) RNAi (*zip*-RNAi) clones in tissues expressing β-catenin (Armadillo) fused to GFP (Arm::GFP) (Figure 1E). Upon application of a 20-min exogenous unidirectional stretch, we quantified cell shape changes in Arm::GFP-expressing tissue containing clones with *zip*-RNAi mutant cells, which have reduced levels of the regulatory light chain Sqh (Figure S1K). We used automatically extracted changes in cell aspect ratios as a measure of cell deformation (Figures 1E and S1F). While both the control and *zip*-RNAi tissue increased their aspect ratio significantly, as expected for a tissue under stretch, the *zip*-RNAi cells elongated 2-fold more than the corresponding control cells (Figures 1E and 1F). These results imply that cells depleted of MyoII are more deformable than control cells, consistent with AFM measurements on isolated cells (Martens and Radmacher, 2008). Furthermore, 10 min following tissue relaxation, control cells returned to their pre-stretch shape while *zip*-RNAi cells still remained significantly deformed, implying reduced contractility (Figures 1G and 1H). Similar results were obtained in tissues depleted of the MyoII regulatory light chain via the expression of dsRNA targeting *spaghetti squash* (*sqh*-RNAi) (Figures S1I and S1J).

To gain further insight into the physical properties of clones depleted of MyoII, we applied a defined static stretch over an extended period of time (40 min). Following the initial

Figure 1. Myosin II RNAi Clones are Softer and Less Elastic

(A) Stretching and compression device; 1: clamping mechanism, 2: arms, 3: stage insert, 4: drive mechanism, 5: media-filled PDMS chamber, 6: two layers of stretchable elastomer (PDMS), one of which is pre-patterned with microchannels.

(B) Scheme of PDMS pre-patterning; the dimensions of the microchannels are 80–120 μm in width and 50 μm in depth.

(C) Cross-sectional schematic view of the stretching device. The wing disc (in red) is positioned over the microchannel with sides clamped by the two PDMS layers. The central portion of the tissue is submerged in the microchannel and perfused with *ex vivo* culture media (Mao et al., 2013, 2011).

(D) Stretching of the PDMS sandwich concomitantly stretches the wing disc inside the microchannel (top-down view); wing disc resting on a stretching device (anchor) and 10 min after stretch (stretch); M = microchannel.

(E) *zipper* RNAi clones (*zip* iR, in red) in Arm::GFP expressing anchored and stretched third instar wing disc; lower panel shows color-coded cell aspect ratio in anchored and stretched discs. (Bright colors indicate high elongation.) The *zip*-RNAi clone is outlined in white.

(F) Box plot showing distribution of cell aspect ratio for control (wild-type) and *zip*-RNAi cells in anchored and stretched (20 min) wing disc; median represented by horizontal line, 75th and 25th percentiles are represented by top and bottom of the boxes respectively; n = 8 wing discs.

(G) Color-coded aspect ratio in control and *zip*-RNAi cells (*zip*-RNAi clone outlined in white) subjected to anchoring, stretching (20 min), and relaxation (10 min).

(H) Box plot showing distribution of cell aspect ratio in anchored, stretched (20 min), and relaxed (10 min) wing disc for control (wild-type) and *zip*-RNAi cells; median represented by horizontal line; 75th and 25th percentiles are represented by top and bottom of the boxes respectively; n = 3 wing discs.

(I) *zip*-RNAi clone in Arm::GFP wing disc at the beginning (5 min) and at the end (40 min) of stretching experiment. Yellow and red arrows indicate the control and *zip*-RNAi cells respectively.

(J) Change of *zip*-RNAi aspect ratio (a.r.) relative to change in control cells aspect ratio (a.r.) in the course of stretch, blue line shows fitted regression for persistent *zip*-RNAi clones (n = 3); green line shows fitted regression for silent clone system with 16 h *zip*-RNAi expression prior to dissection (n = 6); dotted lines represent 95% confidence bounds of the best fit line.

(K) Schematics describing the behavior of *zip*-RNAi cells; spring represents control cells which contract and pull on less elastic *zip*-RNAi clone (dashpot) during 40 min stretch. Red horizontal arrows indicate direction of stretch.

**$p < 0.01$, ****$p < 0.0001$ with t test. n.s. = non-significant. Scale bars, 5 cm (A), 50 μm (D), 10 μm (E and G), and 5 μm (I).

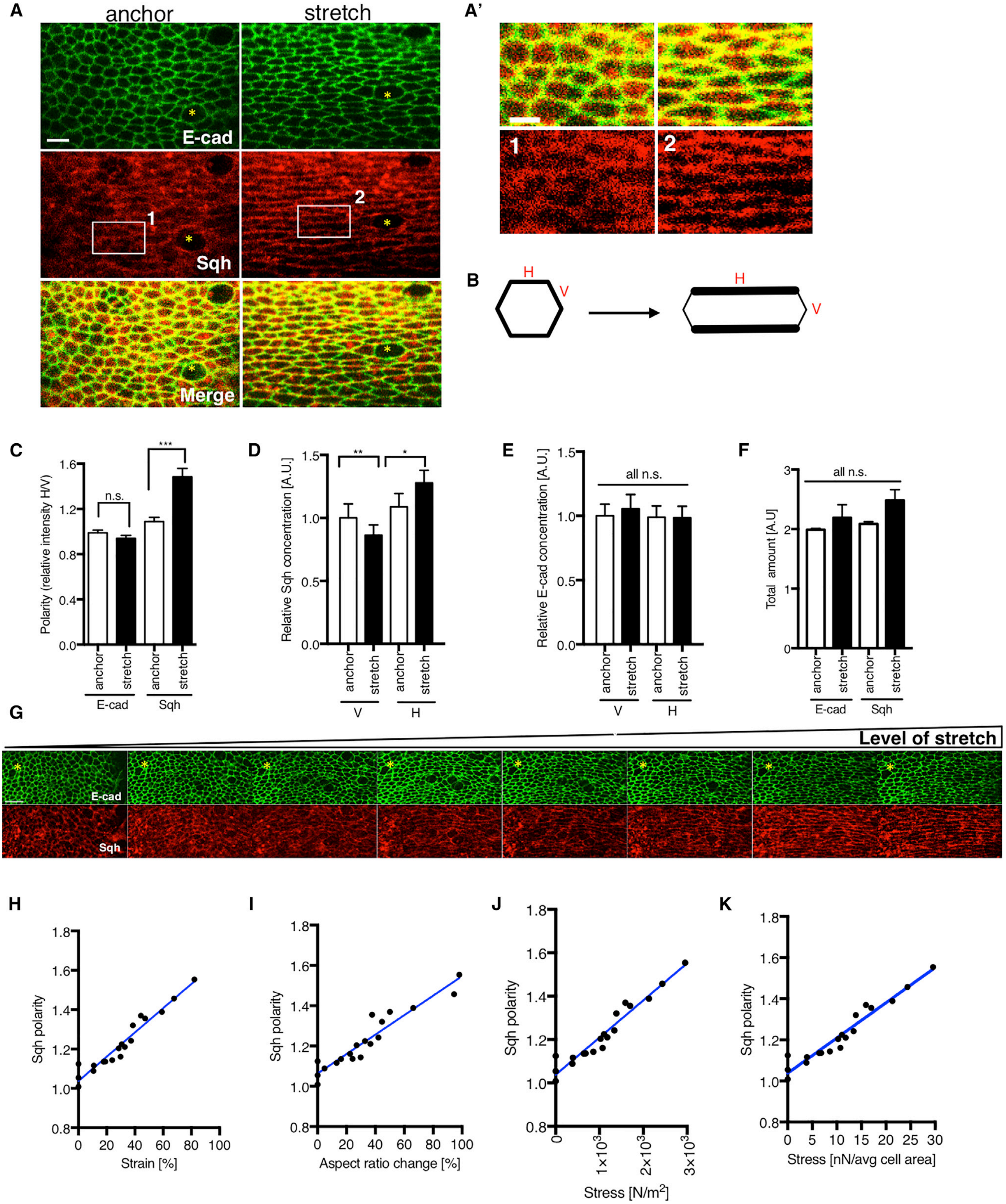

Figure 2. Myosin II Polarizes with Mechanical Stretch

(A) Wing disc expressing E-cad::GFP and Sqh::mCherry prior to stretch (anchor) and after stretch (15 min). (A′) Insets of discs shown in (A).

(B) Schematics demonstrating change in Sqh concentration before and after stretch on vertical (V) and horizontal (H) junctions.

(legend continued on next page)

deformation, *zip*-RNAi cells continued to deform along the line of stretch with time (Figures 1I and 1J; Video S2). In fact, as *zip*-RNAi cells elongated, control cells on either side of a *zip*-RNAi clone contracted over time (10 min compared to 40 min stretch), returning to their pre-stretch shape, as measured by their aspect ratio in the direction of the stretch (Figure S1G). As a further control, to avoid the potential side effects of persistent *zip*-RNAi, which can affect cell size, we also removed *zip* acutely using a ‘‘silent clone’’ technique, whereby the *zip*-RNAi clones are allowed to grow but do not begin the knockdown until a temperature shift activates the RNAi (STAR Methods). Again, cells with acutely reduced MyoII activity showed increased deformability compared to neighboring control cells, albeit more moderately, due to the milder knockdown in acute clones (Figures 1J and S1H).

This behavior is reminiscent of the behavior of two springs (control cells) in series connected via a dashpot (*zip*-RNAi clone) (Figure 1K). Thus, the gradual expansion of the *zip*-RNAi population is likely to be a direct consequence of the control cells contracting when pulled, against the less contractile *zip*-RNAi cells whose viscous properties now dominate. Taken together, these findings suggest that MyoII is essential for setting both the stiffness and elasticity of the wing disc epithelial tissue.

Total and Activated MyoII Polarize with Mechanical Stretching

To gain mechanistic insight into how MyoII might govern tissue stiffness, we looked at the localization of MyoII regulatory light chain (Sqh), Sqh::mCherry, co-expressed with a marker of cell junctions E-cadherin::GFP (E-cad) in anchored (non-stretched, just clamped in the device) and stretched discs. Strikingly, within a few minutes of stretch, MyoII became strongly enriched on the junctions parallel to the axis of stretch, while no change of localization was detected for E-cad (Figures 2A, 2A′, S2A, and S2A′). This occurred irrespective of different genetic backgrounds, fluorescent tags, or stretch orientation (Figures S2B–S2C′). Quantification of mean junctional intensities of MyoII and E-cad (explained in Figures S2D–S2F; STAR Methods) in anchored and stretched wing discs demonstrated that there was a significant enrichment of MyoII, but not E-cad on stretched (horizontal) junctions relative to non-stretched (vertical) junctions, a property hereafter referred to as MyoII polarity (Figures 2A and 2C). Furthermore, the relative enrichment of MyoII resulted from both MyoII recruitment to stretched junctions and concomitant loss from non-stretched junctions, while total junctional MyoII amount remained unchanged (Figures 2D–2F). This suggests that stretch redistributes MyoII from non-stretched to stretched junctions (Figure 2B).

To understand how the magnitude and duration of mechanical stretch affect MyoII polarity, we compared wing discs stretched for short (up to 20 min) and long (up to 3 h) periods and varied the magnitude of stretch. Over short timescales, the extent of MyoII polarization was proportional to cell deformation, stress, and strain (Figures 2G–2K; Video S3). When a stretched tissue with clear MyoII polarity was subjected to laser ablation to locally remove tension, MyoII polarity was rapidly lost (Figures 3E and 3F). Similarly, both cell shape and polarity induced by stretch were fully reversible (Figures 3A and 3B).

Interestingly, over the course of a prolonged (up to 3 h) stretch, we observed a gradual decrease in MyoII polarity, even though the extent of cell deformation was maintained. Notably, by the end of the assay, when stretch was alleviated and MyoII polarity was completely lost, cells were 25% more elongated than they were in their pre-stretched state (Figures 3C and 3D).

These findings demonstrate that MyoII polarity follows the pattern of cell and tissue deformation over short timescales. However, during a prolonged stretch, MyoII polarity is reduced, allowing the tissue to acquire a new, preferred homeostatic shape.

MyoII Polarity is Rho-Kinase Independent

Previous work has shown that assembly of contractile MyoII filaments is regulated by direct phosphorylation of myosin light chain by Rho-Kinase (Rok), downstream of the Rho1 pathway (Riento and Ridley, 2003). To uncover the molecular mechanism driving stretch-induced MyoII polarity, we therefore examined Rok function in control wing discs and in wing discs subjected to mechanical forces. While *Rok*-RNAi reduces levels of MyoII phosphorylation in normal wing discs (Figures 3G and 3I), in line with previous work, the loss of Rok (*Drok*^{2} loss of function clones, *enGal4>Rok RNAi* and treatment with Rok inhibitor Y27632) did not prevent MyoII polarization (Figures 4A–4D, S3A–S3H; Video S6).

MyoII Polarization Requires the Formin Diaphanous

To identify the upstream regulator of stress-induced MyoII polarity, we performed an RNAi screen, targeting a selection of potential or reported MyoII regulators (Figure S4A; STAR Methods). While Sqh::GFP was expressed ubiquitously, the *engrailed-Gal4* (*en-Gal4*)-driver-targeted dsRNAs to the posterior side, marked by expression of *UAS-Actin5C::RFP* (Act5C::RFP), which we confirmed had no visible effect on MyoII polarity (Figures S4B and S4B′). A positive result was defined as a failure to polarize Sqh::GFP upon stretch, while retaining junctional Sqh (Figure S4A; see STAR Methods for details). Unexpectedly, using this assay, none of the reported Myosin regulators such as Rok

(C) Quantification of mean fluorescent intensity of E-cad::GFP and Sqh::mCherry on horizontal (H) junctions relative to the intensity on vertical (V) junctions (referred to as ‘‘polarity’’) prior to and after 15 min stretch. The detailed rationale of intensity determination is described in Figure S2 and in STAR Methods.

(D and E) Quantification of Sqh::mCherry (D) and E-cad::GFP (E) concentration (i.e., mean intensity per junctional unit area) prior and after stretch on both horizontal and vertical junctions normalized to starting concentration on non-stretched (anchored) vertical junctions.

(F) Quantification of total amount of Sqh::mCherry and E-cad::GFP fluorescence on vertical and horizontal junctions in anchored and stretched discs.

(G) Tissue expressing E-cad::GFP and Sqh::mCherry subjected to increasing levels of stretch.

(H) Quantification of Sqh::mCherry polarity (as in C) of experiment described in (G). The level of stretch is measured as a change in tissue strain (deformation relative to unstretched tissue).

(I–K) Sqh::mCherry polarity emergence as a function of cell aspect ratio change (I), stress (J), and stress applied on average cell area of 10 μm^2 (K).

Yellow asterisks indicate equivalent regions in the tissue. N = 8 wing discs (A–F). N = 3 wing discs (G–K). All experiments are plotted as mean ± S.E.M.; * $p < 0.05$, ** $p < 0.01$, *** $p < 0.001$ with t test. Scale bars, 5 μm (A), 3 μm (A′), and 10 μm (G).

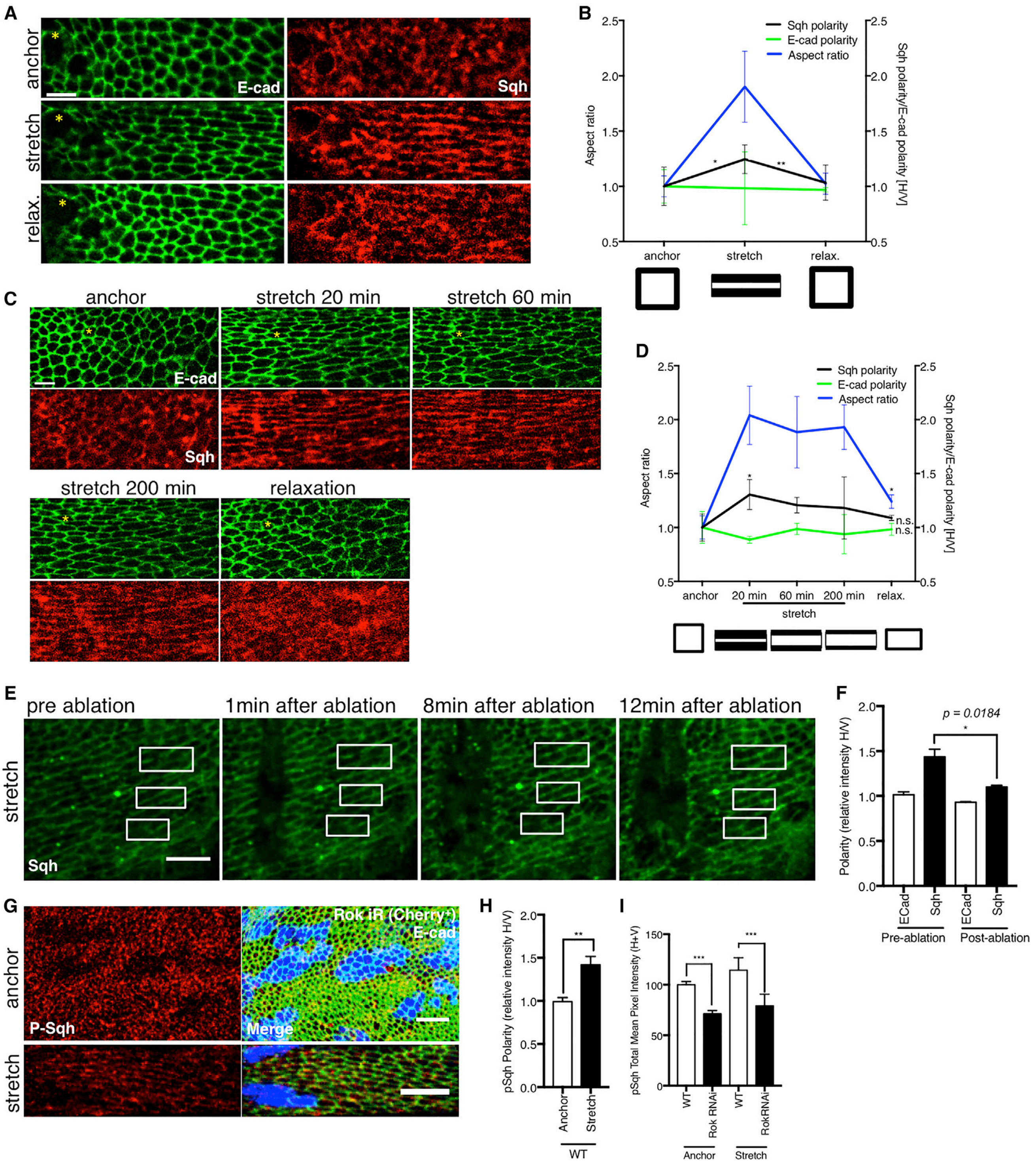

Figure 3. Myosin II Polarization after Mechanical Stretch is Highly Reversible but Changes with Prolonged Stretch and is Phosphorylated

(A) Short (15 min) stretch and relaxation (10 min) of E-cad::GFP and Sqh::mCherry expressing discs.

(B) Quantification of Sqh::mCherry polarity, E-cad::GFP polarity, and aspect ratio change in experiment described in (A); schematics below the graph illustrate quantified changes in cell aspect ratio and Sqh polarity. Statistical test compares Sqh polarity between anchored and stretched or stretched and relaxed disc.

(C) Long (200 min) stretch and relaxation (10 min) of E-cad::GFP and Sqh::mCherry expressing discs.

(D) Quantification of Sqh::mCherry polarity, E-cad::GFP polarity, and aspect ratio change in experiment described in (C); schematics below the graph illustrate quantified changes in aspect ratio and Sqh polarity. Statistical test compares changes with respect to initial (anchored) state. Yellow asterisks indicate equivalent regions in the tissue. N = 3 wing discs.

(legend continued on next page)

or myosin light chain kinase (MLCK) appeared to be required for stretch-induced MyoII polarity (Figure S4C).

Nevertheless, we observed a profound change in polarization when we depleted several actin regulators. Most significantly, these included the formin Diaphanous (Dia), an actin nucleator that generates linear arrays of F-actin that serve as scaffolds for MyoII (two different RNAi lines, Figures 4F–4H and S4D–S4F) (Jégou et al., 2013). The Dia-RNAi polarity defect was further quantified in discs co-expressing E-cad::GFP (Figures 4G and 4H). The depletion of Pebble and Rok, which act together with Dia in many instances and have cytokinetic and cell-size defects similar to those seen in Dia-deficiency, did not produce any MyoII polarity defects (Figures 4B, 4D, S3A, S3B, S3G, S3H, and S4C). Importantly, the upstream regulator Rho1 did affect MyoII polarity (Figures 5A and 5A′).

At the same time, RNAi-induced depletion of the F-actin depolymerization factors cofilin and AIP1 also impaired MyoII polarization (Figures S3I, S3J, and S4C), suggesting that existing F-actin filaments must disassemble for force-induced MyoII polarization (Chen et al., 2015; Nadkarni and Brieher, 2014). Finally, perturbation of branched F-actin, which is believed to be a less favorable scaffold for myosin contractility, by depletion of Arp2, had no effect on MyoII polarity (Figure S4C). Taken together, our data strongly suggest that the dynamic remodeling of linear actin cables generated by formins, downstream of Rho1, is required for MyoII polarization upon stretch. This is in agreement with previous *in vitro* studies that demonstrated that Diaphanous is tension sensitive whereas Arp2 and Arp3 are not (Jégou et al., 2013; Lomakin et al., 2015).

Rho1-Dependent Dia Activity Stabilizes MyoII on Stretched Junctions

To explore this new mechanosensitive pathway in more detail and to understand the polarization of these regulators under conditions of stretch, we investigated the localization of Rho1 and Dia in response to mechanical stretch. Using a ubi-Dia-GFP reporter line, we did not observe Dia localization change upon stretch (Figure 4I). In contrast, Rho1, a known regulator of Dia was found to polarize upon stretch (Figures 4J and 4J′) consistent with it being able to respond to mechanical perturbations (Lessey et al., 2012).

Interestingly, Rho1 depletion caused reduction in Dia (Figures 5B and 5B′) and in Rok (Figures 5D and 5D′). By contrast, Rho1 was not significantly affected by *dia*-RNAi (Figures S5B, S5B′, and S5C), but Rok polarity was (Figures 5E and 5E′), likely due to loss of MyoII polarity as seen with *dia*-RNAi (Figure 4F), which affects Rok polarization (Figures 4A, 4E, and 4E′), see summary diagram Figure 5F. Similarly, MyoII depletion using *zip*-RNAi also compromised Rho1-GFP levels and reduced its polarization upon stretch (Figures 5C, 5C′, and S5D), implying the presence of feedback MyoII and Rho1 in the system, as previously reported (Munjal et al., 2015; Priya et al., 2015). Therefore, in summary, our results suggest that Dia mechanosensitivity to polarize MyoII is dependent upon Rho1 activity.

However, how does Dia induce MyoII polarity to emerge within minutes of stretch? We reasoned that as Dia is a formin, it is likely to affect actomyosin dynamics. In order to investigate the remodeling dynamics of Actin and MyoII, we performed fluorescence recovery after photobleaching (FRAP) analysis for Actin and MyoII on anchored and stretched wing discs (Figures S5G–S5N; STAR Methods). We found little change in Actin dynamics (Figures S5K–S5N), presumably due to the limitations of the experimental procedure, where the time delays between stretching the tissue and performing FRAP prevent the instant changes in actin to be captured (see Figure 6D). However, we found that during the first 10 min of stretch, MyoII (Sqh) binding is more stable on stretched (horizontal) junctions and that this stabilization is dependent on Dia activity (Figure 5G). This is consistent with findings that tensed actin filaments can stabilize more adenosine diphosphate (ADP)-bound Myosin (Llinas et al., 2015). Presumably the ability to form new actin filaments in response to tissue stretch and tension changes requires Dia in the wing disc.

Tension Increases Immediately after Stretch but Drops Gradually due to Diaphanous-Dependent Actin Remodeling

To directly measure how tension actually changes in the stretched tissue, we used laser ablation to analyze both cell-level and tissue-scale forces. Using long (spanning ~10 cells) and single junctional cuts perpendicular to the stretch, we determined that along the direction of tissue deformation, tissue-scale and cell-scale tension increases significantly directly after stretch and decreases to the pre-stretch (anchor) levels 2 h later (Figures 6A–6C and S6A–S6D; Videos S4 and S5). Further compression of the tissue is required to return cells to their original pre-stretch shape, as evidenced by a further drop in tension (Figures 6A, 6A′, and S6C). In contrast, tissue relaxation following short (<20 min) stretch returns the tissue to the pre-stretched (anchored) tension state (Figure S6B). Changing the orientation of the laser cuts to the direction orthogonal to stretch revealed that the tension was isotropic in the anchored non-stretched tissue. However, these junctions had lower tension in the stretched tissue, indicating that the majority of tissue tension was borne by junctions aligned with the direction of stretch (Figures S6D–S6E′). The pattern of junctional recoil was consistent with the changes in

(E) Stretched Sqh::GFP expressing discs with large tissue ablation for acute, local tension release. Subsequent loss of myosin polarization shown at 1, 8, and 12 min after ablation. White boxes highlight re-emergence of Sqh::GFP signal on vertical junctions.
(F) Quantification of Sqh::GFP polarity and ECad::mCherry for experiment described in (E). Statistical test compares change in polarity of stretched state to polarity after ablation once recoil had stopped (post-ablation). N = 3 wing discs.
(G) Activated Myosin (anti-phosphorylated-Sqh; in red) and anti-E-cad (green) staining in anchored and stretched discs with *Rok*-RNAi clones (Cherry+, blue).
(H) Quantification of activated Myosin (anti-phosphorylated-Sqh) polarity in anchored and stretched discs; n = 5, p = 0.0022.
(I) Quantification of total mean intensity of activated Myosin (anti-phosphorylated-Sqh) in wild-type regions and *Rok*-RNAi clones for anchored and stretched discs. Statistical tests compare change in mean intensity of wild-type regions to *Rok*-RNAi for anchored and stretched state separately; n = 4, p = 0.0002.
Error bars indicate S.E.M.; * $p < 0.05$, ** $p < 0.01$, and *** $p < 0.001$ with t test (F, H, and I). Scale bars, 10μm (A and C), 10 μm (E and G).

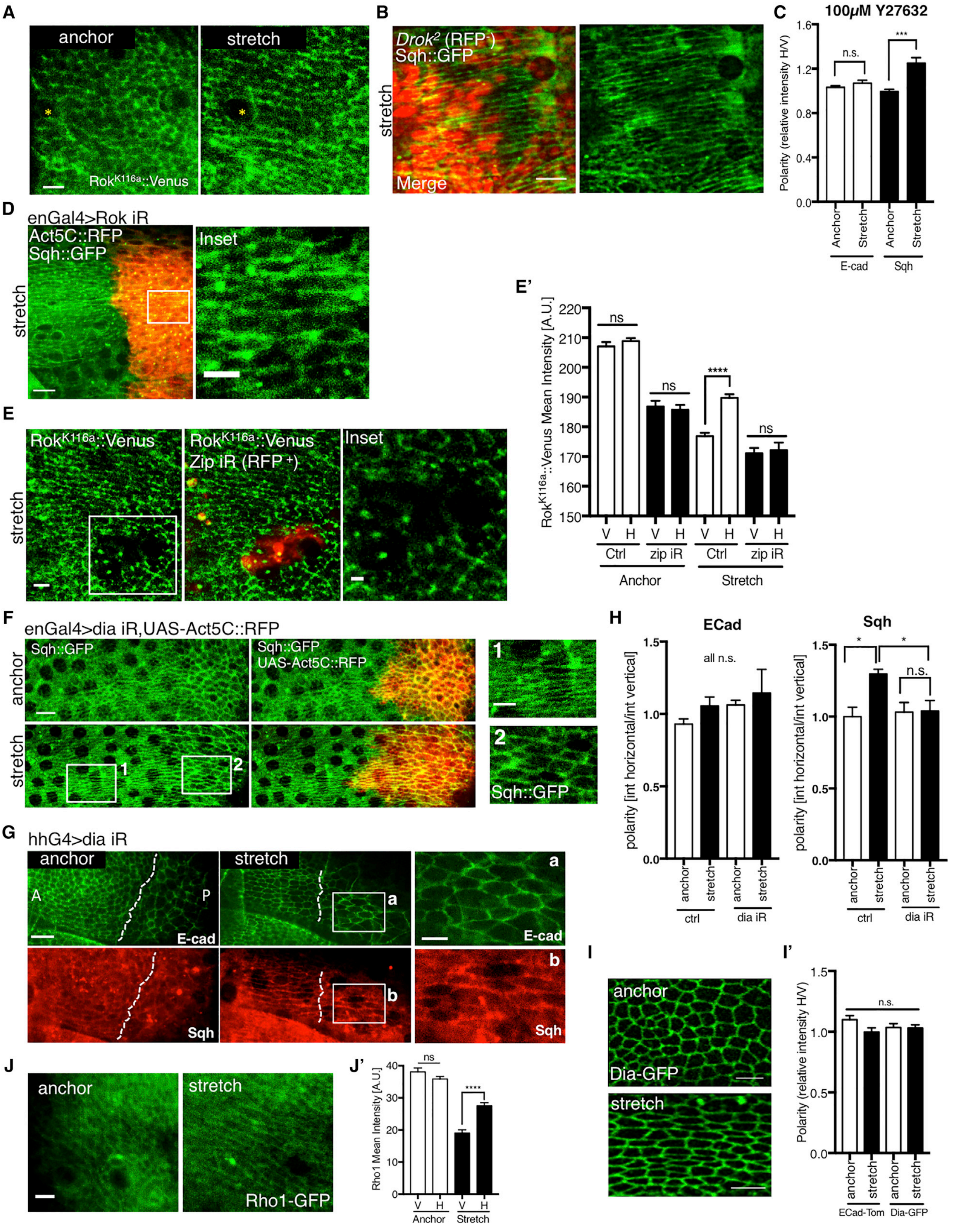

Figure 4. Myosin II Polarity is Rok Independent and Depends on Dia Expression

(A) Rok^{K116a}::Venus localization in anchored and stretched third instar imaginal discs.

(B) *Drok*^{2} mitotic clones (RFP^{-}) in Sqh::GFP third instar imaginal disc.

(legend continued on next page)

MyoII accumulation (compare Figure 6B′ with Figures 3B–3D). Taken together, we can conclude that MyoII polarity correlates well with tension changes in the tissue in response to stretch, rather than cell shape changes.

Strikingly, however, under *dia*-RNAi conditions, the tension did not drop after 2 h (Figures 6C and S6G). The observed drop of tension in prolonged stretch is therefore likely a consequence of actomyosin remodeling, which is dependent on Dia activity. To investigate how Dia affects F-actin remodeling after stretch, we performed high-resolution F-actin imaging in wild-type (WT) and *dia*-RNAi discs.

Immediately upon stretch, F-actin polarizes (i.e., there is an enrichment on the horizontal stretched junctions) and becomes very anisotropic when compared to the anchored state (Figures 6D and S7A; STAR Methods). This F-actin organization is then maintained for up to 20 min after stretch but returns to the anchored-like (non-stretched) state after 120 min of stretch (Figures 6D (WT) and 6D′), even though the cells are still deformed, as evidenced by E-cadherin images and anisotropy analysis (Figures S7A (WT) and S7B). In *dia*-RNAi wing discs, junctional F-actin does not polarize after stretch (vertical junctions still clearly visible in Figure 6D Dia iR panel) and the ability of F-actin to remodel and return to a new steady-state configuration after prolonged stretch is also lost (Figures 6D (Dia iR) and 6D″). Taken together these results suggest that Dia is required immediately after stretch to polarize junctional actin structures to provide a substrate for polarized MyoII binding, and this is what underlies myosin polarization within 5 min of stretch. Dia is also responsible for actin remodeling after prolonged stretch to allow the system to evolve to a new resting tensile state.

Stretch-Induced MyoII Cables Rigidify the Tissue

Junctional laser ablations not only reveal the tensile state of tissues but also lead to micro-injuries in the cells. We wondered whether we could observe any differences in tissue responses to injury in anchored and stretched tissue (see STAR Methods). Interestingly, we noticed two types of behaviors depending on the proximity to the injury site. Locally, at the level of injured cells (row 0), we noticed significantly higher tissue recoil in stretched cells, consistent with the measured increase in tissue tension (Figures 7A–7C and S6F). Remarkably, in cell rows further away from the injury site (row 2 and row 3) this relationship became reversed, with anchored cells showing higher degrees of recoil compared to stretched tissue (∼100% more recoil in rows 2 and 3) (Figure 7C). This is despite a much higher global tension in the latter condition (Figure S6). Thus, it appears that tensile stress stiffens the tissue to buffer mechanical perturbations such that they fail to propagate beyond the site of injury. Given that MyoII polarization contributes to the stiffness of the tissue (Figures 1E and 1F), it is likely that this stiff tissue lattice is controled by stretch-induced tissue scale MyoII polarization. To directly test this, we performed the same wound propagation analysis in *dia*-RNAi stretched discs, where MyoII does not polarize across the tissue. The wound recoil now propagates further beyond the wound site (Figures 7D and 7E). Together, our data strongly suggest that the presence of polarized MyoII cables stiffens the tissue to preserve tissue morphology and to prevent local tissue damage from propagating. In summary, stretch-induced tension increase depends on MyoII contractility, not polarity per se, but stretch-induced stiffening requires MyoII polarity, not contractility in general.

MyoII Polarity Pathway is Essential for Maintaining Tissue Shape

Having identified a condition that specifically decreases MyoII polarity, we next tested its impact on cell deformation upon mechanical stretch. In agreement with our MyoII RNAi data (Figures 1E–1F), Dia depletion significantly increased cell deformation (Figure 7F). Taken together, our results showed that Dia is required for induction of MyoII polarity and subsequent cell shape maintenance in tissues challenged with extrinsic mechanical stress.

How does this pathway influence morphogenesis during normal development? To test the requirements of Dia-dependent MyoII polarization in the context of mechanical forces emerging *in vivo*, we explored previous reports showing that mechanical tension emerges in proximal wing disc cells as a result of proliferation anisotropy (Legoff et al., 2013; Mao et al., 2013). Interestingly, the increased tension was shown to correlate with the polarization of phosphorylated MyoII in these cells (Legoff et al., 2013; Mao et al., 2013). However, whether MyoII polarized as a direct response to mechanical force was never shown. Our data showing that MyoII polarizes as a direct response to exogenous stretch suggests that the same mechanism is occurring during *in vivo* wing disc development. To test whether this is the case, we targeted *dia*-RNAi expression to the posterior compartment of the disc. In agreement with exogenous stretching data, we observed defective Sqh::GFP

(C) Quantification of polarization for ECad::GFP and Sqh::Cherry expressing discs treated with Rok inhibitor Y27632 prior to stretching experiment. Statistical tests compare the change in polarity between the anchor and stretch states, n = 5.

(D) *Sqh::GFP, enGal4>UAS-Rok-RNAi,* and *UAS-Act5CRFP* discs subjected to stretching; inset demonstrates Sqh::GFP polarity in *Rok*-RNAi cells (marked by red).

(E) *zip*-RNAi clones (labeled as zip iR, indicated in red) in stretched third instar Rok^{K116a}::Venus discs. Quantifications of Rok^{K116a}::Venus mean intensity in (E′).

(F) Discs expressing Sqh::GFP and *enGal4>UAS-dia-RNAi, UAS-Act5c-RFP* that were stretched; UAS-Act5C::RFP marks *dia* iR (posterior side) in red. Insets compare Sqh::GFP polarity in stretched *enGal4>dia iR* and *UAS-Act5c-RFP* discs; panel 1 refers to anterior (ctrl) side; panel 2 refers to posterior (*dia* iR) side.

(G) Tissue marked with E-cad::GFP and Sqh::mCherry, expressing *dia*-RNAi (*dia* iR) with hhGal4 driver and subjected to stretching. Dotted line indicates A-P compartment boundary. Inset demonstrates lack of Sqh::GFP polarity in *dia*-RNAi cells.

(H) Quantification of E-cad::GFP polarity and Sqh::mCherry polarity in experiment described in (G). Polarity is measured as mean fluorescent intensity on horizontal junctions relative to vertical junctions (see STAR Methods for details); n = 4 wing discs.

(I) Dia::GFP localization in anchored and stretch third instar imaginal discs. (I′) Quantification of Dia::GFP polarity and ECad::tomato polarity in anchor (n = 3) and stretch (n = 6).

(J) Rho1::GFP protein trap localization in anchored and stretched third instar imaginal discs. (J′) Quantification of Rho1-GFP mean junctional intensity.

Error bars indicate S.E.M.; *p < 0.05, **p < 0.01, ***p < 0.001, and ****p < 0.0001 with t test. Scale bars, 2 μm (D, F, and G: inset), 5 μm (A, B, E, I, and J), 10 μm (B, D, F, and G).

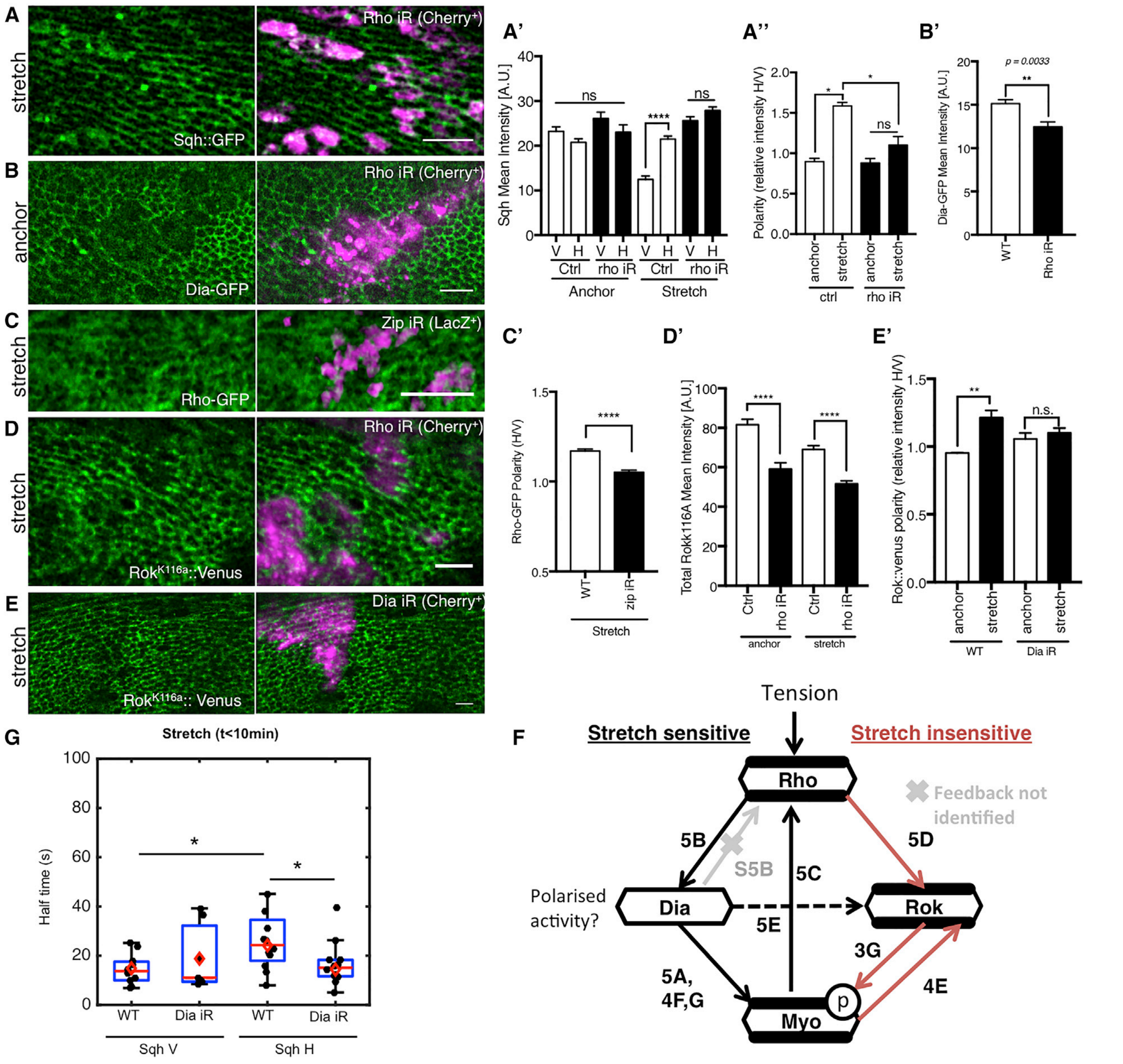

Figure 5. Stretch-Sensitive MyoII Polarization Pathway Induces Stabilization of MyoII on Stretched Junctions

(A) *rho*-RNAi clones (magenta) in stretched Sqh::GFP expressing discs. (A′) Quantification of Sqh intensity on horizontal and vertical junctions in anchor (Figure S5A) and stretch states; n = 2–3. N.B. Absolute intensities are not comparable between anchor and stretch discs as different clones are used for each analysis.

(A″) Quantification of Sqh polarity on anchor and stretch states in control and *rho*-RNAi clones.

(B) *rho*-RNAi clones (indicated in magenta) in anchored Dia::GFP expressing discs. (B′) Quantification of Dia::GFP mean intensity in clone regions compared to wild-type regions in anchored disc; n = 5.

(C) *zip*-RNAi clones (magenta) in stretched Rho::GFP expressing discs. Discs were fixed under stretch and stained. Rho::GFP boosted with anti-GFP-FITC. (C′) Quantification of Rho::GFP polarity in stretched discs expressing zip-RNAi clones and Rho::GFP; n = 4, $p < 0.001$.

(D) *rho*-RNAi clones (magenta) in stretched Rok^{K116a}::Venus expressing discs.

(D′) Quantification of Rok^{K116a}::Venus total mean intensity in *rho*-RNAi clones and in WT control tissue, in anchor (n = 2), and stretched (n = 7) discs.

(E) *dia*-RNAi clones (magenta) in stretched Rok^{K116a}::Venus expressing discs. (E′) Quantification of Rok^{K116a}::Venus polarity in anchored (Figure S5F) and stretched discs expressing *dia*-RNAi clones; n = 3.

(A′–E′) Error bars indicate S.E.M.; * $p < 0.05$, ** $p < 0.01$, and **** $p < 0.0001$ with t test. (A-E) Scale bars, 10 μm.

(F) Pathway schematic summarizing the molecular pathways regulating MyoII polarity. The polarization of MyoII protein levels are stretch sensitive and via Rho and Dia, but it's phosphorylation per se is stretch insensitive and via Rho and Rok.

(G) FRAP half time of Sqh::GFP on horizontal and vertical junctions in discs expressing either Sqh::GFP, enGal4>UAS-Act5CRFP, or Sqh::GFP, enGal4>UAS-*dia*-RNAi, or UAS-Act5CRFP subjected to <10 min stretch. The red line indicates the median, the red diamond the mean, edges the 25th and 75th percentiles, and whiskers the most extreme data points that are not considered to be outliers. Black points represent individual junctions. * $p < 0.05$ with Wilcoxon rank-sum test.

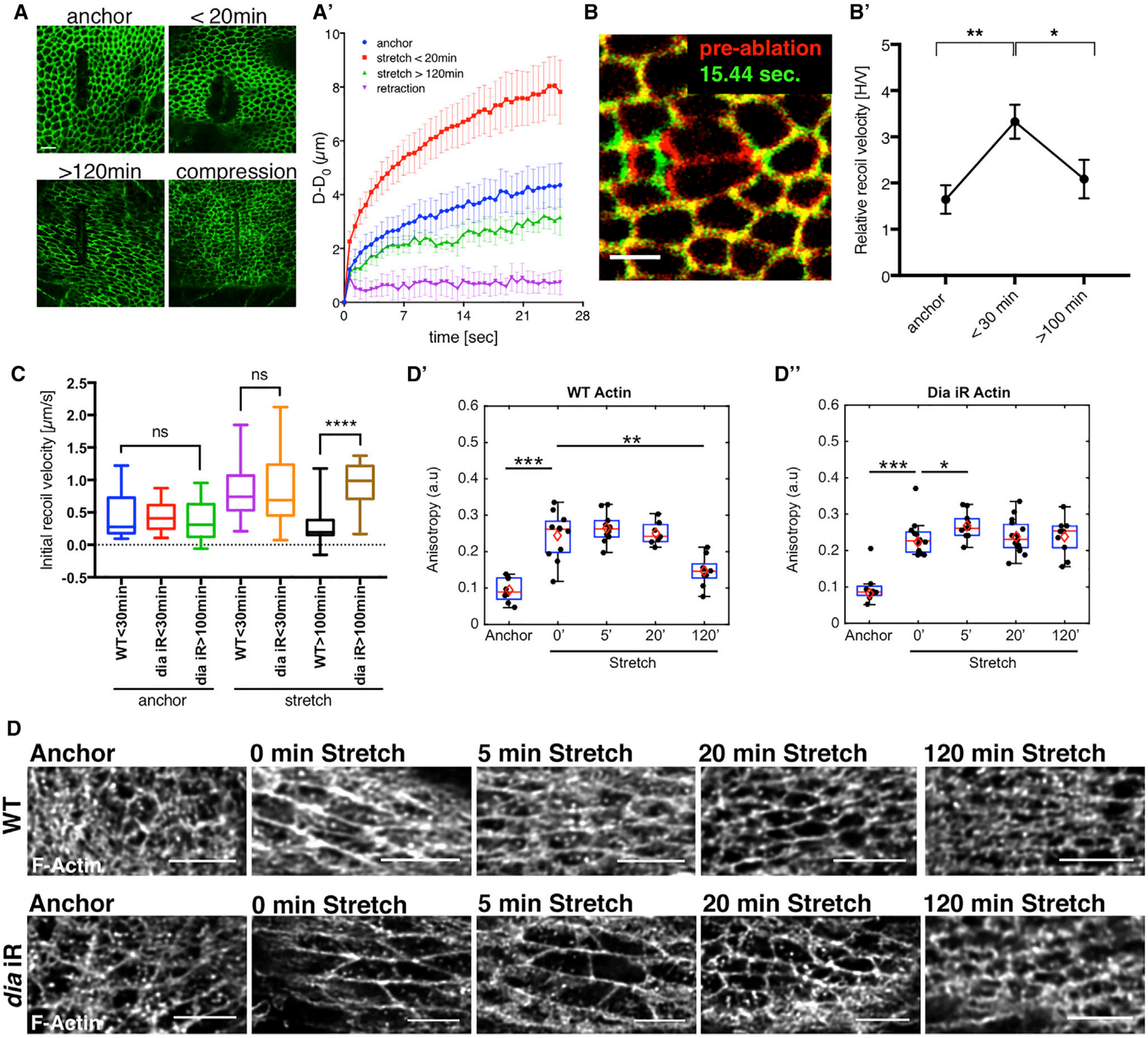

Figure 6. Tissue Tension Correlates with MyoII Polarity and Drops after Prolonged Stretch due to Actin Remodeling

(A) Laser cuts (perpendicular to the line of stretch) across circa 10 cells in E-cad::GFP expressing wing disc tissue subjected to anchoring, short (<20 min) and long (>120 min) stretching and stretch retraction or compression—tissue forced back to the original, pre-stretch shape. (A′) Quantification of the experiment described in (A). Plot shows increase in distance between recoiling short axis (μm) of the fitted ellipse (see Figure S6A for details) against time (s). Data is plotted as mean ± S.E.M.; n = 3–11 wing discs per condition.

(B) An overlay of a junction prior to (red) and 15.44 s after ablation (green). (B′) Plot showing relative initial recoil velocity (velocity on horizontal, stretched junctions normalized to velocity on vertical, non-stretched junctions) for individual junctions ablated in anchored or stretched (<30 min, >100 min) E-cad::GFP expressing discs. Data is plotted as a mean ± S.E.M.; n = 17–24 cuts per condition.

(C) Initial recoil velocity of horizontal, stretched junctions in anchored discs and stretched discs (<30 min and >100 min) expressing ECad::GFP in wild-type and *dia*-RNAi wing discs. Data is plotted as a mean ± S.E.M.; n = 8–30 cuts per condition.

(A–C) * $p < 0.05$, ** $p < 0.01$, *** $p < 0.001$, and **** $p < 0.0001$ with t test.

(D) Wild-type discs expressing Ecad::6XRFP or Ecad::GFP;rnGal4>UAS-*dia*-RNAi were fixed while in anchored or stretched positions, then stained for filamentous actin with Phalloidin, and deconvolved following high-resolution imaging. (D′) Anisotropy quantification for F-Actin in wild-type wing discs. (D″) Anisotropy quantification for F-actin in *dia*-RNAi wing discs. N = 3–5. In all boxplots (D′–D″). The red line indicates the median, the red diamond the mean, edges the 25th and 75th percentiles, and whiskers the most extreme data points that are not considered to be outliers. Black points represent individual ROIs as described in the STAR Methods. * $p < 0.05$, ** $p < 0.01$, and *** $p < 0.001$ with Wilcoxon rank-sum test.

Scale bars, 5 μm (A and D), 3 μm (B).

CellPress

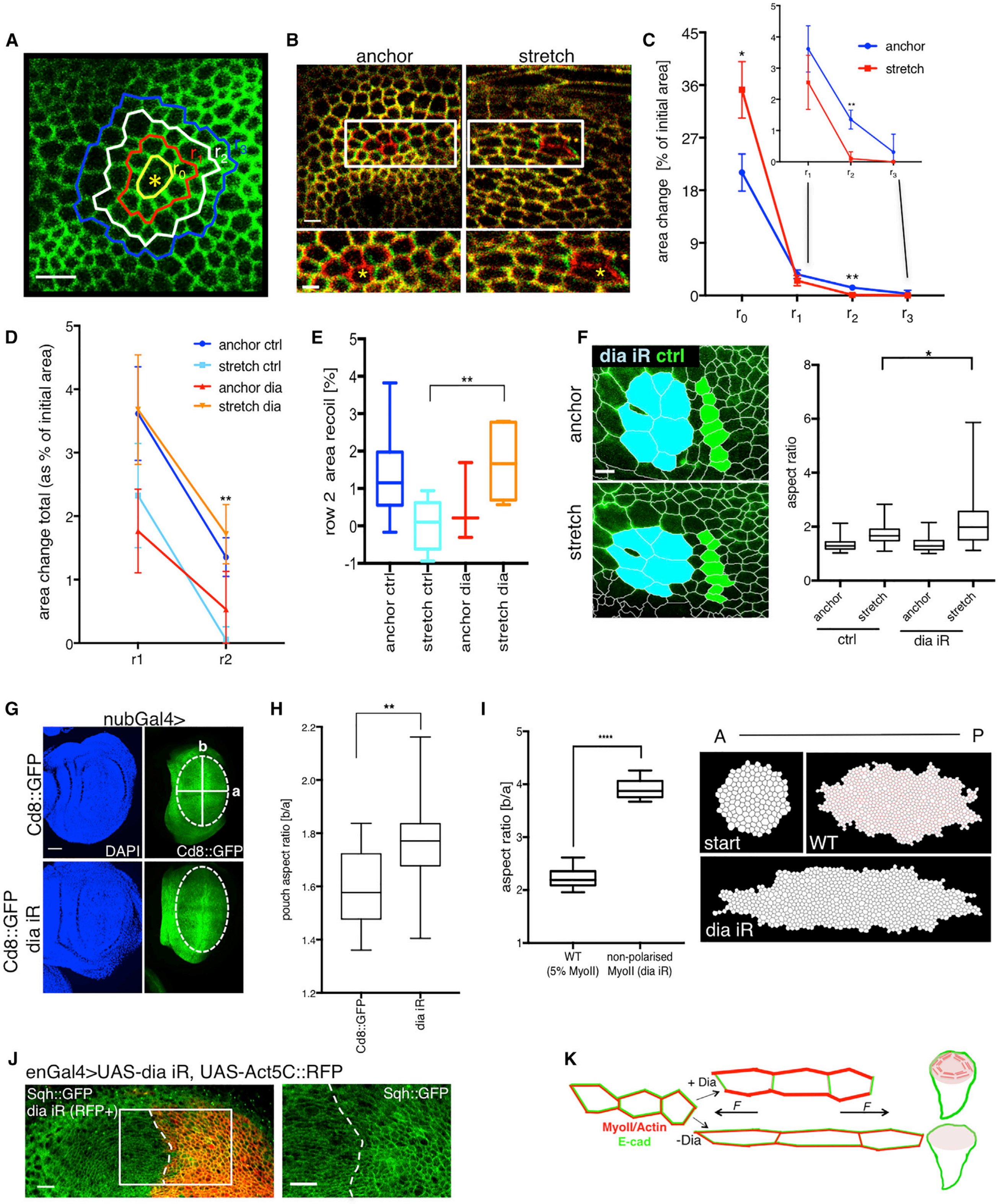

Figure 7. MyoII Polarity Stiffens the Tissue and is Required for Wing Disc Shape Maintenance via Dia-Dependent Actin Polymerization

(A) Image depicting injury site (yellow asterisk) and selection of cell rows used for tissue recoil propagation measurement; r: row.

(B) An overlay of anchored and stretched discs prior to ablation (red) and 15.44 s post ablation (green). Inset zooms in on the area next to ablation site (marked with yellow asterisk).

(legend continued on next page)

polarization of the proximal cells in the posterior compartment when compared to proximal cells in the anterior (control) compartment (Figure 7J) (Legoff et al., 2013; Mao et al., 2013). This result indicated that MyoII polarity was compromised in *dia* depleted cells *in vivo*.

Due to the emergence of mechanical tension in developmental growth, one would predict that the lack of a MyoII polarization mechanism could impact tissue shape. To test this prediction we targeted *dia*-RNAi expression in the whole wing disc pouch with *nubbin-Gal4* driver (nubGal4). Comparing control and *dia*-RNAi discs, we could not observe any significant difference in the overall size (Figure S7C). Strikingly though, *dia*-RNAi wing disc pouches were significantly wider as measured by an increase in their aspect ratio (Figures 7G–7H), whereas overall reduction of tissue tension via MyoII (Sqh) mutations did not significantly affect pouch aspect ratio (Figures S7D and S7E). These results suggest that it is a polarized increase in tissue deformation, rather than an overall increase in tissue deformation, that presumably affects cell shape and long-term cell division orientations in the wing disc.

To explore more fully the likely long-term effects of this experimental perturbation, we performed computational simulations with a vertex model to compare tissue shape in WT wing discs and in Dia-depleted wing discs. The simulations mimic a clone growing along the anterior-posterior (A-P) axis in the pouch, with and without the ability to polarize MyoII along the A-P axis (Figure 7I). The cells then grow and divide according to the cell long axis rule, i.e., to bisect the long axis (Mao et al., 2013; Mao et al., 2011). The clones that cannot polarize MyoII in response to stretch (dia iR) have a significantly higher aspect ratio than WT (Figure 7I). This is because the short-term response of polarizing MyoII affects cell shape, which affects the orientation of divisions (Mao et al., 2011), which accumulates over time to affect the overall tissue shape. These simulations are not aimed at addressing the second role of Dia, which is to remodel the actin to dissipate stress after prolonged stretch. Actin remodeling leads to a gradual reduction of stress, but the cell shapes (that orient divisions) remain unchanged. As such, inclusion of remodeling to the computational model, while maintaining the cell shapes upon stretch, would not alter the cell division patterns and the emerging morphology. In conclusion, our simulation suggests that short-term responses of MyoII polarization can affect the emergent long-term changes in tissue shape, as shown experimentally (Figures 7G–7H).

In summary, the physiological role of Dia-MyoII pathway is to limit tension-induced cell deformation by polarizing actomyosin to stiffen the tissue in the direction of stress (summarized in Figure 7K).

DISCUSSION

Our study has uncovered a MyoII polarity pathway that allows an epithelium to rapidly adapt to a mechanical perturbation. We demonstrated that mechanical stretch induces MyoII polarization, a process that has a dual role in stretch responses by i) maintaining tissue elasticity to main cell shape and ii) setting tissue stiffness to protect against fractures and injuries. Moreover, this happens in a linear way, with more force leading to more polarization.

Both of the identified functions ensure that tissue shape and subsequent function are preserved while mechanical stress is being dissipated. Importantly, the core of the pathway, the formation of polarized MyoII cables, does not follow the canonical rules of MyoII upstream biochemical activation via Rok, seen in processes like *Drosophila* embryonic junctional remodeling, but is downstream of Rho1. Thus, there are two pathways downstream of Rho1, one via Dia that is stretch-sensitive and controls the levels of F-actin and MyoII, and one via Rok that is stretch-insensitive and phosphorylates MyoII (Figure 5F).

Prior *in vitro* studies have demonstrated that application of mechanical tension to isolated actin filaments increases the rate of their polymerization, a phenomenon, dependent in part on the barbed-end associated actin nucleator Diaphanous (Courtemanche et al., 2013; Galkin et al., 2012; Jégou et al., 2013; Kovács et al., 2007; Kozlov and Bershadsky, 2004). Such tensed

(C) Recoil area (% increase from pre-ablation area) for row 0, row 1, row 2, and row 3 in stretched and anchored discs. Data is plotted as mean ± S.E.M.; n = 8–13 discs per condition.

(D) Recoil area (% increase from pre-ablation area) for row 1 and row 2 in stretched and anchored discs for wild-type (control) and *dia*-RNAi discs. Data is plotted as mean ± S.E.M.; n = 8–13 discs per condition.

(E) Box plot showing recoil area (% increase from pre-ablation area) for row 2 (same data as D). Median represented by horizontal line; 75th and 25th percentiles are represented by top and bottom of the boxes respectively. Statistical test compares wild-type (control) stretch with *dia*-RNAi stretch discs.

(F) Discs expressing E-cad::GFP and hhGal4>*dia*-RNAi (KK) subjected to stretch; selection of control (green) and *dia*-RNAi (cyan) for cell deformation analysis. Box plot showing distribution of cell aspect ratio in anchored and stretched (30 min) wing disc for control (E-cad::GFP expressing) and *dia*-RNAi cells; median represented by horizontal line; 75th and 25th percentiles are represented by top and bottom of the boxes respectively; n = 3 wing discs.

(G) Discs expressing *nub*Gal4 and Cd8::GFP (control) or *dia*-RNAi (KK) labeled with DAPI; ellipse is fitted into the pouch region (dotted line) and aspect ratio determined as long ellipse axis (b) divided by short ellipse axis (a) (white lines).

(H) Box plot showing distribution of wing disc pouch aspect ratios in conditions described in (G); n = 19 (ctrl) and 16 (dia iR) wing pouches; horizontal line indicates median.

(I) Influence of myosin increase upon tissue stretching to emergent tissue shape. Start: Initial configuration of the monolayer to be stretched in horizontal direction (A-P axis). WT: Configuration of the monolayer after 12 h simulation with 5% increase in tension levels on horizontal junctions that are within 30 degrees of the stretch axis. The junctions with increased tension are labeled in red. dia iR: Configuration of the monolayer upon 12 h simulation with no myosin response. Quantification of the aspect ratio of the central region of the monolayer (displayed in white). Single cells detached from the clone are excluded from the analysis. Mean and standard deviation of 10 simulation for each case are displayed. No myosin response case is significantly different from 5% myosin increase (two-tailed t test; p value $2.55E^{-11}$).

(J) Endogenous Sqh::GFP polarization in enGal4> *dia* iR, UAS-Act5C::RFP discs. Sqh::GFP does not polarize in *dia*-RNAi side of disc.

(K) Model of diaphanous-myosin force buffering pathway. Tissue (E-cad marks cell junctions in green) subjected to mechanical stretching polarizes MyoII and Actin (in red) in Dia-dependent manner. This mechanism is required to limit tension-induced cell deformation and thus maintain wing disc pouch shape.

* $p < 0.05$,** $p < 0.01$ and **** $p < 0.0001$ with t test unless otherwise stated. Scale bars, 5 μm (B inset and F), 10 μm (A, B and J), 30 μm (G).

actin filaments could also stabilize more ADP-bound Myosin (Llinas et al., 2015). Since Dia is required to polarize Myosin, without itself being polarized by stress, such a mechanism may help polarize MyoII *in vivo*. The extrinsic force applied on the tissue stimulates actin polymerization via the formin Diaphanous in the direction of stretch, downstream of Rho1. These new tensed filaments result in more stable MyoII binding, which could have a scaffolding role for Rok and Rho1. The observed polarization of Rok and Rho1 could serve as an emergent positive feedback signal to further enhance or stabilize polarized actomyosin complexes rather than being the instructive upstream cue.

Gradual changes in mechanical properties such as tissue stiffness are a normal part of development as exemplified in the differentiation of embryonic stem cells (Butcher et al., 2009). Here, we show how epithelial tissues can rapidly refine their mechanical properties as an adaptive and protective response to the constant fluctuations in physical forces from a tissue's environment. This response buffers mechanical stresses to maintain the balance of intrinsic tissue forces and preserve tissue integrity. This fundamental mechanosensitive pathway could play a broad role in force adaptation in both development and pathological situations such as cancer and tissue repair, where tissue mechanics are affected in an uncontrolled manner.

STAR★METHODS

Detailed methods are provided in the online version of this paper and include the following:

- KEY RESOURCES TABLE
- CONTACT FOR REAGENT AND RESOURCE SHARING
- EXPERIMENTAL MODEL AND SUBJECT DETAILS
  - Drosophila melanogaster
- METHOD DETAILS
  - Fabrication of Silicon Master Mold
  - Preparation of Patterned Polydimethylsiloxane (PDMS) Membranes
  - Stretcher Design
  - Stretching Experiments
  - Immunofluorescence
  - Young's Modulus Determination
  - Live Imaging
  - Fixed Sample Imaging
  - Laser Ablations
  - Screen Design
  - Rok Inhibition
  - Fluorescence Recovery after Photobleaching
  - The Vertex Model
- QUANTIFICATION AND STATISTICAL ANALYSIS
  - Analysis of Junctional Intensities and Cell Aspect Ratio
  - Fluorescence Recovery after Photobleaching Analysis
  - Anisotropy Analysis
  - Wing Disc Pouch Shape Analysis
  - Statistical Analysis

SUPPLEMENTAL INFORMATION

Supplemental Information includes seven figures and six videos and can be found with this article online at https://doi.org/10.1016/j.devcel.2018.12.020.

ACKNOWLEDGMENTS

We are grateful to Nic Tapon, in whose lab the development of the stretching device was initiated, for his support. We thank GREM (Griffon Gravure) for building the first prototype of the stretching/compression device. We thank Duncan Farquharson, Simon Townsend, and Piotr Sienkiewicz from the Mechanical and Electronic Workshops at the University College London for the design and execution of the final version of the stretching/compression device. We thank Davide Heller for help with junctional myosin intensity measurements. We thank John Zhang for his help drawing the photomasks and JDPhoto-Tools for printing. We thank Pedro Gaspar for his kind gift of the ubi-Dia-GFP line. We thank all the authors and Shiladitya Banerjee, Martin Raff, Robert Tetley, Franck Pichaud, Nic Tapon, and David Ish-Horowicz for revision of this manuscript. M.D. is funded by a Marie Skłodowska-Curie Horizon 2020 Individual Fellowship (MRTGS). N.J.K. is funded by a MRC PhD studentship. N.K. is funded by the Rosetrees Trust, the UCL Graduate School, the EPSRC-funded doctoral training program CoMPLEX, and a UCL Overseas Research Scholarship. M.T. is funded by a Sir Henry Wellcome Fellowship (Grant No: 103095). M.P. and N.C. are funded through CNRS and Institut Curie. A.B. is funded through EPSRC. G.C. is supported by a consolidator grant from the European Research Council (MolCellTissMech, agreement 647186). B.B. is supported by CRUK 17343, BBSRC BB/K009001/1, and BBSRC BB/J008532/1 grants. Y.M. is funded by an MRC Fellowship MR/L009056/1, a UCL Excellence Fellowship, and a NSFC International Young Scientist Fellowship 31650110472. This work was also supported by MRC funding to the MRC LMCB University Unit at UCL, award code MC_U12266B.

AUTHOR CONTRIBUTIONS

M.D. and N.J.K. designed the research, performed experiments, analyzed the data, and wrote the article. N.K. designed and performed experiments and analyzed results. M.T. performed the simulations in Figure 7. A.C.Y. helped with immunostaining experiments. N.C. and M.P. helped design the prototype of the stretching/compression device. A.B. prepared silicon wafer molds. G.C. and B.B. provided input into research design and data analysis and helped write the article. Y.M. designed the research, designed the prototype of the stretching/compression device, analyzed the data, and wrote the article.

DECLARATION OF INTERESTS

The authors declare no competing interests.

Received: May 10, 2017
Revised: November 26, 2018
Accepted: December 21, 2018
Published: January 28, 2019

REFERENCES

Afshar, K., Stuart, B., and Wasserman, S.A. (2000). Functional analysis of the Drosophila diaphanous FH protein in early embryonic development. Development *127*, 1887–1897.

Aragona, M., Panciera, T., Manfrin, A., Giulitti, S., Michielin, F., Elvassore, N., Dupont, S., and Piccolo, S. (2013). A mechanical checkpoint controls multicellular growth through YAP/TAZ regulation by actin-processing factors. Cell *154*, 1047–1059.

Aw, W.Y., Heck, B.W., Joyce, B., and Devenport, D. (2016). Transient tissue-scale deformation coordinates alignment of planar cell polarity junctions in the mammalian skin. Curr. Biol. *26*, 2090–2100.

Boudaoud, A., Burian, A., Borowska-Wykręt, D., Uyttewaal, M., Wrzalik, R., Kwiatkowska, D., and Hamant, O. (2014). FibrilTool, an ImageJ plug-in to quantify fibrillar structures in raw microscopy images. Nat. Protoc. *9*, 457–463.

Brückner, B.R., and Janshoff, A. (2015). Elastic properties of epithelial cells probed by atomic force microscopy. Biochim. Biophys. Acta *1853*, 3075–3082.

Butcher, D.T., Alliston, T., and Weaver, V.M. (2009). A tense situation: forcing tumour progression. Nat. Rev. Cancer *9*, 108–122.

Campinho, P., Behrndt, M., Ranft, J., Risler, T., Minc, N., and Heisenberg, C.P. (2013). Tension-oriented cell divisions limit anisotropic tissue tension in epithelial spreading during zebrafish epiboly. Nat. Cell Biol. *15*, 1405–1414.

Casares, L., Vincent, R., Zalvidea, D., Campillo, N., Navajas, D., Arroyo, M., and Trepat, X. (2015). Hydraulic fracture during epithelial stretching. Nat. Mater. *14*, 343–351.

Chen, C.S., Mrksich, M., Huang, S., Whitesides, G.M., and Ingber, D.E. (1997). Geometric control of cell life and death. Science *276*, 1425–1428.

Chen, Q., Courtemanche, N., and Pollard, T.D. (2015). Aip1 promotes actin filament severing by cofilin and regulates constriction of the cytokinetic contractile ring. J. Biol. Chem. *290*, 2289–2300.

Courtemanche, N., Lee, J.Y., Pollard, T.D., and Greene, E.C. (2013). Tension modulates actin filament polymerization mediated by formin and profilin. Proc. Natl. Acad. Sci. USA *110*, 9752–9757.

Eisenhoffer, G.T., Loftus, P.D., Yoshigi, M., Otsuna, H., Chien, C.B., Morcos, P.A., and Rosenblatt, J. (2012). Crowding induces live cell extrusion to maintain homeostatic cell numbers in epithelia. Nature *484*, 546–549.

Escudero, L.M., Bischoff, M., and Freeman, M. (2007). Myosin II regulates complex cellular arrangement and epithelial architecture in Drosophila. Dev. Cell *13*, 717–729.

Etournay, R., Popović, M., Merkel, M., Nandi, A., Blasse, C., Aigouy, B., Brandl, H., Myers, G., Salbreux, G., Jülicher, F., et al. (2015). Interplay of cell dynamics and epithelial tension during morphogenesis of the Drosophila pupal wing. Elife *4*, e07090.

Farhadifar, R., Röper, J.C., Aigouy, B., Eaton, S., and Jülicher, F. (2007). The influence of cell mechanics, cell-cell interactions, and proliferation on epithelial packing. Curr. Biol. *17*, 2095–2104.

Fernandez-Gonzalez, R., Simoes, Sde M., Röper, J.C., Eaton, S., and Zallen, J.A. (2009). Myosin II dynamics are regulated by tension in intercalating cells. Dev. Cell *17*, 736–743.

Galkin, V.E., Orlova, A., and Egelman, E.H. (2012). Actin filaments as tension sensors. Curr. Biol. *22*, R96–R101.

Haigo, S.L., and Bilder, D. (2011). Global tissue revolutions in a morphogenetic movement controlling elongation. Science *331*, 1071–1074.

Harris, A.R., Peter, L., Bellis, J., Baum, B., Kabla, A.J., and Charras, G.T. (2012). Characterizing the mechanics of cultured cell monolayers. Proc. Natl. Acad. Sci. USA *109*, 16449–16454.

Heisenberg, C.P., and Bellaïche, Y. (2013). Forces in tissue morphogenesis and patterning. Cell *153*, 948–962.

Heller, D., Hoppe, A., Restrepo, S., Gatti, L., Tournier, A.L., Tapon, N., Basler, K., and Mao, Y. (2016). EpiTools: an open-source image analysis toolkit for quantifying epithelial growth dynamics. Dev. Cell *36*, 103–116.

Huang, J., Zhou, W., Dong, W., Watson, A.M., and Hong, Y. (2009). From the Cover: directed, efficient, and versatile modifications of the Drosophila genome by genomic engineering. Proc. Natl. Acad. Sci. USA *106*, 8284–8289.

Jégou, A., Carlier, M.F., and Romet-Lemonne, G. (2013). Formin mDia1 senses and generates mechanical forces on actin filaments. Nat. Commun. *4*, 1883.

Jordan, P., and Karess, R. (1997). Myosin light chain-activating phosphorylation sites are required for oogenesis in Drosophila. J. Cell Biol. *139*, 1805–1819.

Jufri, N.F., Mohamedali, A., Avolio, A., and Baker, M.S. (2015). Mechanical stretch: physiological and pathological implications for human vascular endothelial cells. Vasc. Cell *7*, 8.

Khalilgharibi, N., Fouchard, J., Recho, P., Charras, G., and Kabla, A. (2016). The dynamic mechanical properties of cellularised aggregates. Curr. Opin. Cell Biol. *42*, 113–120.

Kovács, M., Thirumurugan, K., Knight, P.J., and Sellers, J.R. (2007). Load-dependent mechanism of nonmuscle myosin 2. Proc. Natl. Acad. Sci. USA *104*, 9994–9999.

Kozlov, M.M., and Bershadsky, A.D. (2004). Processive capping by formin suggests a force-driven mechanism of actin polymerization. J. Cell Biol. *167*, 1011–1017.

Lancaster, O.M., Le Berre, M., Dimitracopoulos, A., Bonazzi, D., Zlotek-Zlotkiewicz, E., Picone, R., Duke, T., Piel, M., and Baum, B. (2013). Mitotic rounding alters cell geometry to ensure efficient bipolar spindle formation. Dev. Cell *25*, 270–283.

Legoff, L., Rouault, H., and Lecuit, T. (2013). A global pattern of mechanical stress polarizes cell divisions and cell shape in the growing Drosophila wing disc. Development *140*, 4051–4059.

Lessey, E.C., Guilluy, C., and Burridge, K. (2012). From mechanical force to RhoA activation. Biochemistry *51*, 7420–7432.

Liu, M., Tanswell, A.K., and Post, M. (1999). Mechanical force-induced signal transduction in lung cells. Am. J. Physiol. *277*, L667–L683.

Llinas, P., Isabet, T., Song, L., Ropars, V., Zong, B., Benisty, H., Sirigu, S., Morris, C., Kikuti, C., Safer, D., et al. (2015). How actin initiates the motor activity of myosin. Dev. Cell *33*, 401–412.

Lomakin, A.J., Lee, K.C., Han, S.J., Bui, D.A., Davidson, M., Mogilner, A., and Danuser, G. (2015). Competition for actin between two distinct F-actin networks defines a bistable switch for cell polarization. Nat. Cell Biol. *17*, 1435–1445.

Lyon, R.C., Zanella, F., Omens, J.H., and Sheikh, F. (2015). Mechanotransduction in cardiac hypertrophy and failure. Circ. Res. *116*, 1462–1476.

Mao, Y., and Baum, B. (2015). Tug of war–the influence of opposing physical forces on epithelial cell morphology. Dev. Biol. *401*, 92–102.

Mao, Y., Tournier, A.L., Hoppe, A., Kester, L., Thompson, B.J., and Tapon, N. (2013). Differential proliferation rates generate patterns of mechanical tension that orient tissue growth. EMBO J. *32*, 2790–2803.

Mao, Y.L., Tournier, A.L., Bates, P.A., Gale, J.E., Tapon, N., and Thompson, B.J. (2011). Planar polarization of the atypical myosin Dachs orients cell divisions in Drosophila. Genes Dev. *25*, 131–136.

Marinari, E., Mehonic, A., Curran, S., Gale, J., Duke, T., and Baum, B. (2012). Live-cell delamination counterbalances epithelial growth to limit tissue overcrowding. Nature *484*, 542–545.

Martens, J.C., and Radmacher, M. (2008). Softening of the actin cytoskeleton by inhibition of myosin II. Pflugers Arch. *456*, 95–100.

Martin, A.C., Kaschube, M., and Wieschaus, E.F. (2009). Pulsed contractions of an actin-myosin network drive apical constriction. Nature *457*, 495–499.

Martin-Bermudo, M.D., and Brown, N.H. (1999). Uncoupling integrin adhesion and signaling: the betaPS cytoplasmic domain is sufficient to regulate gene expression in the Drosophila embryo. Genes Dev. *13*, 729–739.

Monier, B., Gettings, M., Gay, G., Mangeat, T., Schott, S., Guarner, A., and Suzanne, M. (2015). Apico-basal forces exerted by apoptotic cells drive epithelium folding. Nature *518*, 245v248.

Munjal, A., Philippe, J.M., Munro, E., and Lecuit, T. (2015). A self-organized biomechanical network drives shape changes during tissue morphogenesis. Nature *524*, 351–355.

Nadkarni, A.V., and Brieher, W.M. (2014). Aip1 destabilizes cofilin-saturated actin filaments by severing and accelerating monomer dissociation from ends. Curr. Biol. *24*, 2749–2757.

Porazinski, S., Wang, H., Asaoka, Y., Behrndt, M., Miyamoto, T., Morita, H., Hata, S., Sasaki, T., Krens, S.F.G., Osada, Y., et al. (2015). YAP is essential for tissue tension to ensure vertebrate 3D body shape. Nature *521*, 217–221.

Pouille, P.A., Ahmadi, P., Brunet, A.C., and Farge, E. (2009). Mechanical signals trigger myosin II redistribution and mesoderm invagination in Drosophila embryos. Sci. Signal. *2*, ra16.

Priya, R., Gomez, G.A., Budnar, S., Verma, S., Cox, H.L., Hamilton, N.A., and Yap, A.S. (2015). Feedback regulation through myosin II confers robustness on RhoA signalling at E-cadherin junctions. Nat. Cell Biol. *17*, 1282–1293.

Riento, K., and Ridley, A.J. (2003). Rocks: multifunctional kinases in cell behaviour. Nat. Rev. Mol. Cell Biol. *4*, 446–456.

Roper, K., Mao, Y., and Brown, N.H. (2005). Contribution of sequence variation in Drosophila actins to their incorporation into actin-based structures in vivo. J. Cell Sci. *118*, 3937–3948.

Schneider, C.A., Rasband, W.S., and Eliceiri, K.W. (2012). NIH Image to ImageJ: 25 years of image analysis. Nat. Methods *9*, 671–675.

Simões Sde, M., Blankenship, J.T., Weitz, O., Farrell, D.L., Tamada, M., Fernandez-Gonzalez, R., and Zallen, J.A. (2010). Rho-kinase directs Bazooka/Par-3 planar polarity during Drosophila axis elongation. Dev. Cell *19*, 377–388.

Singh, A., Saha, T., Begemann, I., Ricker, A., Nüsse, H., Thorn-Seshold, O., Klingauf, J., Galic, M., and Matis, M. (2018). Polarized microtubule dynamics directs cell mechanics and coordinates forces during epithelial morphogenesis. Nat. Cell Biol. *20*, 1126–1133.

Skoglund, P., Rolo, A., Chen, X., Gumbiner, B.M., and Keller, R. (2008). Convergence and extension at gastrulation require a myosin IIB-dependent cortical actin network. Development *135*, 2435–2444.

Stewart, M.P., Helenius, J., Toyoda, Y., Ramanathan, S.P., Muller, D.J., and Hyman, A.A. (2011). Hydrostatic pressure and the actomyosin cortex drive mitotic cell rounding. Nature *469*, 226–230.

Théry, M., Jiménez-Dalmaroni, A., Racine, V., Bornens, M., and Jülicher, F. (2007). Experimental and theoretical study of mitotic spindle orientation. Nature *447*, 493–496.

Vuong-Brender, T.T.K., Ben Amar, M., Pontabry, J., and Labouesse, M. (2017). The interplay of stiffness and force anisotropies drive embryo elongation. Elife *6*.

Wyatt, T., Baum, B., and Charras, G. (2016). A question of time: tissue adaptation to mechanical forces. Curr. Opin. Cell Biol. *38*, 68–73.

Wyatt, T.P., Harris, A.R., Lam, M., Cheng, Q., Bellis, J., Dimitracopoulos, A., Kabla, A.J., Charras, G.T., and Baum, B. (2015). Emergence of homeostatic epithelial packing and stress dissipation through divisions oriented along the long cell axis. Proc. Natl. Acad. Sci. USA *112*, 5726–5731.

Zartman, J., Restrepo, S., and Basler, K. (2013). A high-throughput template for optimizing Drosophila organ culture with response-surface methods. Development *140*, 667–674.

Zhang, L., and Ward, R.E., 4th (2011). Distinct tissue distributions and subcellular localizations of differently phosphorylated forms of the myosin regulatory light chain in Drosophila. Gene Expr. Patterns *11*, 93–104.

Zhou, J., Kim, H.Y., and Davidson, L.A. (2009). Actomyosin stiffens the vertebrate embryo during crucial stages of elongation and neural tube closure. Development *136*, 677–688.

STAR★METHODS

KEY RESOURCES TABLE

REAGENT or RESOURCE	SOURCE	IDENTIFIER
Antibodies		
Donkey anti-mouse RRX	Jackson Immuno Research	115-295-003; RRID: AB_2338756
Goat anti-GFP-FITC	Abcam	ab6662; RRID: AB_305635
Goat anti-mouse Alexa-488	Life Technologies	A11029; RRID: AB_138404
Goat anti-rabbit Alexa Fluor-555	Abcam	ab150086; RRID: AB_2722519
Goat anti-rabbit Alexa-488	Life Technologies	A11034; RRID: AB_2576217
Mouse anti-Engrailed	Developmental Studies Hybridoma Bank	4D9; RRID: AB_528224
Rabbit anti-Diaphanous	(Afshar et al., 2000)	N/A
Rabbit Beta-Galactosidase	Life Technologies,	A11132; RRID: AB_221539
Rabbit pMRLC	Cell Signalling	3671S; RRID: AB_330248
Chemicals, Peptides, and Recombinant Proteins		
DAPI	Sigma-Aldrich	D8417; RRID: AB_2307445
Fluoromount G slide mounting medium	SouthernBiotech	0100-01
GelPak, 6.5 mil	GelPak	PF-70-x4/6.5mil
Microposit^{TM} EC solvent	Dow Chemicals	
Phalloidin Alexa-488	Life technologies	A12379; RRID: AB_2315147
Phalloidin Alexa-555	Life technologies	A34055
Phalloidin Alexa-647	Life Technologies	A22287; RRID: AB_2620155
Su-8 2050	Microchem	Su-8 2050
SYLGARD 184 elastomer kit	Dow Corning	1673921
Y27632 dihydrochloride Rok Inhibitor	Sigma	Y0503
Experimental Models: Drosophila melanogaster		
actin-FRT-stop-FRT-Gal4-UAS-Cd8::mCherry; Arm::GFP	Y. Mao	N/A
actin-FRT-stop-FRT-Gal4-UAS-Cd8::mCherry; Rho-1::GFP	Y. Mao	N/A
actin-FRT-stop-FRT-Gal4-UAS-Cd8::mCherry; RokK116a::Venus	Y.Mao from Rokk116a::Venus (Simões et al., 2010)	N/A
actin-FRT-stop-FRT-Gal4-UAS-Cd8::mCherry; Sqh::GFP	Y. Mao	N/A
actin-FRT-stop-FRT-Gal4-UAS-Cd8::mCherry; TubGal80^{ts}	Y. Mao	N/A
DEcad::GFP; rnGal4	N. Tapon	N/A
ECad::6XRFP	Gift from Y. Bellaiche	N/A
Ecad::GFP; pnrGal4, Sqh::mCherry	Ecad::GFP knock-in (Huang et al., 2009). Gift from B. Baum	N/A
Ecad::tomato	(Huang et al., 2009)	N/A
mRFP.nls,w,hsF,Frt19A	Bloomington Drosophila Stock Centre	#31418
Rho-1::GFP	Kyoto Stock Centre	DGRC ID: 110833
Sqh::GFP, enGal4	Y. Mao	N/A
Sqh::GFP, enGal4; UAS-Act5C::RFP	Y. Mao	N/A
Sqh::mCherry	E. Wieschaus	N/A
SqhAx3;;Sqh::GFP	Jordan and Karess, (1997)	N/A
UAS-Act5C::RFP	K. Roper (Roper et al., 2005)	N/A
UAS-AIP1 ^{RNAi}	Vienna *Drosophila* RNAi Centre	VDRC, ID: 22851
UAS-arp2^{RNAi}	Vienna *Drosophila* RNAi Centre<br>Fly stocks of National Institute of Genetics	VDRC:KK10199<br>NIG, ID: 9901-R
UAS-Cd8::GFP, nubGal4	Mao et al. (2013)	N/A

(*Continued on next page*)

Continued

REAGENT or RESOURCE	SOURCE	IDENTIFIER
UAS-cofilin ^{RNAi}	Vienna *Drosophila* RNAi Centre	VDRC, ID: 110599
UAS-Diaphanous ^{RNAi}	Vienna *Drosophila* RNAi Centre	VDRC:KK103914 20518 GD
UAS-drak ^{RNAi}	Vienna *Drosophila* RNAi Centre	VDRC, ID:107263
UAS-e-cadherin ^{RNAi}	Vienna *Drosophila* RNAi Centre	VDRC:KK103962
UAS-enabled ^{RNAi}	Vienna *Drosophila* RNAi Centre	VDRC, ID: 43058
UAS-mlck ^{RNAi}	Vienna *Drosophila* RNAi Centre	VDRC, ID:109937KK
UAS-paxillin ^{RNAi}	Vienna *Drosophila* RNAi Centre	VDRC, ID:107789
UAS-pebble ^{RNAi}	A. Mueller	N/A
UAS-rho kinase ^{RNAi}	Vienna *Drosophila* RNAi Centre	VDRC:KK104675
UAS-rho ^{RNAi}	Vienna *Drosophila* RNAi Centre	VDRC, ID:109420 KK
UAS-spaghetti squash ^{RNAi}	Fly stocks of National Institute of Genetics	NIG, ID: HMS00830 and HMS00437
UAS-Sqh[AA]/TM6B	B. Baum	N/A
UAS-src42A ^{RNAi}	N. Tapon	N/A
UAS-src64B ^{RNAi}	N. Tapon	N/A
UAS-vinculin ^{RNAi}	Vienna *Drosophila* RNAi Centre	VDRC, ID: 34586
UAS-zyxin ^{RNAi}	Fly stocks of National Institute of Genetics	NIG ID: 32018R-1 and 32018R-3
UAS-α*-actinin* ^{RNAi}	Vienna *Drosophila* RNAi Centre	VDRC, ID:107263
UAS–torso^{D}*/*β*cyt*	Martin- Bermudo and Brown, (1999)	N/A
Ubi-dia::GFP	P. Gaspar and N. Tapon	N/A
ubi-dia::GFP; actin-FRT-stop-FRT-Gal4-UAS-Cd8::mCherry/CyO	Y. Mao	N/A
ubi-Ecad::GFP; hhGal4, UAS-IAP/TM6; Sqh::mCherry	(II) - N. Tapon<br>(III) - (Martin et al., 2009)	N/A
ubi-mRFP.nls,w,hs.flp,Frt19A;; Arm-GFP	Y. Mao	N/A
ubi-mRFP.nls,w,hs.flp,Frt19A;; Sqh-GFP	Y. Mao	N/A
yw hsflp; dia iR	Y. Mao from VDRC:103914 KK	N/A
yw hsflp; rho iR	Y. Mao from VDRC:109420/KK	N/A
yw hsflp; rok iR	Y. Mao from VDRC:KK104675	N/A
yw hsflp; UAS-zip iR/SM6-TM6	Y. Mao	N/A
yw hsflp; zip iR; Arm::GFP/SM6-TM6	Y. Mao	N/A
yw,Drok^{2},Frt19A/FM7c	Bloomington Drosophila Stock Centre	#6666
yw; act-FRT-stop-FRT-Gal4-UASlacZ/ CyO	Y. Mao	N/A
Software and Algorithms		
Epitools	Heller et al. (2016) https://epitools.github.io/wiki/	N/A
GraphPad Prism 7	GraphPad Software	N/A
ImageJ 1.51w	Schneider et al. (2012) https://imagej.nih.gov/ij/docs/install/osx.html	N/A
Vertex model	Bespoke model code developed in C++, as in Mao et al. (2013).	N/A

CONTACT FOR REAGENT AND RESOURCE SHARING

Further information and requests for resources and reagents should be directed to and will be fulfilled by the Lead Contact, Yanlan Mao (y.mao@ucl.ac.uk).

EXPERIMENTAL MODEL AND SUBJECT DETAILS

Drosophila melanogaster

Fly stocks were raised in non-crowded conditions on standard cornmeal molasses fly food medium at 25°C, unless otherwise indicated. Briefly, the fly food consisted of, per 1L, 10g agar, 15g sucrose, 33g glucose, 35g years, 15g maize meal, 10g wheat germ, 30g treacle, 7.22g soya flour, 1g nipagin, 5ml propionic acid. Male and female larvae were dissected at late 3rd instar development (approximately 110-120hr AEL) for experiments.

Strains used are listed in the Key Resources Table and include: *Ecad::GFP* (knock-in (Huang et al., 2009)); *pnrGal4, Sqh::mCherry* (gift from Baum lab), *Ecad::Tomato* (Huang et al., 2009), *Sqh::GFP, enGal4; UAS-Act5C::RFP* (self-generated), *Rho1::GFP* (protein trap, Kyoto Stock Centre, DGRC ID: 110833), *SqhAx3;; Sqh::GFP* (Jordan and Karess, 1997), *Rok^{K116a}::Venus* (Simões et al., 2010), *ubi-Ecad::GFP; hhGal4, UAS-IAP/TM6* (gift from N. Tapon), *Sqh::mCherry* (III) (Martin et al., 2009), *UAS-Cd8::GFP, nubGal4* (Mao et al., 2013), *UAS–torso^D/βcyt (Myospheroid mutant)* (Martin-Bermudo and Brown, 1999), u*bi-mRFP.nls,w,hsF,Frt19A* (Bloomington, 31418), *yw,Drok^2,Frt19A/FM7c* (Bloomington, 6666) (Escudero et al., 2007), *ubi-mRFP.nls,w,hs.flp,Frt19A;; Arm-GFP* (self-generated), *ubi-mRFP.nls,w,hs.flp,Frt19A;; Sqh-GFP* (self-generated), *actin-FRT-stop-FRT-Gal4-UAS-Cd8::mCherry; Arm::GFP* (self-generated), *actin-FRT-stop-FRT-Gal4-UAS-Cd8::mCherry; Sqh::GFP* (self-generated), *actin-FRT-stop-FRT-Gal4-UAS-Cd8::mCherry; Rok^{K116a}::Venus* (self-generated), *actin-FRT-stop-FRT-Gal4-UAS-Cd8::mCherry; Rho-1::GFP* (self-generated), *ubi-dia::GFP* (gift from Pedro Gaspar and Nic Tapon), *ubi-dia::GFP; actin-FRT-stop-FRT-Gal4-UAS-Cd8::mCherry/CyO* (self-generated), *yw hs.flp; UAS-zip iR/SM6-TM6* (self-generated), *yw hs.flp; UAS-rho iR/SM6-TM6* (self-generated from KK/109420), *yw hs.flp; UAS-dia iR/SM6-TM6* (self-generated from KK/103914), *yw hs.flp; UAS-rok iR/SM6-TM6* (self-generated from KK/104675), *yw hs.flp UAS-zip iR; ArmGFP/SM6-TM6* (self-generated), *actin-FRT-stop-FRT-Gal4-UAS-Cd8::mCherry; TubGal80^{ts}* (self-generated), *DEcad-GFP; rotundGal4* (gift from Tapon lab), *UAS-Sqh[AA]/TM6B* (gift from Baum lab), *ECad::6xRFP* (gift from Bellaiche lab).

For the generation of *Drok^2* mitotic clones *ubi-mRFP.nls,w, hs.flp,Frt19A/ yw,Drok^2,Frt19A*; *Arm-GFP/+* or *ubi-mRFP.nls,w,hsF,Frt19A/ yw,Drok^2,Frt19A; Sqh-GFP/+* larvae were heat shocked at 37 °C for 1h at the age 48h AEL and discs were dissected for experiments 72h later (at 120h AEL).

For the generation of flipout clones expressing *yw hs.flp/+; actin-FRT-stop-FRT-Gal4-UAS-Cd8::mCherry /zipiR; arm-GFP/+, yw hs.flp/+; actin-FRT-stop-FRT-Gal4-UAS-Cd8::mCherry /zip iR; Sqh-GFP/+, yw hs.flp/+; actin-FRT-stop-FRT-Gal4-UAS-Cd8::mCherry/zip iR; Rok^{K116a}::Venus/+; yw hs.flp/+; actin-FRT-stop-FRT-Gal4-UAS-Cd8::mCherry /zip iR; Rho-1::GFP/+, yw hs.flp/+; actin-FRT-stop-FRT-Gal4-UAS-Cd8::mCherry /dia iR; arm-GFP/+, yw hs.flp/+; actin-FRT-stop-FRT-Gal4-UAS-Cd8::mCherry /dia iR; Sqh-GFP/+, yw hs.flp/+; actin-FRT-stop-FRT-Gal4-UAS-Cd8::mCherry /dia iR; Rok^{K116a}::Venus/+, yw hs.flp/+; actin-FRT-stop-FRT-Gal4-UAS-Cd8::mCherry /dia iR; AniRBD::GFP/+, yw hs.flp/+; actin-FRT-stop-FRT-Gal4-UAS-Cd8::mCherry /dia iR; Rho-1::GFP/+, yw hs.flp/+; actin-FRT-stop-FRT-Gal4-UAS-Cd8::mCherry /rok iR; arm-GFP/+, yw hs.flp/+; actin-FRT-stop-FRT-Gal4-UAS-Cd8::mCherry /rok iR; Sqh-GFP/+, yw hs.flp/+; actin-FRT-stop-FRT-Gal4-UAS-Cd8::mCherry /rok iR; Rho-1::GFP/+ and yw hs.flp/ ubi-Dia::GFP; actin-FRT-stop-FRT-Gal4-UAS-Cd8::mCherry /rok iR* larvae were heat shocked at 37 °C for 13 min at the age 62-72h AEL and discs dissected for experiments 48h later (at 110-120h AEL). For *yw hs.flp/+; actin-FRT-stop-FRT-Gal4-UAS-Cd8::mCherry /rho iR; Sqh-GFP/+; yw hs.flp/ ubi-Dia::GFP; actin-FRT-stop-FRT-Gal4-UAS-Cd8::mCherry /rho iR, yw hs.flp/+; actin-FRT-stop-FRT-Gal4-UAS-Cd8::mCherry /rho iR; Rok^{K116a}::Venus* /+, in which, strong Rho knock down leads to clone extrusion, larva were heat shocked at 37 °C for 13 min at the age 72-96 AEL and grown at 18 °C for 72 hours before discs were dissected for experiments at the equivalent age of 96-120h AEL at 25°C. For Silent clone generation with *zipper* RNAi, *yw hs.flp/+; actin-FRT-stop-FRT-Gal4-UAS-Cd8::mCherry/zip iR; ArmGFP/ TubGal80^{ts}* larva were heat shocked at 37°C for 13 min at the age 48-72h AEL and kept at 18°C for 60 hours before shifting larva to 29°C for 16 hours. Discs were dissected and selected for morphology equivalent to 96-120h AEL at 25°C.

All RNAi lines were purchased from Vienna *Drosophila* Resource Centre (VDRC) or National Institute of Genetics (NIG). The RNAi lines were used to silence following genes: *zipper* (VDRC, ID: 7819), *spaghetti squash* (NIG, ID: HMS00830 and HMS00437), *diaphanous* (VDRC, ID: 103914 KK and 20518 GD), *pebble* (gift from A. Mueller), *arp2* (VDRC, ID: 101999 and NIG, ID: 9901-R), *rok* (VDRC, 104675KK), *enabled* (VDRC, ID: 43058), *e-cadherin* (VDRC, ID: 103962), *drak* (VDRC, ID:107263), *src64B* (gift from N.Tapon), *src42A* (gift from N.Tapon), *zyxin* (NIG ID: 32018R-1 and 32018R-3), *mlck* (VDRC, ID:109937KK), *paxillin* (VDRC, ID:107789), *vinculin* (VDRC, ID: 34586), *α-actinin* (VDRC, ID:107263), *cofilin* (VDRC, ID: 110599), *AIP1* (VDRC, ID: 22851), rho (VDRC, ID:109420 KK).

METHOD DETAILS

Fabrication of Silicon Master Mold

Optical lithography was performed on silicon wafers and carried out using the cleanroom facilities of the London Centre for Nanotechnology. Silicon substrates were cleaned through repeated washes with acetone and isopropanol, and covered with Su-8 2050 (Microchem). The photoresist was spun with a spin-coater, according to the manufacturer's instructions, to achieve a uniform thickness of 50μm. The soft-bake step, required after spin-coating to evaporate the excess of solvent and to densify the resist, was performed by placing the wafers for 3 minutes on a hot-plate at 65°C and, sequentially, at 95 °C for 8 minutes. The photoresist is exposed to a pattern of intense UV light through a photomask. The UV exposure time needed to crosslink the SU-8 was calculated by dividing the exposure energy (mJ/cm^2) indicated in the Su-8 data sheet by the light intensity of the Karl Suss MJB3 mask aligner (20 mJ/cm^2). Post-exposure bake was performed on hot-plates, at 65°C (2 minutes) and at 95°C (8 minutes). The wafer was placed in

Microposit^{TM} EC solvent for development for approximately 6 minutes, and finally hard-baked at 180°C for 5 minutes, to stabilize and harden the developed photoresist.

Preparation of Patterned Polydimethylsiloxane (PDMS) Membranes

PDMS mix was prepared according to the manufacturer's recommendations (SYLGARD 184 elastomer kit, Dow Corning), briefly spinned down to remove bubbles (2min, 1000rpm) and let to cure at room temperature for 4h prior to spinning (30 seconds, 900 rpm) on a patterned silicon wafer mold (microchannels of 80-120μm width and 50μm depth) with Spin Coater (SPS Spin 150). The PDMS covered mold was then heated up to 100° C for 10 min to allow full PDMS curing and the PDMS was subsequently peeled off the wafer and transferred onto a transparency sheet for storage.

Stretcher Design

A custom made stretching/compression device was constructed. In detail, the basic drive for the unit consists of a combination of linear bearings with left and right-handed fine pitch screw threads to provide the desired movement. The extended metal arms connect the drive to the clamping mechanism. Moving the arms apart (inside-out) or bringing them closer together (outside-in) respectively stretches or compresses the clamped material. The device fits onto most standard microscope stage plate holders suitable for imaging.

For stretching/compression assays, a pre-patterned PDMS membrane (Figure 1B) was spread over two metal arms, overlaid with a plain (unpatterned) PDMS membrane (GelPak, 6.5 mil) and clamped on both ends. An isolated wing disc was suspended in the media, injected in between two PDMS layers and positioned with forceps over a microchannel, with its apical side of wing disc proper facing down the channel. A PDMS chamber was placed on top of the membranes and filled with media to prevent the drying out of the sample.

Stretching Experiments

If not specified otherwise stretching was performed bi-directionally along anterior-posterior (A-P) axis. An anchored disc was defined as a disc injected in between PDMS membranes but not subjected to any mechanical perturbation. Short stretch was defined as less than 30 minutes and long stretch as more than 120 min. Relaxation was achieved by bringing the stretcher arms back to the pre-stretch position. For assessment of degree of polarity with respect to applied strain (Figure 2) discs were stretched to increasing degrees with 5 min pause intervals in between to allow for the full emergence of MyoII polarization. We confirmed stretching had no effect on discs viability as cells continued dividing at least 1 hour post-stretch (see Video S3).

Immunofluorescence

Discs were dissected in ice cold PBS and fixed for 30 min in 4% paraformaldehyde, washed 4x10 min with PBT (PBS, 0.3% Triton X-100), blocked for 1h with 0.5% BSA PBT and stained with primary (over night at 4°C), washes were repeated before incubating in fluorescently conjugated secondary antibodies (1h at room temperature) and were washed 3x20 min PBT, 3xquick wash PBS. Discs were mounted using Fluoromount G slide mounting medium (SouthernBiotech) ready for imaging.

In cases where immunofluorescence staining was required for stretched discs and their controls, discs were directly fixed on the device by exchanging media with 4% PFA for 15 min, then discs were removed from the device and fixed for a further 15 min in a glass well. Staining protocol is the same as above. When immunostaining specifically for phospho-MyoII, 18% PFA was used directly on the stretcher device for 5 minutes, removed and fixed for a further 5 minutes, the washes as stated above were doubled for the staining protocol previously described.

Primary antibodies used were rat anti-E-cad (Developmental Studies Hybridoma Bank), rabbit anti-Diaphanous (Afshar et al., 2000), mouse anti-Engrailed (Developmental Studies Hybridoma Bank), mouse anti-Sqh (gift from R. Ward) (Zhang and Ward, 2011), rabbit anti-phospho-Sqh (3671, Cell Signalling) goat anti-GFP-FITC (Abcam, ab6662). Dyes used were DAPI (D8417, Sigma) for DNA staining and Phalloidin Alexa-488 (A12379, Life technologies), Phalloidin Alexa-555 (A34055, Life technologies) or Phalloidin Alexa-647 (A22287, Life Technologies) for actin labeling. All secondary antibodies were from Life Technologies and Jackson ImmunoResearch.

Young's Modulus Determination

Force measurement device used for Young's modulus determination was modified from previous design (Harris et al., 2012; Wyatt et al., 2015). Briefly, a glass capillary (O.D. 1 mm, I.D. 0.5 mm, Sutter Instruments) was heated in its centre using a micro-pen blowtorch and bent into a right angle. This procedure was repeated in a second position separated by ~10 mm from the first bend. The capillary was cut to size using a pair of pliers, such that the lengths of its arms were 35 and 5 mm. A 35 mm strand of NiTi wire (0.3 mm diameter, Euroflex) was cut, dipped into UV-curing glue (Loctite Glassbond, Henkel), and threaded into the short capillary arm. The capillary was then exposed to UV light for 5 min. A second ~10 mm length of capillary was then cut, glued to the free end of the NiTi wire and cured with UV light for 5 min. This would act as a flexible rod, whereas the long capillary arm would act as a static rod. Next, two glass coverslips (VWR) were cut to size (~10x10 mm) with a glass scorer, glued to the extremities of the static and flexible rods, and cured for 30 min. The device was then submerged in 70% ethanol for 5 min, rinsed with water and left to air dry for 10 min. Finally,

the device was secured to the bottom of a plastic Petri dish (50x20.3 mm high-rimmed Petri dish, Thermo Fisher Scientific) using hot glue. The mechanical testing setup was modified to allow direct force measurements with force transducer rather than inferring it from the bending of the wire in the images.

A wing disc was immobilized with CellTak (Corning) over two coverslips attached to flexible and static rods and subsequently overlaid with culture media. Stretching force was applied with a motorized micromanipulator (Physik Instrumente). Initially, the wing disc was preconditioned, by stretching to 0.1mm at 0.01mm/s strain rate. This was repeated 5 times. Then the disc was rested for 2.5 min to allow full relaxation. The stretching experiments were conducted by stretching the discs to 0.3mm, 0.5mm. 0.65mm and 0.8mm at 0.01mm/s strain rate. The wire was calibrated by stretching it to 0.8mm at 0.01mm/s strain rate. This was repeated 3 times. For Young's modulus determination the recorded force and measured cross sectional area (depth dimension averaged as 50 μm; width measured in each of experiments) was used to derive stress. The Young's modulus was further determined from the linear part of the stress-strain curve.

Live Imaging

For all live imaging experiments discs were cultured *ex vivo* as described previously (Heller et al., 2016; Zartman et al., 2013). Live imaging of stretching and compression experiments was performed on a Zeiss Spinning Disc confocal microscope equipped with Andor Zyla 4.2. PLUS sCMOS camera or Zeiss LSM880 inverted multiphoton microscope. The field of view for imaging was confined to the pouch and part of the hinge region of the wing disc with a 40x lens and 1 μm depth resolution. The LSM880 Airyscan detector was used for imaging MyoII and ECad in stretched discs analyzed for acute tension release, *ubi-Dia-GFP* imaging and Rok inhibitor treated discs and depth resolution was optimised to 0.2 μm.

Fixed Sample Imaging

Fluorescent imaging of fixed samples was performed on Leica Sp5 and Sp8 inverted confocal microscopes. Images of discs that had not been within the stretcher device were acquired with 40x objective and depth resolution of 1μm. Images obtained of anchored and stretched discs from clones and phosphor-MyoII experiments were acquired with 40x objective and depth resolution of 0.35μm. High resolution actin images were obtained on Leica SP8 using 100X objective, pixel resolution of 49-51nm and depth resolution of 0.13-0.15 μm as calculated for optimal deconvolution with Huygens Professional software.

Laser Ablations

Drok2 Clones

Laser ablations were performed on a Zeiss LSM780 inverted two-photon microscope with a Chameleon Ultra II laser set to 730 nm and 100% power with ~16 μs dwell time and × 10–12 digital zoom with 40x lens. Timelapse was recorded for a single channel (E-cad::GFP) with 780 ms scan time. For each of the experiments the overview picture was taken (E-cad::GFP, RFP) to identify the position of the clones prior to laser cutting; care was taken to take comparative regions in the disc for control and *DRok*^{2} clones.

Stretching Experiments

Laser ablations were performed on a Zeiss LSM880 inverted multiphoton microscope with a Chameleon Ti:Sapphire laser set to 720 nm and 60% power and the appropriate digital zoom ranging from 10x–5x. The single junction ablations were acquired with a Zeiss Airyscan detector set to a super-resolution mode. Timelapse was recorded for a single E-cad::GFP channel with 633.02 ms (confocal imaging) or 643.47 ms (superresolution imaging) scan time. Single junction ablations were analyzed as described previously (Mao et al., 2013). For tissue scale tension measurements, an ellipse was fitted over the ablated region with Fiji. The change in short ellipse axis over time was taken as a measure of tissue recoil (Figure S6A.). For determining tissue-scale ablation recoil (Figures 7A–7E) a single junction was ablated and timelapse was recorded for a single E-cad::GFP channel with 643.47 ms scan intervals. Based on the resultant videos the area was drawn around 0,1,2, and 3 rows of cells away from injured cells prior to ablation (0 sec.) and post ablation (15.4 sec.) (Figure 7B). The difference in area normalized to pre-ablation area was taken as a recoil propagation measure (post ablation area – pre-ablation area /pre-ablation area). If required the images were corrected with stackreg Fiji plugin to account for unspecific drift. For acute tension release mediated by a large tissue cut, polarity was calculated for the pre-ablation disc (8-15min stretch) and then following ablation imaged at multiple time points up to purse string formation. Polarity was quantified for the time point in which the cut had stopped recoiling and preceded purse string formation (approx. 10 min).

Screen Design

Sqh::GFP, enGal4> UAS-Act5C::RFP line was generated for screening and crossed to respective RNAi or overexpression lines. Discs were imaged in anchored and stretched (15 min) configuration. A hit was defined on the basis of Sqh::GFP localization: uniform junctional when anchored on both posterior and anterior sides; polarized on anterior side (control) and uniform on posterior side (RNAi) when stretched.

Rok Inhibition

For Rok inhibition, Y27632, 30mM stock was diluted to make a 1mM and 100μM treatment in dissecting medium. Discs were incubated for 30 minutes prior to loading into the stretcher and imaging as described.

Fluorescence Recovery after Photobleaching

Sqh::GFP, enGal4/+; UAS-Act5C::RFP + and *Sqh::GFP, enGal4/UAS-dia iR; UAS-Act5C::RFP* lines were generated for FRAP analysis. Acquisition and bleaching of Sqh-GFP and Act5C::RFP was acquired using a Zeiss LSM880 inverted multiphoton microscope with a Chameleon Ti:Sapphire laser. Acquisition was obtained at 5X digital zoom and bleaching induced by 860 nm laser at 10-15% power to a region of 300 by 30 pixels or 30 by 300 pixels (16.03μm by 1.37μm and vice versa) that spanned multiple horizontal and vertical junctions. The acquisition region was imaged prior to bleaching for normalisation. Then, following bleaching, recovery was imaged at 5 second intervals up to 250 seconds.

The Vertex Model

The effects of local myosin responses at the cellular level on the emergent shape control of the whole tissue is investigated using the well-established vertex model (Farhadifar et al., 2007; Mao et al., 2011).

The vertex model defines the epithelia through the cell-cell contact edges. Each cell is defined by the polygons formed through the cellular junction points (vertices), and cell-cell boundaries connecting these junction points (edges). The edges represent the attached contacting surfaces of cells on each side. The physical properties of the edges represent a combined effect of cell-cell adhesion and actomyosin cortex. The model describes the total energy of the system as a function of cell-cell junction vertex positions. The vertex positions are updated during the simulation to minimize the total energy, and the dynamics of the epithelial morphology emerges in the process.

The energy of the system is defined by the combined energy contributions of: i) cell area conservation, the deviation of each cell from its ideal size, ii) combination of tension and adhesion energies at each contacting cell-cell boundary (1).

$$E(\boldsymbol{R}_i) = \underbrace{\sum_{\alpha}^{N_c} \frac{K_\alpha}{2}\left(A_\alpha - A_\alpha^{(0)}\right)^2}_{\text{(i)}} + \underbrace{\sum_{i,j}^{N_v} \Lambda_{ij} l_{ij}}_{\text{(ii)}} \qquad \text{(Equation 1)}$$

In Equation 1, $E(R_i)$ represents the total energy of the system for the vertex positions $\boldsymbol{R_i}$. The modelled epithelia is composed of $\boldsymbol{N_c}$ cells (α = 1…$\boldsymbol{N_c}$), and $\boldsymbol{N_v}$ vertices (i = 1…$\boldsymbol{N_v}$). In area conservation energy contribution (i), K_α is the elasticity coefficient, A_α is the current area, and $A^{(0)}{}_\alpha$ is the ideal area of cell α. For the tensile/adhesive contact energy contribution (ii), Λ_{ij} is the line tension coefficient for the vertex couple (i,j), and l_{ij} is the length of the edge between vertices. In the simulations of this manuscript, the base tension is Λ_{ij} = 0.26, $A^{(0)}{}_\alpha$ is 1.0, and K_a is 1.0.

Further to the definition of the total energy of the system, cell growth is modelled to have an average 4 hour G0 phase. Cell growth prior to division is driven by increasing the ideal area of the cell ($A^{(0)}$). The cells are allowed to divide only if they can increase their emergent size above a certain threshold within the limitations of energy minimization, division is aligned to the longest axis of the cell, within a random margin of 30 degrees. The system is defined as overdamped, such that the forces obtained from the derivative of the energy function move the cells against a global viscosity, defined as 1 units per second. For further details of the modeling methodology, refer to (Mao et al., 2011).

Modeling the myosin response

The myosin response upon stretch is defined as a percentage increase on the tension term Λ_{ij} for the edges where the response is applicable, such that a 5 percent myosin response is defined as a 5 percent increase from the base parameter value of 0.26. The edges that demonstrate the myosin response are limited to edges aligned at ±30 degrees to the stretched axis.

Tissue stretching simulations

Random epithelia are generated by growing a small number of cells into an epithelial sheet of approximately 1500 cells. For application of stretch, the lateral regions of the generated epithelial sheet further than ten micrometres away from the epithelia centre are clamped, and each vertex within this region is pulled by a predefined stretching force $\mathbf{F}_s$ throughout the simulation. The orientation of the applied force is parallel to the x-axis, its direction pointing away from the centre of the sheet, depending on the position of the vertex. The applied force is selected to be 2.5E-5 units per vertex in the simulations of this manuscript. The precise value of the applied force is not deterministic of the effects of the myosin response, as long as the selected value is sufficiently high to stretch the tissue, yet not too high to cause tearing of the sheet.

For each randomly generated epithelial sheet, simulations are run with and without the myosin response, with the same level of tissue stretch. The progeny of the cells within 8 micrometres of the tissue centre at the beginning of each simulation, therefore outside the clamped zone, are traced. The aspect ratios of the emerging clones at the end of 12 hour simulations are analyzed as a readout for the effects of cellular level on emergent tissue morphology. The statistical difference is tested by a two-tailed t-test on data from 10 simulations for each case, with and without the myosin response.

QUANTIFICATION AND STATISTICAL ANALYSIS

Analysis of Junctional Intensities and Cell Aspect Ratio

Images were segmented and analyzed with EpiTools software as reported before (Heller et al., 2016). In short, image was segmented (Epitools) after background subtraction (rolling ball radius 20, Fiji) and underlying junctional intensities (E-cad::GFP and

Sqh::mCherry) were extracted with Cell Graph (EDGE_COLOR_TAG overlay) (Figure S2). The following parameters were applied: 2 pixel edge intensity buffer around the junctions, selection mode 1 (tricellular signal removed), upper 50% (0.5) of the signal intensity. Junctions were selected manually and color-coded as horizontal (junction angle <45°) and vertical (junction angle >45°); resultant intensity data was exported. Polarity was defined as fluorescent mean intensity on horizontal (stretched or equivalent in anchored) junctions divided by intensity on vertical (non-stretched or equivalent in anchored) junctions. Total MyoII and E-cad was quantified by multiplication of mean intensity by the junctional length. Concentration was defined as mean fluorescent intensity per unit junctional area. For aspect ratio the analysis was performed as shown previously using ELLIPSE_ELONGATION_RATIO or CELL_COLOR_TAG overlay in Cell Graph (Heller et al., 2016).

Fluorescence Recovery after Photobleaching Analysis

Intensity recovery curves were fitted with the following function, where bleaching is conducted at t = 0s:

$$I(t) - I(0) = [I_\infty - I(0)]\left(1 - e^{-t/\tau}\right)$$

Mobile fraction was defined as $[I_\infty - I(0)]/[I_{pre-bleach} - I(0)]$ and half time as $\tau \ln 2$.

Due to non-normality of the data, Wilcoxon rank-sum test was used for statistical analysis.

Anisotropy Analysis

To quantify anisotropy of actin structures, small regions of interst containing ~10 cells were chosen from the raw microscopy images, insuring that all cells were in focus. Anistropy was then calculated for three planes at the level of the adherens junctions. For this, we used FibrilTool(Boudaoud et al., 2014), an ImageJ plugin developed to quantify fibrillar structures in microscopy images. Briefly, the plugin calculates a local nematic tensor $n = t \otimes t$, where t is a function of the pixel intensity $I(x,y)$:

$$t = \left(t_x, t_y\right) = (\partial I/\partial y, -\partial I/\partial x) \Big/ \sqrt{(\partial I/\partial x)^2 + (\partial I/\partial y)^2}$$

The local nematic tensor is then averaged over the region of interest to achieve an average nematic tensor $<n>$. If n_1 and n_2 are the eigenvalues of $<n>$ such that $n_1 > n_2$, then anisotropy is defined as $q = n_1 - n_2$.

Finally, the anisotropies of the three planes were averaged to achieve a single anisotropy for each ROI.

Wilcoxon rank-sum test was used for statistical analysis.

Wing Disc Pouch Shape Analysis

nubGal4>UAS-Cd8::GFP was used to define the areas of the wing disc pouch and to drive RNAi. An ellipse was fitted with Fiji selectively to the pouch region and aspect ratio (length of ellipse long axis/length of ellipse short axis) determined.

Statistical Analysis

No statistical methods were used to predetermine sample size. The minimum requirements for sample sizing were as follows: 1. Experiments performed on 3 independent days; 2. Wing discs from at least 3 independent animals/conditions; 3. On average 20-200 cells were analyzed per wing disc. Exclusion criteria, mostly due to Sqh-mCherry aggregates: 1. Uneven and unspecific fluorescent signal across the sample; 2. Much higher variability when compared to other samples in the same condition group. Random wing discs were chosen within a given condition group.

Prism 7 was used for statistically analysis. Two-tailed unpaired or paired Student's t-tests were used as indicated in the respective figure legends along with the exact n values used for each of the experiments. The following statistical significance cut off was applied: * p<0.05, **p<0.01, ***p<0.001, ****p<0.0001.

Developmental Cell, Volume *48*

Supplemental Information

Polarization of Myosin II Refines

Tissue Material Properties

to Buffer Mechanical Stress

Maria Duda, Natalie J. Kirkland, Nargess Khalilgharibi, Melda Tozluoglu, Alice C. Yuen, Nicolas Carpi, Anna Bove, Matthieu Piel, Guillaume Charras, Buzz Baum, and Yanlan Mao

Supplemental Information

INVENTORY OF SUPPLEMENTAL INFORMATION

Supplemental Data

Figure S1, related to Figure 1
Figure S2, related to Figure 2
Figure S3, related to Figure 4
Figure S4, related to Figure 4
Figure S5, related to Figure 5
Figure S6, related to Figure 6
Figure S7, related to Figure 6 and 7

Supplemental Movies Legends

Movie S1, related to Star Methods: Young's modulus determination
Movie S2, related to Figure 1
Movie S3, related to Figure 2
Movie S4, related to Figure 6
Movie S5, related to Figure 6
Movie S6, related to Figure 4

Duda et al. Fig. S1 related to Figure 1

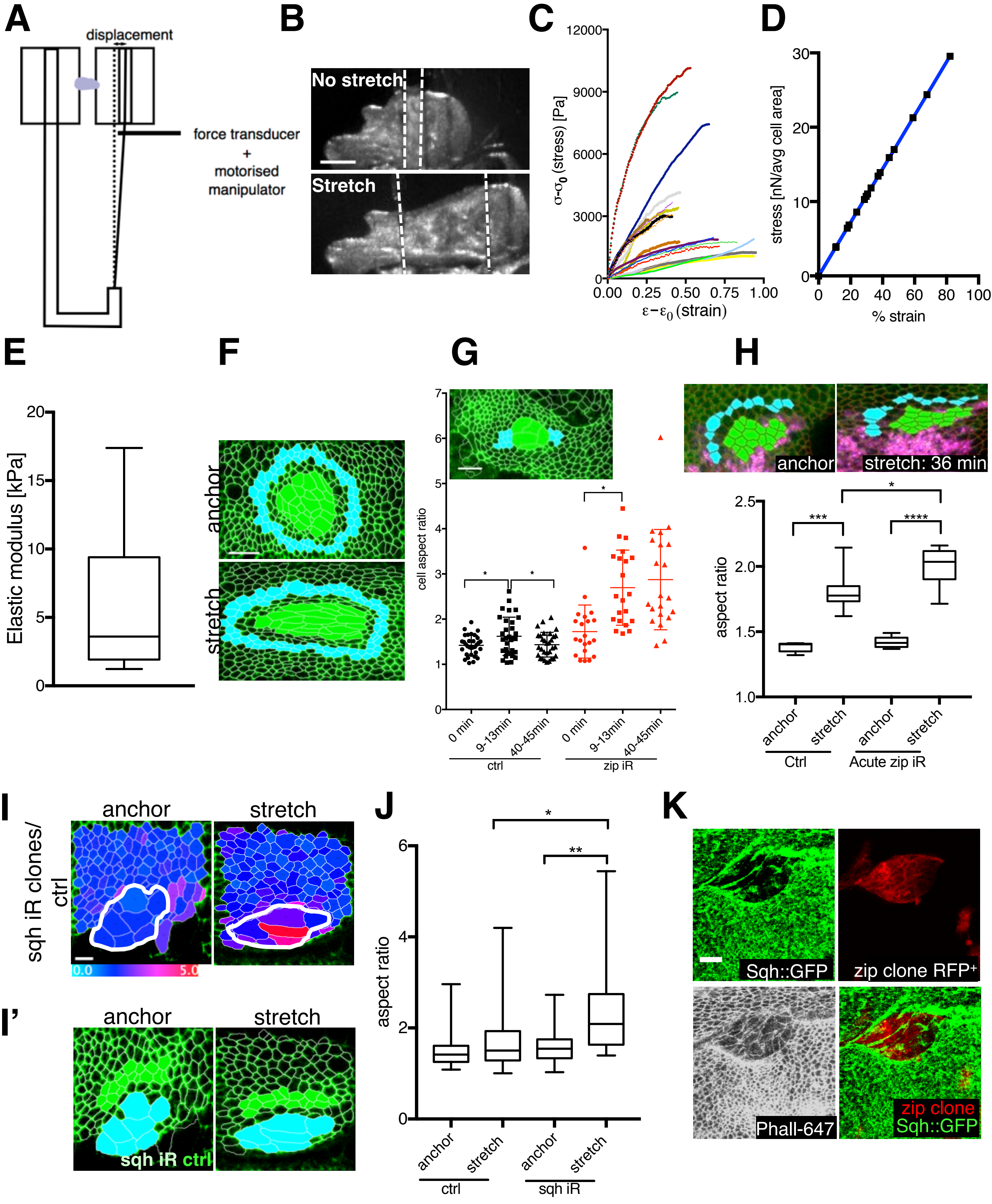

Figure S1, related to Figure 1. Further characterization of forces applied and *zip* RNAi cells in stretching assays.

(A) Schematics of the force transducer set up: wing (purple) is attached to two coverslips glued on a stiff and elastic rod. The elastic rod is directly connected to the force transducer and motorised manipulator, which stretches the tissue.

(B) Brightfield image of wing disc prior to and after stretch.

(C) Stress (y axis)-strain (x axis) curves of each stretching experiment. N=15 discs, 3 different devices (as shown in A).

(D) Forces experienced by the average cell area (10 μm^2) with different degrees of stretching (quantified as % strain change; strains are comparable to assay shown in (C).

(E) Box plot showing distribution of derived Young's modulus values; median (3.6.kPa) is indicated with horizontal line and used thereafter for force calculations.

(F) Segmentation example of a *zip*-RNAi clone as in Fig. 1E. Selection of control (cyan) and *zip*-RNAi (green) cells. Note a 2-cell rows gap is always ensured between clone and control cells in order to avoid the unspecific effects from clone stretching.

(G) Distribution plot showing aspect ratio change in discs subjected to constant deformation for 40-45 min for control and *zip*-RNAi cells (image shows selection of control cells (cyan) directly adjacent and in series with the *zip*-RNAi clone (green) and aligned with direction of the stretch); X-axis defines specific timepoints during stretch (in minutes); 0 min indicates anchored (unstretched) discs. N=3 discs; error bars represent S.D.

(H) Silent clone system with 16 hours *zip*-RNAi expression (marked by magenta) in third instar wing discs expressing Arm::GFP subject to up to 40 min stretch. Overlaid segmentation with control (cyan) and *zip*-RNAi (green). Box plot showing distribution of cell aspect ratio in anchored and stretched (20 min) discs with acute *zip*-RNAi; median is represented by horizontal line; 75th and 25th centiles are represented by top and bottom of the boxes respectively, n= 5-7 wing discs.

(I) Color coded aspect ratio of anchored and stretched (20 min) disc with *sqh*-RNAi clone (labeled as *sqh* iR and outlined in white).

(I') Control cells (green) and *sqh*-RNAi cells (cyan) prior to and after stretch (20 min).

(J) Box plot showing distribution of cell aspect ratio in anchored and stretched (20 min) wing disc for control (wild-type) and *sqh*-RNAi cells; median is represented by horizontal line; 75th and 25th centiles are represented by top and bottom of the boxes respectively, n= 3 wing discs.

(K) Reduction of Sqh::GFP signal in *zipper* RNAi clone (labeled as *zip* iR, in red).

*$p<0.05$, **$p<0.01$ with *t*-test. Scale bars, 5 μm (K), 10 μm (F,G,I,I'), 150 μm (B).

Duda et al. Fig. S2 related to Figure 2

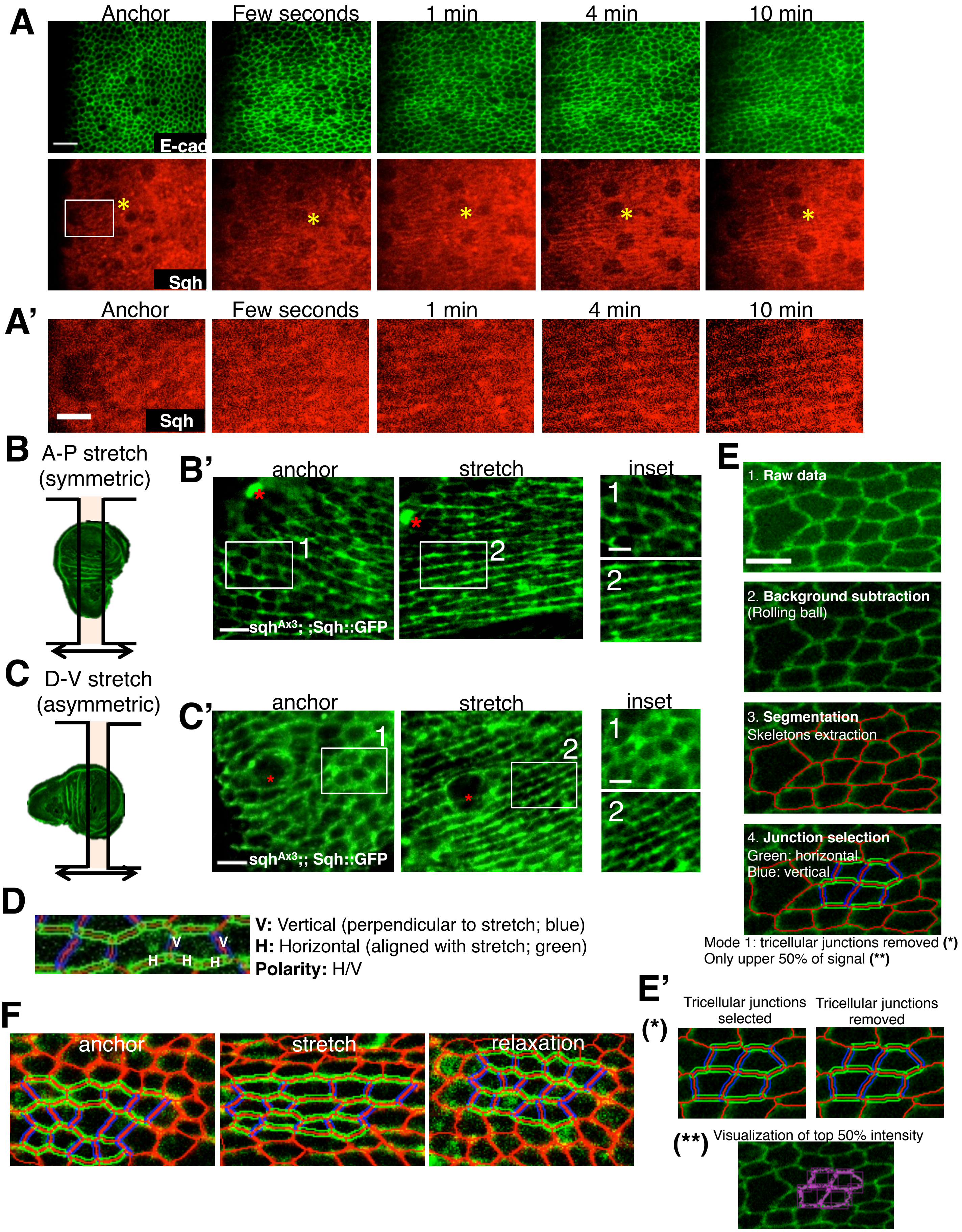

Figure S2, related to Figure 2. Emergence of Sqh polarity and Quantification of polarity.

(A) Emergence of Myosin polarity following mechanical stretching in discs expressing Sqh:mCherry and E-cad::GFP. (A') Inset of Sqh::mCherry from experiment described in (A).

(B) Schematics showing symmetric stretching, defined as stretch along anterior (A)-posterior (P) axis. (B') Discs expressing Sqh::GFP in sqh^{Ax3} null background subjected to symmetric stretch. Insets show a close up of equivalent regions prior to (1) and after stretch (2).

(C) Schematics showing asymmetric stretching, defined as stretch along dorsal (D)-ventral (V) axis. (C') Sqh::GFP in sqh^{Ax3} null background subjected to asymmetric stretch. Insets show a close up of equivalent regions prior to (1) and after stretch (2).

(D) Example of vertical (blue) and horizontal junctions (green). Vertical junctions are at angle > 45° to the direction of stretch while horizontal junctions are aligned <45° with respect to the direction of stretch (and equivalent axis in unstretched tissue). Polarity is defined as the enrichment of mean intensity on horizontal junctions with respect to mean intensity on vertical junctions.

(E) Steps of fluorescent intensity measurements: raw data is subjected to background subtraction and segmentation with EpiTools, junctions are categorized as vertical or horizontal (if unsure, junction omitted) and pixels in the 50% top intensity (**) are measured in mode 1 (tricellular signal removed (*)); note that extracted intensity overlaps perfectly with junctional geometry.

(F) Example of junction selection in anchored, stretched and relaxed disc; green junctions are defined as horizontal junctions, blue junctions are vertical junctions.

Red and yellow asterisks indicate equivalent regions in the tissue. Scale bars, 10 μm (A), 5 μm (A',B',C', E), 3 μm (B' inset, C' inset).

Duda et al. Fig. S3 related to Figure 4

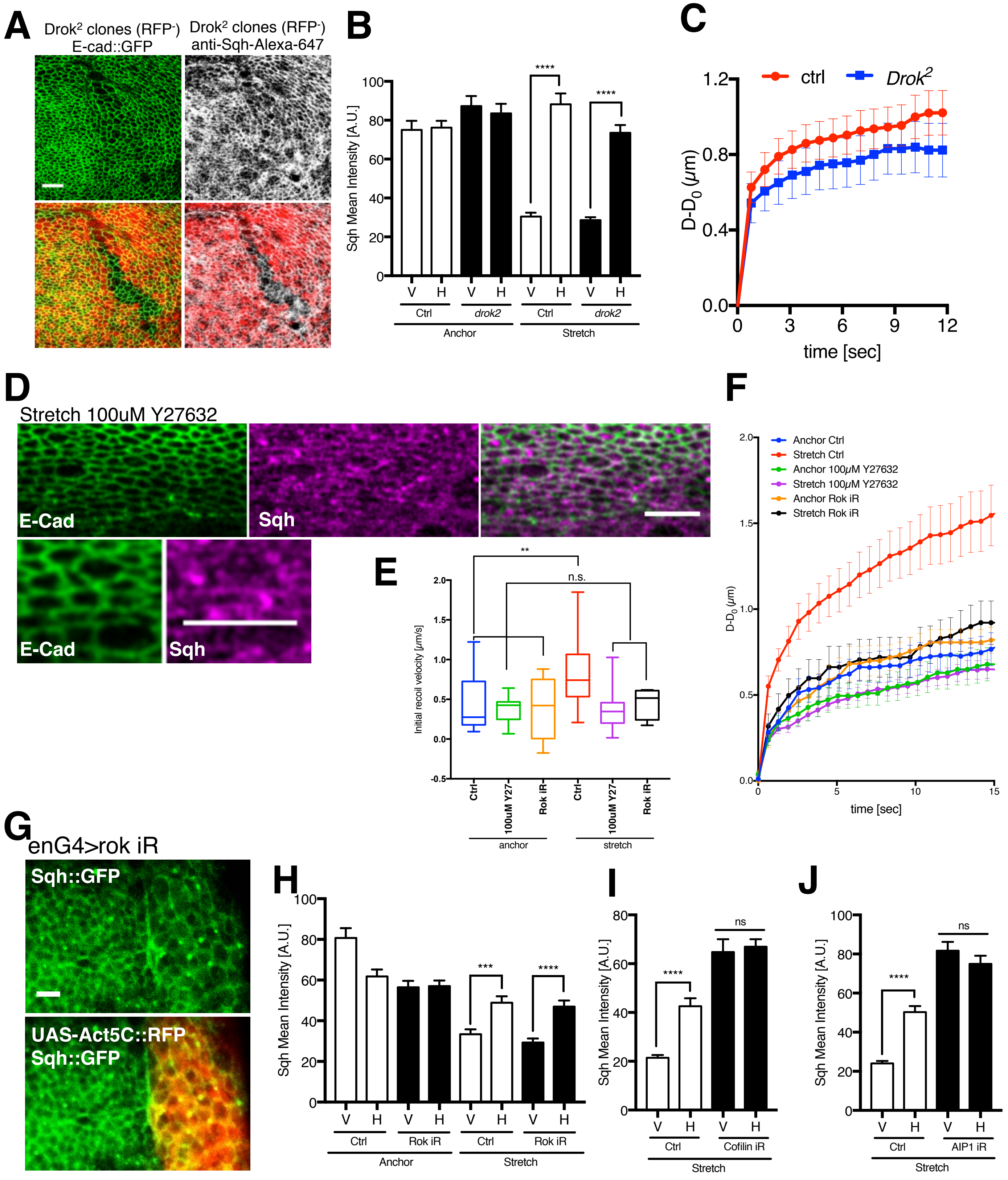

Figure S3, related to Figure 4. Characterization of *Rok* depletion.

(A) *Drok²* clones in Arm::GFP expressing wing disc stained with anti - Sqh (Alexa 647, red) antibody showing enlarged cell size and junctional Sqh inside the clone.

(B) Quantification of junctional Sqh mean intensity in experiment (A) for anchor and stretch, control and *Drok²*; error bars represent S.E.M.

(C) Laser ablations of junctions in *Drok²* clones and control cells in Arm::GFP disc. Plot shows increase in distance between recoiling vertices [μm] against time [sec.]; error bars represent S.E.M.

(D) Rok inhibitor Y27632 treated discs expressing ECad::GFP and Sqh::Cherry under stretch.

(E) Single junction ablation initial recoil velocity for Rok inhibitor treated and *rok*-RNAi wing discs; error bars represent S.E.M.

(F) Laser ablations of junctions used for calculation of (E). Plot shows increase in distance between recoiling vertices [μm] against time [sec.]; error bars represent S.E.M.

(G) UAS- *rok*-RNAi expressed from enGal4 driver (posterior side marked by UAS-Act5C::RFP in red) in Sqh::GFP wing disc shows junctional Sqh as well as enlarged cell size of *rok* iR cells.

(H) Quantification of Sqh junctional intensity for experiment described for (G) in anchored and stretched discs; error bars represent S.E.M.

(I) Quantification of stretched discs expressing Sqh::GFP and *enGal4>UAS-cofilin RNAi* and *UAS-Act5c-RFP*, presented in FigS4C; error bars represent S.E.M.

(J) Quantification of stretched discs expressing Sqh::GFP and *enGal4>UAS-AIP1 RNAi* and *UAS-Act5c-RFP*, presented in FigS4C; error bars represent S.E.M.

Scale bars 10 μm.

Duda et al. Fig. S4 related to Figure 4

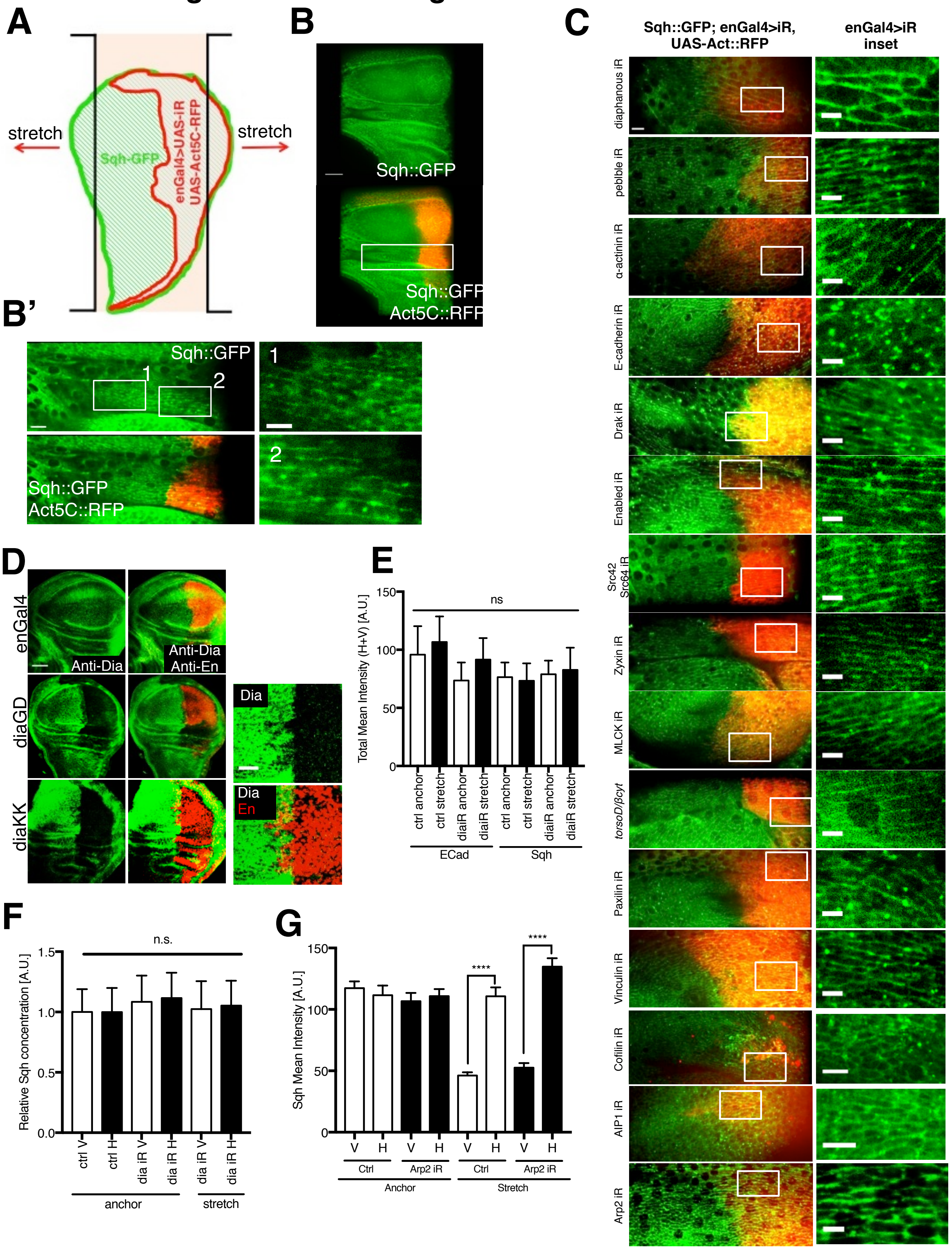

Figure S4, related to Figure 4. Screen identifies MyoII polarity is induced downstream of Diaphanous.

(A) Schematics representing screen rationale: wing disc expressing Sqh::GFP and RNAi of interest using *en*Gal4 driver (*en*Gal4 marked by UAS-Act5C::RFP expression) is subjected to stretch; the readout of the screen is comparison of Sqh polarity on anterior (control) and posterior (RNAi/overexpression) side.

(B) Stretched *Sqh::GFP, enGal4> UAS-Act5C::RFP* wing disc.

(B') Close up of disc shown in (B) showing Sqh:GFP polarity in anterior (inset 1) and posterior (inset 2, marked in red) wing disc compartment.

(C) Screen candidates and observations using system described in A and B. Insets show Sqh::GFP in posterior compartment, scale bars, 5μm.

(D) Diaphanous antibody staining (green) in discs expressing *en*Gal4> UAS-*dia*-RNAi (GD VDRC library, labeled as diaGD) and *en*Gal4>*dia*-RNAi (KK VDRC library, labeled as diaKK). Discs are stained with DAPI (blue), Phalloidin-647 (white) and anti-Engrailed (red, marks posterior side). D inset shows strength of enGal4 driver is patchy around anterior – posterior boundary: some Engrailed positive cells (red) do not have visible *diaphanous* knock-down (green).

(E) Total myosin levels calculated from experiment in Fig4F in anchor and stretch with wildtype anterior and *dia*-RNAi expression in the posterior of the wing disc; error bars represent S.E.M.

(F) Relative change in junctional Sqh concentration in anchor and stretch wing discs described in (A) that are expressing *dia*-RNAi in the posterior compartment and are wild type in the anterior compartment. Quantification from discs shown in (Fig4F). Values normalized to anchored vertical junction concentration, n=4; error bars represent S.E.M.

(G) Quantification of stretched discs expressing Sqh::GFP and *enGal4>UAS-Arp2 RNAi* and *UAS-Act5c-RFP*, presented in (C); error bars represent S.E.M.

****$p<0.0001$ with *t*-test. Scale bars, 50 μm (D), 30 μm (B), 10 μm (B'), 5 μm (B' and C insets)

Duda et al. Fig S5 related to Figure 5

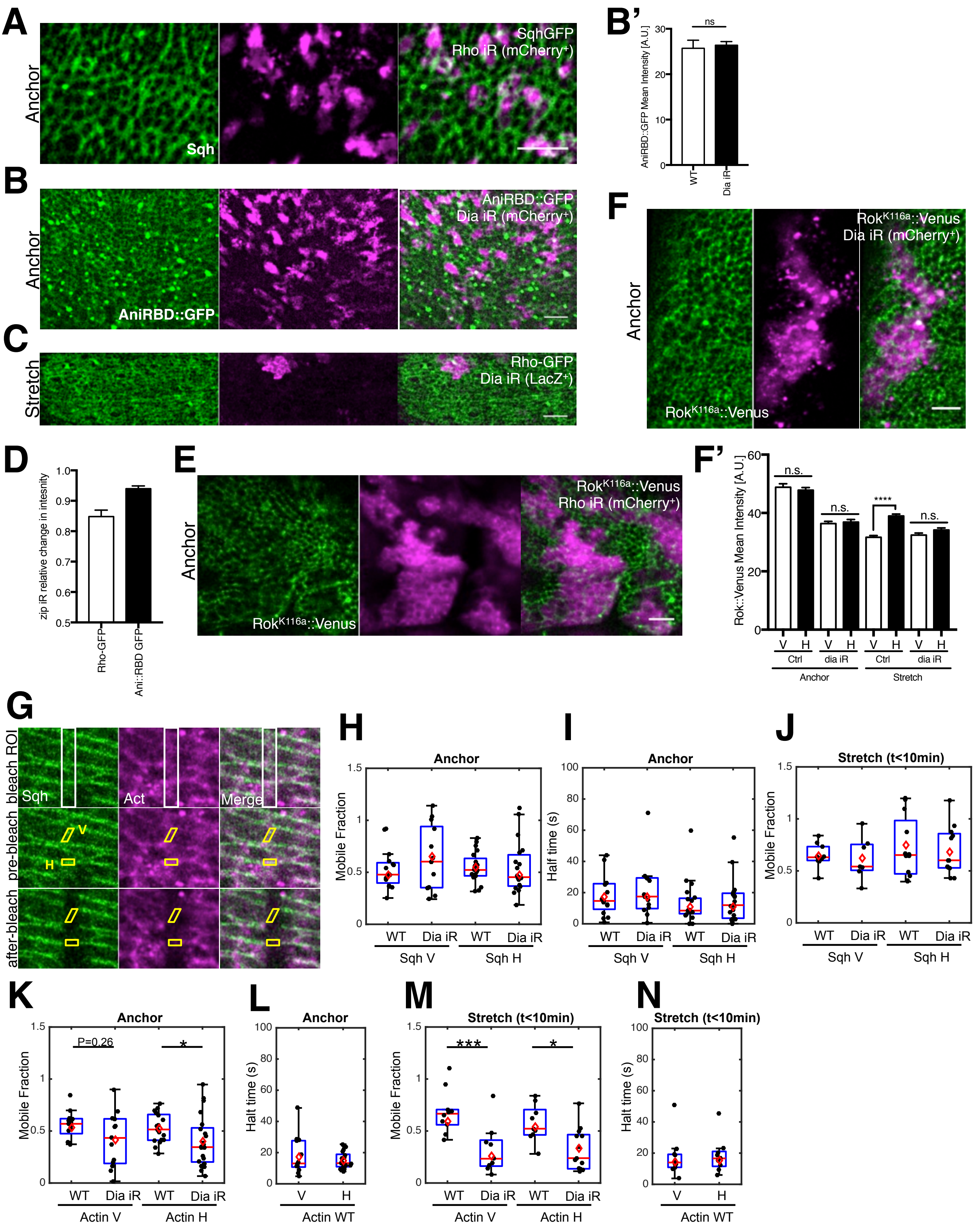

Figure S5, related to Figure 5. Mechanism of Diaphanous in MyoII polarity

(A) *rho*-RNAi clones (indicated in magenta) in anchored Sqh::GFP expressing discs.

(B) *dia*-RNAi clones (indicated in magenta) in anchored AniRBD::GFP expressing discs. (B') quantification of AniRBD::GFP junctional mean intensity in wildtype regions and *dia*-RNAi clones; error bars represent S.E.M.

(C) *dia*-RNAi clones (indicated in magenta) in stretched Rho::GFP protein trap expressing discs. Discs were fixed under stretch and stained, Rho::GFP boosted with anti-GFP-FITC.

(D) Quantification of the relative change in junctional intensity for AniRBD::GFP and Rho::GFP protein trap *zip*-RNAi clones compared to wildtype region; error bars represent S.E.M.

(E) *rho*-RNAi clones (indicated in magenta) in anchored Rok^{K116a}::Venus expressing discs.

(F) *dia*-RNAi clones (indicated in magenta) in anchored Rok^{K116a}::Venus expressing discs. (F') Quantification of Rok^{K116a}::Venus mean junctional intensity for horizontal and vertical junctions in anchored and stretched discs expressing *dia*-RNAi clones, n=3, ****$p<0.0001$ with *t*-test.

(A-F): scale bar 10μm; error bars represent S.E.M.

(G) Fluorescence recovery after photo-bleaching diagram of bleaching and junction analysis. White bounding box shows the bleach region and the yellow boxes mark the horizontal and vertical junctions analyzed for recovery as described in experimental procedures. Wing discs were used as described in (FigS4A) and that co-expressed UAS *dia*-RNAi.

(H-I) FRAP mobile fraction and half time of Sqh::GFP on horizontal and vertical junctions in anchored discs expressing either Sqh::GFP, enGal4> UAS-Act5CRFP or Sqh::GFP, enGal4>UAS-dia RNAi, UAS-Act5CRFP.

(J) FRAP mobile fraction of Sqh::GFP on horizontal and vertical junctions in discs expressing either Sqh::GFP, enGal4> UAS-Act5CRFP or Sqh::GFP, enGal4>UAS-dia RNAi, UAS-Act5CRFP subjected to <10 min stretch.

(K-L) FRAP mobile fraction and half time of Act5C::RFP on horizontal and vertical junctions in anchored discs expressing either Sqh::GFP, enGal4> UAS-Act5CRFP or Sqh::GFP, enGal4>UAS-dia RNAi, UAS-Act5CRFP.

(M-N) FRAP mobile fraction and half time of Act5C::RFP on horizontal and vertical junctions in discs expressing either Sqh::GFP, enGal4> UAS-Act5CRFP or Sqh::GFP, enGal4>UAS-dia RNAi, UAS-Act5CRFP subjected to <10 min stretch.

In all boxplots (H-N), red line indicates the median, red diamond the mean, edges the $25^{th}/75^{th}$ percentile, and whiskers the most extreme data points that are not considered to be outliers. Black points represent individual junctions. * $p<0.05$, ***$p<0.001$ with Wilcoxon rank-sum test.

Duda et al. Fig.S6 related to Figure 6

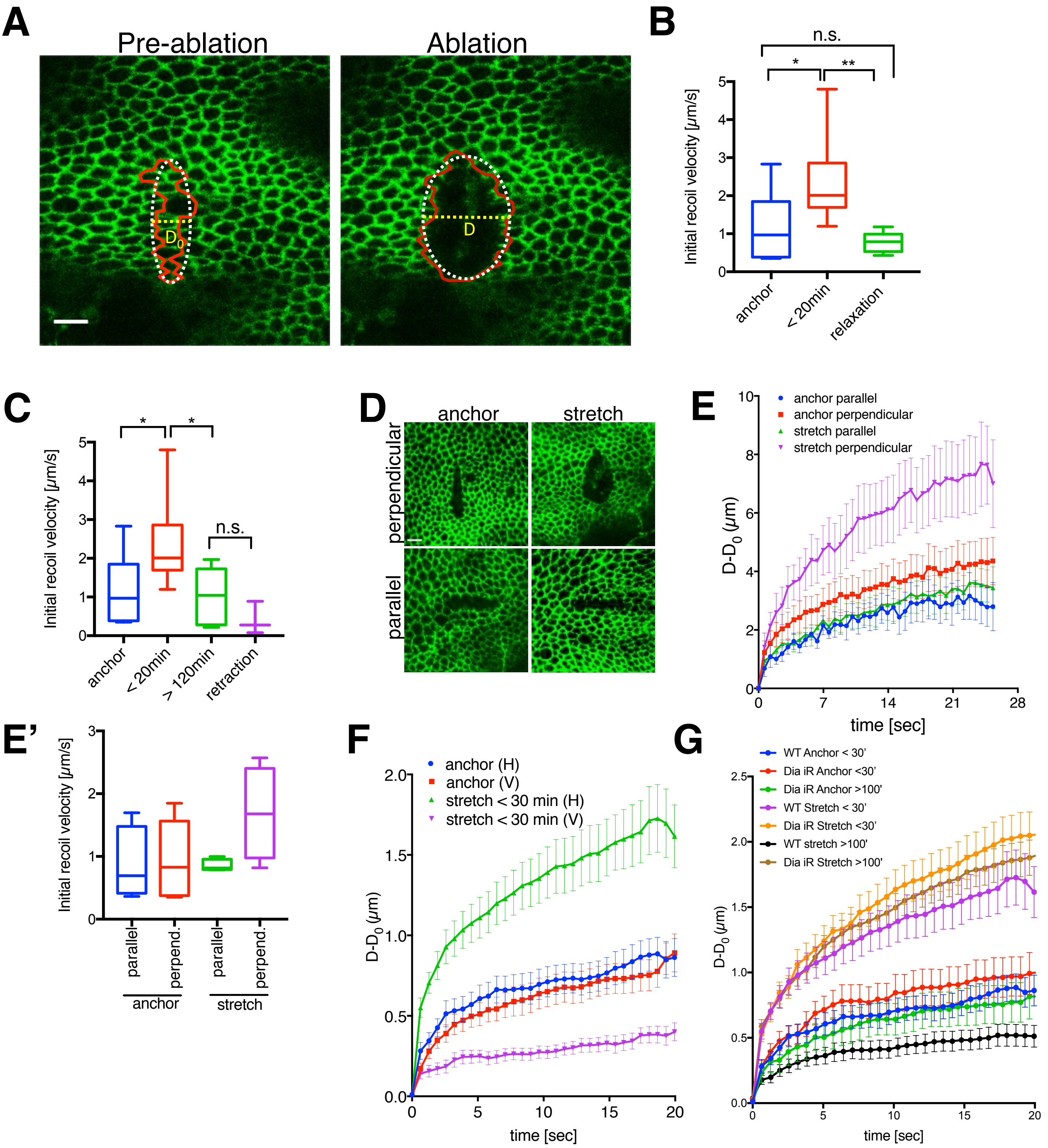

Figure S6, related to Figure 6. Cell- and tissue-scale tension measurements in stretched tissue.

(A) Stretched tissue prior to (left panel) and after (right panel) ablation of circa 10 cells in the direction perpendicular to stretch. Red line outlines ablated area; white dotted line represents ellipse fitted to ablation area; yellow dotted line indicates short ellipse axis measuring ablation recoil.

(B, C) Box plots of initial recoil velocity quantified as a short ellipse length change ($D\text{-}D_0$) over time of 1.27 seconds post-ablation (first timepoint was omitted due to sporadic unspecific effects from extensive injury) as described in (A). Median is represented by horizontal line; 75th and 25th percentiles are represented by top and bottom of the boxes respectively. Plot in (B) represents experiment where discs were anchored, stretched (<20 min) and relaxed; plot in (C) shows the same data for anchored and stretched (<20 min) discs as in (B) but plotted together with discs stretched >120 min and retracted (compressed to initial shape) from Fig6A'. N=3-11 wing discs per condition.

(D) Laser cuts (perpendicular or parallel to the line of stretch) across circa 10 cells in E-cad::GFP expressing wing disc tissue subjected to anchoring or short stretching (< 30 min).

(E) Plot showing anchored and stretched (<20 min) discs ablated in the direction perpendicular and parallel to stretch as described in (D); data is plotted as a mean ± S.E.M. (E') Box plot of initial recoil velocity from (E) calculated as described for (B) and (C). N=3-11 wing discs per condition.

(F) Plot showing increase in distance between recoiling vertices ($D\text{-}D_0$) over time [sec.] following laser cuts of single horizontal (H) and vertical (V) junctions in anchored and stretched E-cad::GFP expressing discs. Data is plotted as a mean ± S.E.M, n=17-23 cuts per condition.

(G) Plot showing increase in distance between recoiling vertices ($D\text{-}D_0$) over time [sec.] following laser cuts of single horizontal (H) junctions in anchored, short term and long term stretched E-cad::GFP expressing discs, with and without *dia*-RNAi expression through rotundGal4 driver. Data is plotted as a mean ± S.E.M, n=8-30 cuts per condition and refers to initial recoil velocity presented in Fig6C.

* $p<0.05$, ** $p<0.01$ with *t*-test. Scale bars, 10 μm.

Duda et al. Fig.S7 related to Fig.6 and Fig. 7

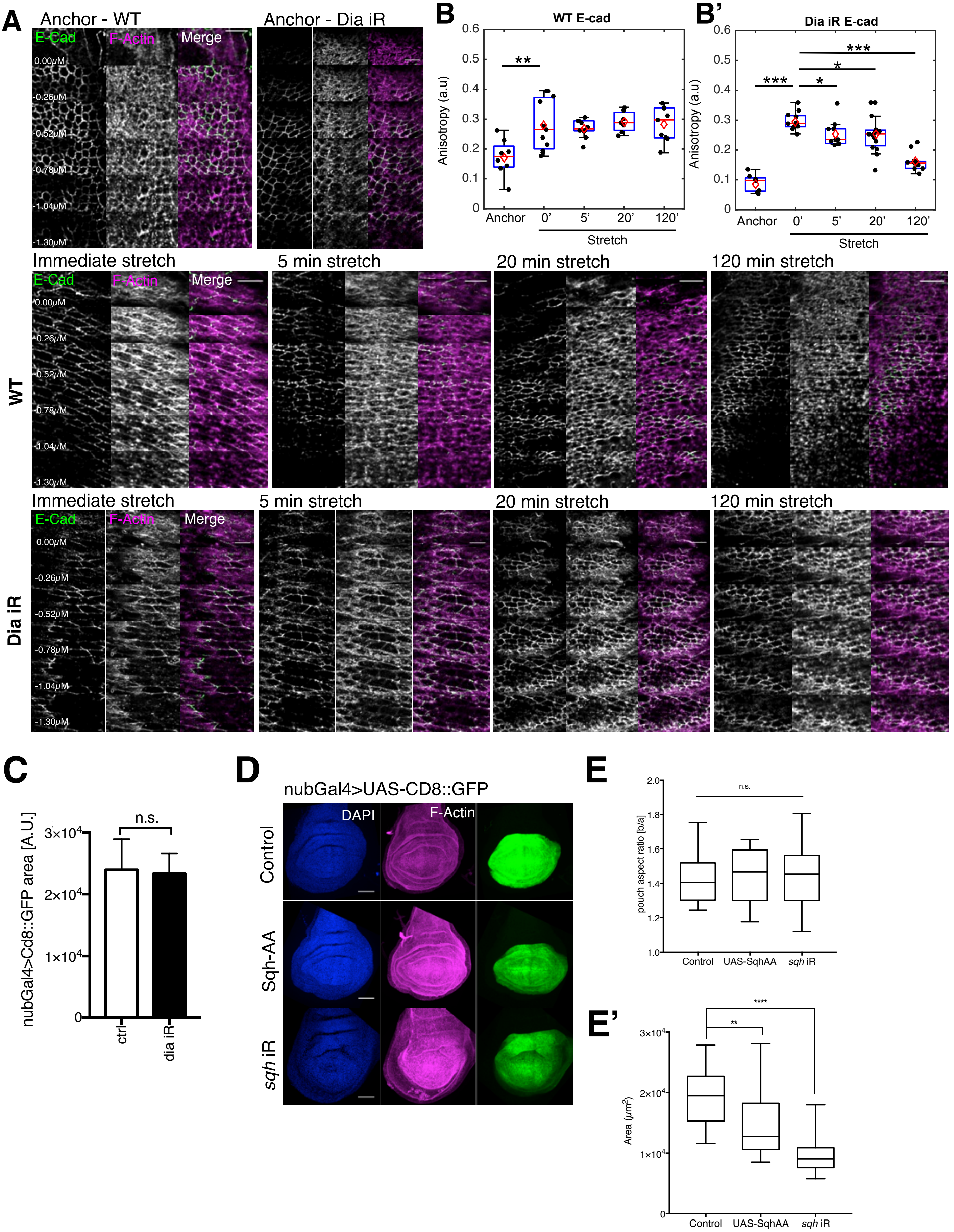

Figure S7, related to Figure 6, 7. Actin remodelling dynamics and disc shape analysis

(A) Wildtype discs expressing Ecad::6XRFP or Ecad::GFP;rnGal4>UAS-*dia*-RNAi were fixed whilst in anchored or stretched positions, then stained for filamentous actin with Phalloidin. Imaging was deconvolved and is presented in 0.26μm z steps to show junctional actin and apical meshwork (5μm scale bars).

(B) Anisotropy quantification as described in experimental procedures for Ecad in wildtype and *dia*-RNAi expressing (B') wing discs in (A). N=3-5. In all boxplots, red line indicates the median, red diamond the mean, edges the 25th/75th percentile, and whiskers the most extreme data points that are not considered to be outliers. Black points represent individual ROIs as described in experimental procedures. *p<0.05, **p<0.01 and ***p<0.001 with Wilcoxon rank-sum test.

(C) Mean wing pouch area quantified as total *nub*Gal4>Cd8::GFP (GFP positive) area in control condition (UAS-Cd8::GFP) and diaphanous knock-down (*dia* iR + UAS-Cd8::GFP) showing no apparent size difference. Error bars indicate standard deviation.

(D) Discs expressing *nub*Gal4 and Cd8::GFP (control) and UAS-*SqhAA* or *Sqh*-iR labeled with DAPI; ellipse is fitted into the pouch region and aspect ratio determined as long ellipse axis (b) divided by short ellipse axis (a) as described for Fig7G.

(E) Box plot showing distribution of wing disc pouch aspect ratios in conditions described in D.

(E') Box plot showing distribution of mean wing pouch area quantified as total *nub*Gal4>Cd8::GFP (GFP positive) area in control condition (UAS-Cd8::GFP) and myosin perturbation (RNAi + UAS-Cd8::GFP), described in (D,E). Median is represented by horizontal line; 75th and 25th percentiles are represented by top and bottom of the boxes respectively. *p<0.05, **p<0.01 ***p<0.002, ****p<0.0001, with *t*-test. Scale bars, 50μm (D) 5μm (A)
